# Multicenter phase II trial of Camrelizumab combined with Apatinib and Eribulin in heavily pretreated patients with advanced triple-negative breast cancer

**DOI:** 10.1038/s41467-022-30569-0

**Published:** 2022-05-31

**Authors:** Jieqiong Liu, Ying Wang, Zhenluan Tian, Ying Lin, Hengyu Li, Zhaowen Zhu, Qiang Liu, Shicheng Su, Yinduo Zeng, Weijuan Jia, Yaping Yang, Shengqiang Xu, Herui Yao, Wen Jiang, Erwei Song

**Affiliations:** 1 Guangdong Provincial Key Laboratory of Malignant Tumor Epigenetics and Gene Regulation, Breast Tumor Center, Sun Yat-sen Memorial Hospital, Sun Yat-sen University, Guangzhou, Guangdong China; 2 Department of Breast and Thyroid Surgery, First Affiliated Hospital of Sun Yat-sen University, Sun Yat-sen University, Guangzhou, Guangdong China; 3 Department of Breast and Thyroid Surgery, Changhai Hospital, Navy Medical University (Second Military Medical University), Shanghai, China; 4 YuceNeo, Shenzhen, Guangdong, China; 5 Department of Radiation Oncology, MD Anderson Cancer Center, Houston, TX USA

**Keywords:** Breast cancer, Breast cancer

## Abstract

In the later-line setting or for patients with PD-L1-negative tumors, immunotherapy-based regimens remain ineffective against advanced triple-negative breast cancer (TNBC). In this multicentered phase II trial (NCT04303741), 46 patients with pretreated advanced TNBC were enrolled to receive camrelizumab 200 mg (day 1), and apatinib 250 mg daily, plus eribulin 1.4 mg/m^2^ (day 1 and 8) on a 21-day cycle until progression, or unacceptable toxicity. Primary endpoint was objective response rate (ORR) according to RECIST 1.1. Secondary endpoints included toxicities, disease control rate (DCR), clinical benefit rate, progression-free survival (PFS), and 1-year overall survival. With a median of 3 lines of prior chemotherapy in the advanced setting, 17.4% had received PD-1/PD-L1 blockade plus chemotherapy for advanced disease. The ORR was 37.0% (17/46, 95% CI 23.2–52.5). The DCR was 87.0% (40/46, 95% CI 73.7–95.1). Median PFS was 8.1 (95% CI 4.6–10.3) months. Tertiary lymphoid structure was associated with higher ORR. Patients with lower tumor PML or PLOD3 expression had favorable ORR and PFS. PD-L1 status was not associated with ORR/PFS. Grade 3/4 treatment-related adverse events occurred in 19 (41.3%) of 46 patients. Camrelizumab plus apatinib and eribulin shows promising efficacy with a measurable safety profile in patients with heavily pretreated advanced TNBC.

## Abstract (web summary)

Therapeutic options for patients with triple-negative breast cancer (TNBC) in later-line setting are limited. Here the authors report the results of a phase 2 clinical trial to evaluate efficacy and safety of the combination of camrelizumab (anti-PD1), apatinib (VEGFR2 inhibitor), and eribulin in patients with heavily pre-treated advanced TNBC.

## Introduction

Triple-negative breast cancer (TNBC) is notorious of its early onset and poor prognosis, as well as short median overall survival (OS) once metastasis^1,2^. First-line immune checkpoint inhibitor in combination with chemotherapy has improved survivals in patients with programmed cell death-ligand 1 (PD-L1)-positive advanced TNBC^3,4^. However, in the later-line setting or for patients with PD-L1-negative tumors, immunotherapy-based regimens have not been reported to be effective^5,6^. According to the National Comprehensive Cancer Network (NCCN)^7^ and European Society of Medical Oncology (ESMO) Clinical Practice Guidelines^8^, single or double-agent chemotherapy and sacituzumab govitecan (a Trop-2-directed antibody-drug conjugate) are current recommended treatment options as the second or later-line therapy for patients with advanced TNBC. Nevertheless, the objective response rates (ORR) ranged between 5–26.6%^6,9,10^ with single-agent chemotherapy, 22.2–31.6% with doublet chemotherapy^11,12^ and 31.0% with sacituzumab govitecan^13^. The survival outcomes of patients treated with these drugs are also unsatisfactory, as the median progression-free survival (PFS) ranged between 1.7 and 5.6 months^10,11,13^. Thus, there is an unmet need for developing novel anti-tumor agents or treatment combinations for these pretreated patients with life-threatening advanced TNBC.

We recently showed that anti-angiogenic therapies with a low dose could increase the efficacy of anti-programmed cell death-1 (PD-1) therapy via normalizing blood vessel, increasing CD8^+^ T cells and B cells infiltration and PD-1 expression on immune cells in multiple mouse models^14^. In our prior phase 2 trial, camrelizumab (anti-PD-1 antibody) combined with apatinib (vascular endothelial growth factor receptor 2 [VEGFR2] tyrosine kinase inhibitor) exhibited a favorable ORR of 43.3% in patients with advanced TNBC who had received no more than two lines of chemotherapy in the advanced setting^15^. However, the median PFS remained short (3.7 months) with this chemo-free regimen. The results of EMBRACE and 301 trials revealed that, as the later-line therapy in advanced breast cancer including TNBC, eribulin monotherapy significantly prolonged PFS and OS compared to other common chemo-drugs^9,16^. Furthermore, there is preclinical evidence suggesting tumor vessel remodeling activity of eribulin^17^. In addition, checkpoint blockade in combination with anti-angiogenesis and chemotherapy has been reported to have promising antitumor-activities with tolerable adverse events in other solid tumors^18,19^. For instance, the IMpower150 trial demonstrated that atezolizumab combined with bevacizumab and chemotherapy significantly improved PFS and OS than bevacizumab plus chemotherapy in patients with metastatic nonsquamous non-small-cell lung cancer^19^. However, such a triplet rationale has not been previously studied in patients with breast cancer.

In this phase 2 trial, we show that combination of camrelizumab, apatinib, and eribulin is effective and tolerable in patients with heavily pretreated advanced TNBC.

## Results

### Patients and Treatment

**Fig. 1:**
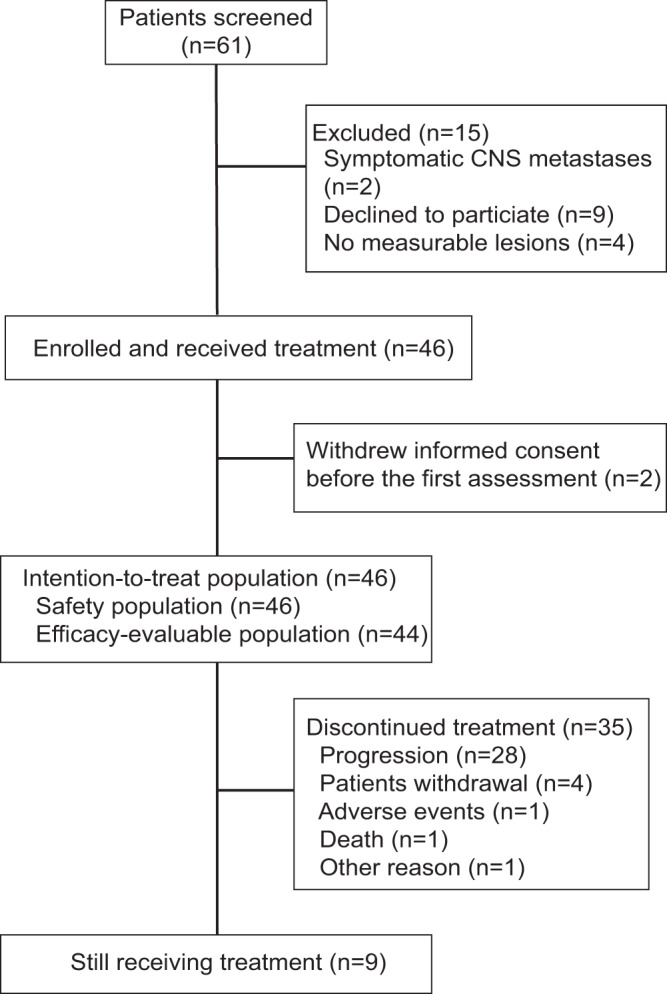
Trial profile. Treatment summary and data collection of study participants. Participants were recruited from 3 hospital sites in China.

From March 27, 2020, to May 27, 2021, 46 patients were enrolled from three academic hospitals in China. Safety analysis was evaluated in all patients (*n* = 46), while overall response of 44 (95.7%) patients were evaluable. Two patients discontinued study treatment before the first scheduled post-baseline assessment. As of the cut-off date of November 30, 2021, the median follow-up time was 11.2 (range, 4.4–20.2) months. At the time of analyses, 11 (23.9%) of the 46 patients had died, and nine (19.6%) patients were still on treatment (Fig. 1). Among all patients enrolled, 33 (71.8%) patients received at least two lines and 17 (37.0%) received at least three lines of treatment in the advanced setting before enrollment (Table 1).

**Table 1:** Baseline characteristics of study population (*N* = 46).

	Patients, No. (%)
**Median age, years (range)**	47 (30–65)
**ECOG performance status**	
0	16 (34.8)
1	30 (65.2)
**Metastatic disease**	42 (91.3)
**Interval from advanced disease diagnosis to enrollment, months (range)**	9.2 (0.6–69.2)
**Median No. of prior therapies (range)**	3 (2–10)
**Number of lines of prior therapies in the advanced setting**	
1	13 (28.3)
2	16 (34.8)
≥3	17 (37.0)
**Median No. of prior anticancer drugs type (range)**	5.5 (2–12)
**Sites of disease**^**a**^	
Chest	29 (63.0)
Liver	21 (45.7)
Lung	20 (43.5)
Bone	19 (41.3)
CNS	2 (4.3)
Pleural	2 (4.3)
Others^b^	2 (4.3)
**Number of metastatic sites**	
<3	23 (50.0)
≥3	23 (50.0)
**Liver metastasis**	
Yes	21 (45.7)
No	25 (54.3)
**Combined positive score** ≥ **1**	
Yes	36 (78.3)
No	8 (17.4)
Unknown	2 (4.3)
**Disease-free interval**^**c**^	
De novo^d^	12 (26.1)
<6 months	17 (37.0)
6≤DFI < 12 months	4 (8.7)
≥12 months	13 (28.2)
**Previous use of PD-1/PD-L1 antibodies**	
Yes	8 (17.4)
No	38 (82.6)

^a^Some patients had more than one metastasis.

^b^One patient had splenic metastasis by radiologic assessment, and one had adrenal metastasis.

^c^Disease-free interval was defined as the interval from completion of chemotherapy to the record of metastasis or recurrence.

^d^Patients with de novo diseases had received at least one standard chemotherapy regimen in the advanced setting before enrollment.

### Therapeutic efficacy

**Fig. 2:**
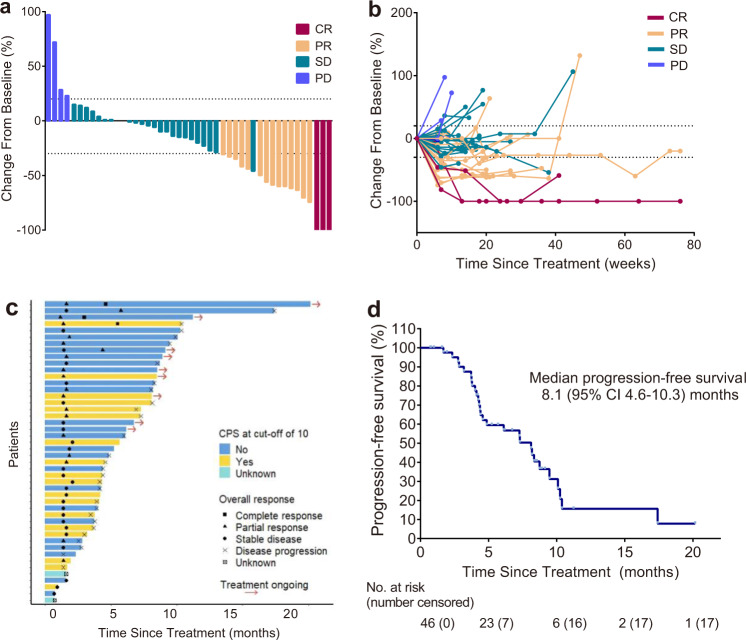
Efficacy evaluation. **a** Waterfall plot of best percent change in tumor size from baseline in the efficacy-evaluable population (*n* = 44). **b** Changes in tumor burden from baseline in the efficacy-evaluable population (*n* = 44). **c** Swimmer plot of duration of treatment (*n* = 46). **d** Kaplan–Meier curves of PFS (*n* = 46). Source data are provided as a Source Data file.

In the first stage, the overall response was observed in five patients (*n* = 14). Then 32 patients were enrolled in the second stage, with 30 had available response evaluation. Among intention-to-treat population, the ORR was 37.0% (17/46, 95% CI 23.2–52.5). Three (6.5%) and 14 (30.4%) patients had a best response of CR or PR, respectively, while 23 (50.0%) had SD, only four (8.7%) patients had progressive disease (PD) (Table 2; Fig. 2a–c). The DCR was 87.0% (40/46, 95% CI 73.7–95.1). And the CBR was 50.0% (23/46, 95% CI 34.9–65.1). Among the efficacy-evaluable population (*n* = 44), the ORR was 38.6% (17/44, 95% CI 24.4–54.5), the DCR was 90.9% (40/44, 95% CI 78.3–97.5), and the CBR was 52.3% (23/44, 95% CI 36.7–67.5) (Table 2).

**Table 2:** Tumor response.

	Intention-to-treat population (*n* = 46)	Efficacy-evaluable population (*n* = 44)
**Best overall response**		
CR	3 (6.5)	3 (6.8)
PR	14 (30.4)	14 (31.8)
SD	23 (50.0)	23 (52.3)
PD	4 (8.7)	4 (9.1)
Not evaluable^a^	2 (4.3)	
**ORR**	17 (37.0)	17 (38.6)
95% CI	23.2–52.5	24.4–54.5
**DCR**	40 (87.0)	42 (90.9)
95% CI	73.7–95.1	78.3–97.5
**CBR**	23 (50.0)	23 (52.3)
95% CI	34.9–65.1	36.7–67.5

Data are n (%), unless otherwise stated.

^a^Two patients discontinued study treatment before the first scheduled post-baseline assessment.

The median PFS was 8.1 (95% CI 4.6–10.3) months (Fig. 2d), and the median OS was not reached. The 1-year OS rate was 68.3% (95% CI 54.3-85.9). The median DoR and TTR were 8.6 (95% CI 6.0-not reached) months and 1.5 (range 1.3–4.5) months, respectively.

Among the 46 patients enrolled, eight patients (17.4%) had received anti-PD-1 antibody combined with chemotherapy in the advanced stage. Two of eight patients had achieved PR, and the ORR of these eight patients was 25.0% (95% CI 3.2–65.1) (Supplementary Table 2). Five of eight patients (62.5%) assessed as SD, while one of eight (12.5%) had PD (Supplementary Table 2). The CBR of these eight patients was 25.0% (2/8, 95% CI 3.2-65.1), and the median PFS was 4.2 (95% CI 3.8-not reached) months (Supplementary Table 2).

For exploratory subgroup analysis, the ORR and PFS were generally consistent across different subgroups, including those that were defined by the presence of liver metastasis, prior lines of therapy, disease-free interval, and CPS score (Supplementary Table 3). However, patients with less than three metastatic sites had a longer median PFS (8.7 months, 95% CI 6.1-not reached) as compared with those with three or more metastatic sites (5.8 months, 95% CI 4.1-not reached).

### Safety

All patients (*n* = 46) in the ITT population experienced treatment-related adverse events (TRAEs) of any grade. The most common TRAEs were elevated aspartate aminotransferase (74.0%), elevated alanine transaminase (65.2%), leukopenia (65.2%), hand-foot syndrome (54.3%), neutropenia (52.2%), alopecia (41.3%), and fatigue (39.1%). Grade 3/4 TRAEs occurred in 19 (41.3%) of 46 patients, with the most common being neutropenia (30.4%), thrombocytopenia (19.6%), elevated aspartate aminotransferase (17.4%), elevated alanine transaminase (17.4%), and leukopenia (13.0%) (Table 3).

**Table 3:** Treatment-related adverse events.

	*No*. (%)					
	All Grade	Grade 3 or 4	Grade 1	Grade 2	Grade 3	Grade 4
Elevated AST	34 (74.0)	8 (17.4)	10 (21.7)	16 (34.8)	8 (17.4)	0
Elevated ALT	30 (65.2)	8 (17.4)	5 (10.9)	17 (37.0)	8 (17.4)	0
Leukopenia	30 (65.2)	6 (13.0)	6 (13.0)	18 (39.1)	4 (8.7)	2 (4.3)
Hand-foot syndrome	25 (54.3)	3 (6.5)	14 (30.4)	8 (17.4)	3 (6.5)	0
Neutropenia	24 (52.2)	14 (30.4)	4 (8.9)	6 (13.0)	9 (19.6)	5 (10.9)
Alopecia	19 (41.3)	0	10 (21.7)	9 (19.6)	0	0
Fatigue	18 (39.1)	0	10 (21.7)	8 (17.4)	0	0
Thrombocytopenia	16 (34.8)	9 (19.6)	3 (6.5)	4 (8.7)	7 (15.2)	2 (4.3)
Rash	16 (34.8)	2 (4.3)	4 (8.7)	10 (21.7)	2 (4.3)	0
Canker sore	11 (23.9)	0	5 (10.9)	6 (13.0)	0	0
Anorexia	10 (21.7)	0	5 (10.9)	5 (10.9)	0	0
Gingivitis	8 (17.4)	0	7 (15.2)	1 (2.2)	0	0
Lose weight	8 (17.4)	0	6 (13.0)	2 (4.3)	0	0
Pneumonia	8 (17.4)	1 (2.2)	1 (2.2)	6 (13.0)	1 (2.2)	0
Voice hoarse	8 (17.4)	0	8 (17.4)	0	0	0
Diarrhea	7 (15.2)	0	5 (10.9)	2 (4.3)	0	0
Hypertension	7 (15.2)	0	2 (4.3)	5 (10.9)	0	0
Hypothyroidism	7 (15.2)	1 (2.2)	3 (6.5)	3 (6.5)	1 (2.2)	0
Proteinuria	7 (15.2)	0	5 (10.9)	2 (4.3)	0	0
Capillary hemangioma	7 (15.2)	0	6 (13.0)	1 (2.2)	0	0
Insomnia	6 (13.0)	0	2 (4.3)	4 (8.7)	0	0
Fever	6 (13.0)	1 (2.2)	3 (6.5)	2 (4.3)	1 (2.2)	0
Elevated bilirubin	6 (13.0)	3 (6.5)	1 (2.2)	2 (4.3)	3 (6.5)	0
Pain	6 (13.0)	1 (2.2)	0	5 (10.9)	1 (2.2)	0
Elevated CK-MB	5 (10.9)	0	4 (8.7)	1 (2.2)	0	0
Hypoalbuminema	5 (10.9)	0	1 (2.2)	4 (8.7)	0	0
Hemoglobin reduction	5 (10.9)	2 (4.3)	2 (4.3)	1 (2.2)	2 (4.3)	0
Constipation	4 (8.7)	0	3 (6.5)	1 (2.2)	0	0
Hemoglobinuria	4 (8.7)	0	4 (8.7)	0	0	0
Headache	4 (8.7)	0	3 (6.5)	1 (2.2)	0	0
Hydropericardium	3 (6.5)	1 (2.2)	2 (4.3)	0	1 (2.2)	0
Infusion reaction	3 (6.5)	0	0	3 (6.5)	0	0
Stomachache	3 (6.5)	0	2 (4.3)	1 (2.2)	0	0
Hyperthyroidism	2 (4.3)	0	0	2 (4.3)	0	0
Blurred vision	2 (4.3)	0	1 (2.2)	1 (2.2)	0	0

*AST* aspartate aminotransferase, *ALT* alanine transaminase, *CK-MB* creatine phosphokinase-MB.

A total of 40 (87.0%) patients had TRAEs considered to be related to immunotherapy. The most common symptoms were elevated aspartate aminotransferase (74.0%), elevated alanine transaminase (65.2%), leukopenia (65.2%), hand-foot syndrome (54.3%), neutropenia (52.2%), alopecia (41.3%) and fatigue (39.1%). Among them, three (6.5%) patients developed grade 3 bilirubin elevation (Supplementary Table 4) and one (2.2%) patient had elevated creatine kinase-MB that required prednisone treatment (Table 4).

**Table 4:** Immune-related adverse events.

	*No*. (%)					
	All Grade	Grade 3 or 4	Grade 1	Grade 2	Grade 3	Grade 4
Elevated AST	34 (74.0)	8 (17.4)	10 (21.7)	16 (34.8)	8 (17.4)	0
Elevated ALT	30 (65.2)	8 (17.4)	5 (10.9)	17 (37.0)	8 (17.4)	0
Leukopenia	30 (65.2)	6 (13.0)	6 (13.0)	18 (39.1)	4 (8.7)	2 (4.3)
Hand-foot syndrome	25 (54.3)	3 (6.5)	14 (30.4)	8 (17.4)	3 (6.5)	0
Neutropenia	24 (52.2)	14 (30.4)	4 (8.9)	6 (13.0)	9 (19.6)	5 (10.9)
Alopecia	19 (41.3)	0	10 (21.7)	9 (19.6)	0	0
Fatigue	18 (39.1)	0	10 (21.7)	8 (17.4)	0	0
Elevated bilirubin	6 (13.0)	3 (6.5)	1 (2.2)	2 (4.3)	3 (6.5)	0
Pneumonia	8 (17.4)	1 (2.2)	1 (2.2)	6 (13.0)	1 (2.2)	0
Diarrhea	7 (15.2)	0	5 (10.9)	2 (4.3)	0	0
Proteinuria	7 (15.2)	0	5 (10.9)	2 (4.3)	0	0
Hypothyroidism	7 (15.2)	1 (2.2)	3 (6.5)	3 (6.5)	1 (2.2)	0
Capillary hemangioma	7 (15.2)	0	6 (13.0)	1 (2.2)	0	0
Elevated CK-MB	5 (10.9)	0	4 (8.7)	1 (2.2)	0	0
Hyperthyroidism	2 (4.3)	0	0	2 (4.3)	0	0

*AST* aspartate aminotransferase, *ALT* alanine transaminase, *CK-MB* creatine phosphokinase-MB.

Overall, seven (15.2%) of the 46 patients had a dose suspension of camrelizumab due to TRAEs (Supplementary Table 5). Moreover, 13 (28.3%) patients had a dose reduction of apatinib or eribulin due to TRAEs (Supplementary Table 5). One patient discontinued apatinib because of apatinib-related toxicity and withdrew from the study permanently. Furthermore, three (6.5%) of 46 patients exhibited treatment-related serious AEs (SAEs). They were bone marrow suppression (*n* = 1, 2.2%), elevated aspartate aminotransferase (*n* = 1, 2.2%), and thrombosis (*n* = 1, 2.2%). No treatment-related death occurred.

### Potential biomarkers

**Fig. 3:**
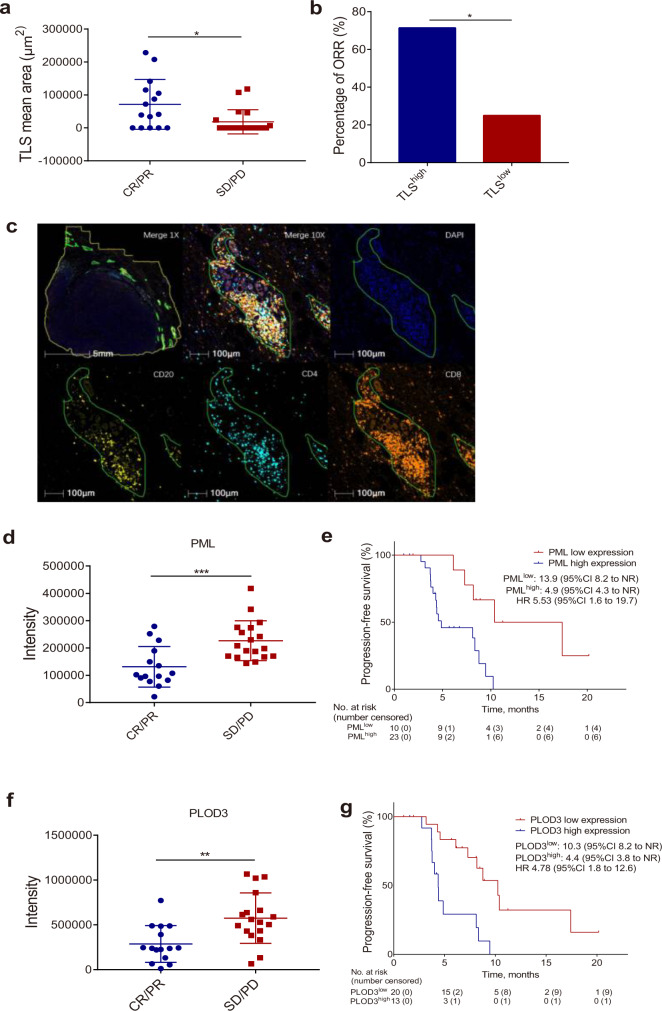
Biomarker analysis of TLS and proteomics. **a** Association between ORR and mean area of TLS, patients with SD/PD (*n* = 19) and with CR/PR (*n* = 15) biologically independent samples. Calculated by two-tailed *t*-test, *P* = 0.011. **b** Association between ORR (%) and mean area of TLS at cutoff value of 30,000 μm^2^. Calculated by two-tailed Fisher’s exact test, *P* = 0.014. **c** Presentative images of TLS stained by multiplex immunofluorescence using markers DAPI, CD20, CD4, and CD8. Original magnification, 1× or 10×. TLS staining was performed one time in 34 independent samples with similar results. **d** Association between response and PML expression level, patients with SD/PD (*n* = 18) and with CR/PR (*n* = 15) biologically independent samples. Calculated by two-tailed *t*-test, *P* = 0.0008. Each dot represents one patient. **e** Kaplan–Meier estimates of PFS by PML intensity at cutoff value of 139,189 (PML^high^ vs. PML^low^). NR, not reached. **f** Association between response and PLOD3 expression level, patients with SD/PD (*n* = 18) and with CR/PR (*n* = 15) biologically independent samples. Calculated by two-tailed *t*-test, *P* = 0.003. Each dot represents one patient. **g** Kaplan–Meier estimates of PFS by PLOD3 intensity at cutoff value of 490,635 (PLOD3^high^ vs. PLOD3^low^). NR, not reached. * indicates adjusted *P*-values < 0.05, ** indicates adjusted *P*-values < 0.01, *** indicates adjusted *P*-values < 0.001. Aggregate data in **a**, **d** and **f** are represented as means ± SD. Source data are provided as a Source Data file.

Baseline tumor samples from 34 patients were available for tertiary lymphoid structure (TLS) assessment. Patients with more TLS (mean area ≥30,000 μm^2^) had significantly higher ORR than those with less TLS (mean area <30,000 μm^2^) (71.4% vs. 25.0%) (Fig. 3a–c). However, there was no significant correlation between TLS and PFS. Additionally, tumor samples from 36 patients were available for proteomic analysis using a label-free technique. Among them, proteomic results were unassessed in three patients due to insufficient tissue. We totally identified 6,149 proteins (Fig. 3d, e; Supplementary Fig. 2; Supplementary Data 1). Patients with lower expression levels of promyelocytic leukemia (PML) or procollagen-lysine,2-oxoglutarate 5-dioxygenase 3 (PLOD3) in tumor showed significantly higher ORR (Fig. 3d, f) and longer PFS (PML: 4.9 vs. 13.9 months, HR = 5.53, 95% CI 1.6–19.7; PLOD3: 4.4 vs. 10.3 months, HR = 4.78, 95% CI 1.8–12.6, Fig. 3e, g) compared with those with higher expression of PML or PLOD3 protein.

**Fig. 4:**
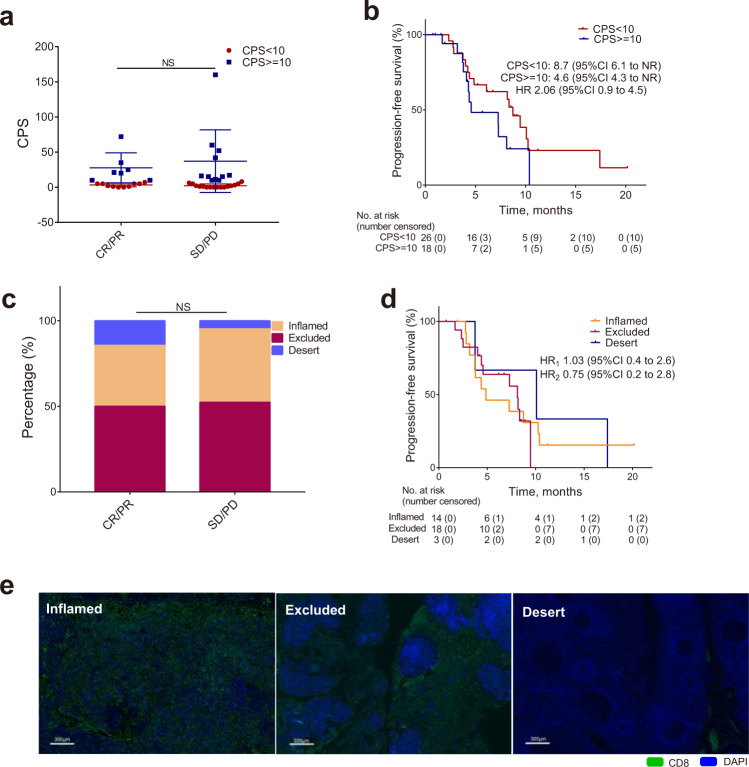
Biomarker analysis of CPS and immunophenotypes of tumor. **a** Association between response and CPS at cutoff value of 10. Each dot represents one patient, patients with SD/PD (*n* = 27) and with CR/PR (*n* = 17) biologically independent samples. Two-tailed chi-square test was used to determine statistical significance between the two groups. **b** Kaplan–Meier estimates of PFS by CPS (≥10 vs. <10). **c** Association between response and immunophenotypes of tumor. Two-tailed chi-square test was used to determine statistical significance between the groups. **d** Kaplan–Meier estimates of PFS by immunophenotypes of tumor (immune-inflamed vs. immune-excluded vs. immune-desert). NR not reached, NS not statistically significant, HR_1_ HR value of immune-excluded vs. immune-inflamed, HR_2_ HR value of immune-desert vs. immune-inflamed. **e** Representative images of different immunophenotypes, identified by CD8 (green) and DAPI. Scale bars, 300 μm. CD8 staining was performed one time in 35 independent samples with similar results, including patients with SD/PD (*n* = 21) and with CR/PR (*n* = 14). Aggregate data in **a** is represented as means ± SD. Source data are provided as a Source Data file.

Additionally, tumor samples were available from 32 patients for stromal TILs assessment, 44 patients for PD-L1 assessment, and 35 patients for immunophenotypes assessment at baseline. Correlations between proportion of TILs (>10%^15,20^) and ORR or PFS were not statistically significant (Supplementary Fig. 1a, b). We have examined the associations between PD-L1 status and efficacy as well. CPS (≥1 vs. <1, ≥10 vs. <10) did not correlate with ORR or PFS (8.1 vs. 6.1 months, HR = 0.64, 95% CI 0.3–1.6; 4.6 vs. 8.7 months, HR = 2.06, 95% CI 0.9–4.5, respectively) (Fig. 4a, b). As previous studies suggested that immunophenotypes of tumor may predict response of immunotherapy combined with chemotherapy in TNBC^21,22^, we explored the correlation between immunophenotypes of tumor and efficacy. There was no correlation between immunophenotypes and ORR or PFS (4.9 vs. 8.2 vs. 10.1 months, HR _excluded vs. inflammed_ = 1.03, 95% CI 0.4–2.6; HR_desert vs. inflammed_ = 0.75, 95% CI 0.2–2.8) (Fig. 4c–e).

To identify potential blood biomarkers, we assessed the proportions of immune cell subpopulations for 37 patients and performed the Olink proteomic assay for 40 patients (Supplementary Data 2). There was no correlation between the outcome and the proportions of CD4^+^ T cells, CD8^+^ T cells, NK cells, Tregs, or B cells in blood. Olink proteomic assay defined no correlations between plasma secretory factor levels and efficacy. Moreover, we divided TRAEs into 10 classes based on the involved systems, including general, nervous system, skin and subcutaneous tissue, blood and lymphatic system, gastrointestinal, urinary, cardiovascular, hemorrhagic, respiratory, and other. Patients with lower levels of CASP-8, IL-18, EGF, or ARG1 at baseline were more likely to develop urinary or general AEs, while those with higher levels of CXCL5 three weeks after treatment tended to develop more skin and subcutaneous tissue adverse events (Supplementary Table 6; Supplementary Fig. 3).

## Discussion

In this multicenter phase 2 study, our results revealed that combined therapy of camrelizumab, apatinib, and eribulin in patients with heavily pretreated advanced TNBC resulted in a ORR of 37.0%, a DCR of 87.0%, and a median PFS of 8.1 months along with a manageable toxicity profile. Moreover, this trial demonstrated that even patients with PD-L1-negative tumors or those underwent multiple lines of unsuccessful systemic therapies including checkpoint inhibitors could still benefit from this combination regimen.

Previous phase 3 studies had reported that checkpoint blockade in combination with chemotherapy could prolong survivals for patients with advanced PD-L1-positive TNBC in the first-line setting^3,4^. However, there are limited treatment options for patients with advanced TNBC who progressed after first-line chemotherapy or immunotherapy. Traditional chemotherapies presented unsatisfying antitumor activities as well as short PFS in these patients^9,10,12^. And checkpoint inhibitors alone or in combination with chemotherapy showed restricted clinical benefits as later-line therapy in patients with metastatic TNBC^5,6^. The KEYNOTE-119 study found that pembrolizumab as a single agent exhibited longer duration of response than chemotherapy in responding patients. However, the ORR (26.3%) and median PFS (3.4 months) remained unsatisfactory even in patients with a CPS ≥ 20, thus supporting the importance of combination therapy to produce improved disease control^6^. Acknowledging the weakness of cross-trial comparisons, the triplet regimen in this study displayed a more promising efficacy than other previously reported combinations. Patients with pretreated advanced TNBC in our study achieved an ORR of 37.0% and a DCR of 87.0%, which were higher than the ORR (21.8%) and DCR (50.5%) for patients treated with pembrolizumab plus eribulin as second/third-line therapy in ENHANCE 1 trial^5^. The median PFS of 8.1 months here was also longer than 4.1 months in ENHANCE 1 trial, 3.7 months with camrelizumab plus apatinib in our previous phase 2 trial^15^, and 6.0 months with high dose of apatinib plus vinorelbine in a prior phase 2 study^23^. In addition, eight patients (17.4%) had progressed after PD-1 inhibitors combined with chemotherapy in the previous treatment for advanced disease in the current trial. Two of these eight patients achieved PR, and five had SD. This finding suggests that PD-1 blockade in combination with apatinib and eribulin might have anti-tumoral activity for patients who are resistant to prior checkpoint inhibitors, which merits further investigation.

Camrelizumab plus apatinib and eribulin in our study also provided clinical benefits for patients with PD-L1-negative tumors or immune-desert phenotypes (based on the location of CD8^+^ T cells^24^). We and others found that tumor vascular normalization induced by antiangiogenic drugs could sensitize PD-1/PD-L1 blockade in multiple mouse models including breast carcinomas. The vessel normalization process could increase the PD-L1 expressions on tumor, endothelial and immune cells and reprogram tumor immune microenvironment (more TILs infiltration), which resulted in a “cold” tumor turning into an “inflamed” tumor in status^14,25,26^. Eribulin has been reported to induce tumor vasculature remodeling and normalization in mouse models^17^. In our previous phase II trial, the addition of apatinib to camrelizumab produced a higher ORR as compared to the pembrolizumab alone in the KEYNOTE-119 study (17.7%), regardless of PD-L1 status. However, the median PFS (3.7 months) remained suboptimal^6,15^. Further, the triplet regimen containing eribulin improved PFS irrespective of patients’ PD-L1 status (median: 8.1 months) while maintaining an ORR of 37% in the present study. Therefore, the immune-sensitizing effect of this triplet regimen in patients with PD-L1-negative tumors seemed to require the presence of both apatinib and eribulin together. However, this result should be interpreted with caution due to the limited statistical power of exploratory subgroup analysis from a small sample size.

The triplet treatment in this study showed no life-threatening or fatal adverse events, and the safety profile was manageable. Neutropenia was similar to eribulin monotherapy in terms of incidence and severity (Supplementary Table 7). However, we observed higher incidences of grade 3–4 ALT (17%), AST (17%), and bilirubin elevation (6.5%) than those reported in prior studies of camrelizumab in combination with apatinib or eribulin (Supplementary Table 7)^5,15,27^. Since eribulin-induced hepatotoxicity is minimal, the hepatotoxicity observed in this study may mainly be caused by the combination of camrelizumab and apatinib, which has also exhibited moderate liver toxicity in other study^27^. Another possible explanation for liver toxicity is the higher proportion of enrolled patients with liver metastasis compared with previous studies using camrelizumab plus apatinib (45.7 vs. 20.0~26.7%)^15,27^. Of note, the bilirubin elevation caused by triplet treatment was manageable, most of the patients (66.7%) recovered within one week, and only a few (6.5%) developed a grade 3 or 4 TRAE. Elevated aspartate aminotransferase, elevated alanine transaminase, and fatigue was the most common immunotherapy-related adverse events, consistent with previous reports of camrelizumab and other anti-PD-1 antibodies^28,29^. Rash and hand-foot syndrome were the most frequently adverse events related to apatinib dose reduction, which was similar to our prior phase 2 trial of camrelizumab plus apatinib^15^. Intriguingly, some biomarkers in peripheral blood potentiated to be predictive of the TRAE occurrence following the combined therapy in this study. Lower blood levels of CASP-8, IL-18, EGF, or ARG1 at baseline and increased CXCL5 in blood after treatment were associated with specific subcategories of TRAEs. Further large-scale studies are needed to endorse these findings.

Regarding the predictive biomarkers for responses to the triplet treatment, we demonstrated that patients with more tertiary lymphoid structure (TLS) in tumor had higher ORR. This result is consistent with recent studies that identified TLS as potential predictor for response to checkpoint inhibitors monotherapy in other solid tumors including malignant melanoma and soft-tissue sarcomas^30–32^. Furthermore, analysis of biopsies for potential proteomic predictive biomarkers of response showed that patients with lower levels of PML or PLOD3 in tumor had more favorable clinical outcomes. PML protein is known as a classic pro-apoptotic and growth-suppressive tumor suppressor. Previous studies found that high expression of PML in tumor was associated with poor prognosis of patients with metastatic breast cancer^33^, as well as lower intratumoral immune cells infiltration^34^. PLOD3 protein is a multifunctional enzyme with lysyl hydroxylase, collagen galactosyltransferase, and glucosyltransferase activities. It was reported to enhance tumor metastasis in lung cancer^35^, and more interestingly, high expression of PLOD3 was significantly correlated with resistance to PD-1 blockade therapy in colorectal cancer^36^. Therefore, our findings suggest that a subset of TNBC patients with more TLS or lower expression of PML and PLOD3 in tumor will be the group who may benefit more from camrelizumab combined with apatinib and eribulin.

The limitations of the study include single-arm design with no control group, and underpowered subgroup analysis due to small study population. Nonetheless, this trial demonstrates that camrelizumab combined with apatinib and eribulin has favorable efficacy with a manageable safety profile in patients with heavily pretreated advanced TNBC, even in those with PD-L1-negative or those who progressed after several lines of treatment including checkpoint inhibitors. Future randomized controlled trials are warranted to confirm our findings.

## Methods

Study protocol was approved by the Research Ethics Board of Sun Yat-sen Memorial Hospital, the First Affiliated Hospital of Sun Yat-sen University, and Changhai Hospital of Shanghai. This study was conducted in accordance with the Declaration of Helsinki. All patients provided written informed consent before enrollment.

### Study design and patients

In this prospective single-arm, multicentered, phase II clinical trial (ClinicalTrials.gov identifier: NCT04303741), patients with locally advanced or metastatic TNBC from three academic hospitals were enrolled. The exact dates of first and last patient enrollment were March 27, 2020 and May 27, 2021, respectively. Eligible patients include women age of 18–70 years with unresectable recurrent or metastatic TNBC defined by the American Society of Clinical Oncology/College of American Pathologists^37,38^; with measurable disease according to the Response Evaluation Criteria In Solid Tumors (RECIST) version 1.1; progressed after prior anthracycline and taxane, with at least one line of unsuccessful systemic therapy in the advanced setting; an Eastern Cooperative Oncology Group (ECOG) status of 0 or 1; and retained adequate organ and bone marrow function. Key exclusion criteria included clinically symptomatic central nerve system metastasis; and history of anti-CTLA-4, TIM3, LAG3, or T cell co-stimulation therapy (prior use of anti-PD-1/PD-L1 antibody was permitted); history of anti-angiogenic drugs or eribulin; and history of autoimmune disease.

### Procedures

Enrolled patients received combination therapy with intravenous camrelizumab 200 mg (3 mg/kg for patients with weight less than 50 kg) on day 1, apatinib 250 mg orally once daily, and intravenous eribulin 1.4 mg/m^2^ on days 1 and 8 of a 21-day cycle until progression, unacceptable toxicity, patient withdrawal, or death.

Imaging evaluation was done every 6 weeks for the first 24 weeks, and every 12 weeks thereafter. Efficacy was evaluated according to RECIST 1.1. Partial or complete response needed to be confirmed 4 weeks later.

Adverse events (AEs) were graded according to the National Cancer Institute Common Terminology Criteria for Adverse Events, version 4.03. Prespecified dose modifications of apatinib or eribulin were permitted, while dose adjustment of camrelizumab was not allowed. If use of any study drug was delayed for more than 4 weeks (8 weeks for camrelizumab) due to treatment-related AEs (TRAEs), the drug was discontinued.

### Biomarker analyses

Baseline tumor biopsy from metastatic or recurrent lesions was required, and the tumor biopsy should be taken no more than 6 months before enrollment. Peripheral blood samples were collected at baseline and three weeks after treatment.

#### PD-L1, TILs, CD8 and tertiary lymphoid structure (TLS) staining

PD-L1 expression of the tumor samples was measured using the FDA-cleared 22C3 assay on the Dako Link 48 platform (DAKO, clone number 22C3, 1:50 dilution,). PD-L1 expression was reported as combined positive score (CPS), defined as the number of PD-L1-positive cells (tumor cells, lymphocytes, and macrophages) divided by the total number of tumor cells multiplied by 100. Stromal tumor-infiltrating lymphocytes (TILs) were evaluated in hematoxylin and eosin sections following criteria proposed by the International Immuno-Oncology Biomarker Working Group^39^. Staining of CD8, PD-L1 and TLS were performed on FFPE sections. For immunophenotypes analysis, CD8 was stained with immunofluorescence using primary rabbit anti-human CD8 antibody (Thermo Fisher, catalog number MA5-14548, 1:200 dilution). TLS was stained by multiplex immunohistochemistry. Antibodies used for TLS analysis were primary rabbit anti-human CD4 antibody, (Abcam, catalog number ab133616, 1:500 dilution), primary rabbit anti-human CD8 antibody, (Abcam, catalog number ab93278, 1:4000 dilution), and primary rabbit anti-human CD20 antibody, (Abcam, catalog number ab78237, 1:50 dilution). CD20, CD8, and CD4 enriched area were defined as TLS.

#### FFPE proteomic analysis

Five to 10 FFPE slides (10 µm) were used for simultaneous isolation of protein. The FFPE samples were dewaxing and rehydration with three times of xylene and one time of 100%, 95%, 75%, 50% ethanol. Every sample was thoroughly mixed with 200 μL lysis buffer (4% SDS, 1% Protease inhibitor cocktail [Sigma]) and put on ice for 15 min. Then the samples were ultrasonicated for 10 min (3s-on, 3s-off). The lysis samples were heated at 95 °C, 750 rpm for 1 h. The concentration was measured by BCA method. The samples were added with 2 μL 500 mM DTT and kept at 56 °C for 1 h. Then 20 μL 500 mM IAM was added and the samples were kept at room temperature blocked of light for 45 min. Up to 60 ug protein was purified with SP3 beads. And 20 μL digestion buffer (50 mM NH_4_CO_3_, 50 mM CaCl_2_, 2.4 µg trypsin) was used to resuspend the beads. The proteins were digested overnight at 37 °C and 1 ug trypsin was further added to digest for another 3 h at 37 °C. The peptides were then purified by SP3 beads and eluted with 20 μL 2% iRT standards (Biognosys, Schlieren, Switzerland). The data from lysed peptide samples was acquired through Orbitrap Q Exactive HF mass spectrometry (Thermo Fisher Scientific, Waltham, MA) accompanied with a Thermo Scientific UltiMate 3000 UHPLC system. The digested peptides were ionized under 2 kiloVolts and introduced into mass spectrometry under a data-independent acquisition (DIA) mode. Ions with m/z ranging from 300 to 1,500 were acquired by Orbitrap mass analyzer at a high resolution of 60,000. Precursor ions were fragmented with higher energy collision dissociation (HCD) with normalized collision energy of 32%.

The data was searched against the human UniProt database (20,365 sequences) using Spectronaut software (version 14.5.200813.47784). The library generation with data applied the default settings with trypsin/P digest rule, high protein, peptide confidential level, and FDR of 0.01. Fold change of 1.5 times and *t*-test *P*-value of 0.05 were set as the cut off value for differential proteins. Proteins with area under curve values greater than 0.8 were analyzed for associations with clinical outcomes. Unsupervised clustering, heatmap were constructed using custom R scripts and R packages.

#### Blood flow cytometry and serum secreted proteomics

Immune cell subpopulations included CD4^+^ T cells (CD3^+^CD4^+^), CD8^+^ T cells (CD3^+^CD8^+^), NK cells (CD3^-^CD16^+^CD56^+^), Tregs (CD4^+^CD25^+^CD127^−^), and B cells (CD3^−^CD19^+^). For T cell subsets, natural killer (NK) cell and B cell detection, 50 μl peripheral blood was stained with BD Multitest 6-color TBNK Reagent (BD Biosciences), all were in 1:2 dilution, details were as follow: PC7-conjugated anti-CD4 (clone SK3), APC-conjugated anti-CD8 (clone SK1) or with FITC-conjugated anti-CD3 (clone SK7), PE-conjugated anti-CD16 (clone B73.1)/anti-CD56 (clone NCAM16.2) and PC7-conjugated anti-CD45 (clone 2D1) for 30 min at 4 °C, respectively. For Tregs detection, whole blood was stained with FITC conjugated anti-CD4 (Beckman, clone 13B8.2, 1:2 dilution), PC5-conjugated anti-CD25 (Beckman, clone B1.49.9, 1:2 dilution) and PE-conjugated anti-CD127 (Beckman, clone R34.34, 1:2 dilution) for 45 min at 4 °C. After antibody staining, hemolysin was used to lyse red blood cell (RBC). Single cell suspension was washed and then resuspended with 200 μl staining buffer for flow cytometry. Additionally, for serum secreted proteomics analysis, proximity extension analysis technology (Olink Bioscience AB) and associations between cytokines/chemokines, outcomes and AEs were performed by Welch Two Sample *t*-test (Supplementary Table 1). The fold change of plasma protein levels between groups more than one time was considered clinically significant.

### Outcomes

The primary endpoint was ORR per RECIST 1.1, defined as the proportion of patients with best response of complete or partial response. Secondary endpoints included incidence of TRAEs, disease control rate (DCR, proportion of patients with complete response [CR], partial response [PR] or stable disease [SD]), clinical benefit rate (CBR, proportion of patients with CR, PR or durable [≥ 24 weeks] SD), duration of response (DoR, time from the first documented CR or PR to disease progression or any-cause death), time to response (TTR, time from the initiation of study treatment to the first documented CR or PR), PFS (time from the initiation of study treatment to disease progression or any-cause death), 1-year OS rate (proportion of patients alive at 1 year), and potential biomarkers.

### Statistical analysis

Simon’s two-stage design was used^40^. The null hypothesis of ORR was 26% based on previously reported data of second- or later-line eribulin chemotherapy in patients with advanced TNBC from a randomized controlled trial^10^. The alternative hypothesis of ORR was 46%. According to the two-tailed test of 0.05 and the power of 0.80, 14 patients needed to be enrolled in the first stage. If 5 or more patients reached ORR in stage I, another 32 patients would be included in stage II. If more than 16 responders are observed in 46 patients, it has clinical significance.

We performed efficacy assessment both in the intention-to-treat (ITT) (patients who received at least one cycle of study treatment) and efficacy-evaluable population (patients had at least one post-treatment evaluation). We used the Clopper-Pearson method to calculate estimates of ORR, DCR, and CBR and the corresponding 95% confidence intervals (CIs). The median duration of PFS, DoR, 1-year OS rate, TTR and their 95% CIs were estimated by the Kaplan–Meier method. Additionally, we conducted subgroup analyses of associations between ORR and distinct factors by chi-square test or Fisher’s exact test. Subgroup factors included number of metastatic sites (≥3 vs. <3), liver metastasis (yes vs. no), prior treatment lines at advanced setting (>2 vs. 1–2), history of checkpoint inhibitors (yes vs. no) and disease-free interval defined as the interval from completion of chemotherapy to record of metastasis or recurrence (≥12 months vs. <12 months vs. de novo) and CPS (≥1 vs. <1; *≥*10 vs. <10).

Statistical analysis was performed using STATA 12.0 (Stata Co., College Station, TX), R studio (version 4.1.2) and Kaluza Analysis (version 2.0). All statistical tests were two-tailed, and *P* < 0.05 considered as statistically significant. Plots were constructed using R studio (version 4.1.2) and GraphPad Prism 7.0.

### Reporting summary

Further information on research design is available in the Nature Research Reporting Summary linked to this article.

## Supplementary information

Supplementary Information

Reporting Summary

Description of Additional Supplementary Files

Supplementary Data 1

Supplementary Data 2

## Data Availability

The study protocol is available as Supplementary Note 1 in the Supplementary Information file. The proteomics data generated in this study have been deposited in the iProX database^41^ under ID IPX0004386001 (PXD033655). Clinical data are not publicly available due to involving patient privacy, but can be accessed on request from the corresponding author Erwei Song for 10 years; individual de-identified participant data will be shared. The remaining data are available within the Article, Supplementary Information or Source Data file. Source data are provided with this paper.

## Source data

Source Data
