# Supplementary material for: Multicenter phase II trial of Camrelizumab combined with Apatinib and Eribulin in heavily pretreated patients with advanced triple-negative breast cancer
Source: Nat Commun. 2022 May 31;13:3011. doi: 10.1038/s41467-022-30569-0 (PMC9156739; doi:10.1038/s41467-022-30569-0)
Supplement: Supplementary file 1 — Supplementary Information [file 41467_2022_30569_MOESM1_ESM.pdf]

Contents

Supplementary Table 1. The Olink Immuno-Oncology panel including 92 cytokines/chemokines, growth factors, or soluble checkpoint-related proteins........2

Supplementary Table 2. Information of patients with prior anti-PD-1 antibody........5

Supplementary Table 3. Subgroup analysis........6

Supplementary Table 4. Clinical management and outcome of patients with elevated bilirubin........8

Supplementary Table 5. Reasons for drug dosing adjustments........9

Supplementary Table 6. Results of plasma analysis using Olink proteomic assay* 11

Supplementary Table 7. Toxicities among different treatment regimens........12

Supplementary Figure 1. Associations between outcomes and the stromal tumor-infiltrating lymphocytes (TILs). 13

Supplementary Figure 2. Results of FFPE proteomic. 14

Supplementary Figure 3. Plasma analysis using Olink proteomic assay. 15

Supplementary Figure 4. Example of FACS sequential gating strategies. 16

Supplementary Note 1. Study Protocol

Supplementary Table 1. The Olink Immuno-Oncology panel including 92 cytokines/chemokines, growth factors, or soluble checkpoint-related proteins

**Target**	**UniProt No**
Adenosine deaminase (ADA)	P00813
Adhesion G-protein coupled receptor G1 (ADGRG1)	Q9Y653
Angiopoietin-1 (ANGPT1)	Q15389
Angiopoietin-1 receptor (TIE2)	Q02763
Angiopoietin-2 (ANGPT2)	O15123
Arginase-1 (ARG1)	P05089
Carbonic anhydrase 9 (CAIX)	Q16790
Caspase-8 (CASP-8)	Q14790
C-C motif chemokine 13 (MCP-4)	Q99616
C-C motif chemokine 17 (CCL17)	Q92583
C-C motif chemokine 19 (CCL19)	Q99731
C-C motif chemokine 2 (MCP-1)	P13500
C-C motif chemokine 20 (CCL20)	P78556
C-C motif chemokine 23 (CCL23)	P55773
C-C motif chemokine 3 (CCL3)	P10147
C-C motif chemokine 4 (CCL4)	P13236
C-C motif chemokine 7 (MCP-3)	P80098
C-C motif chemokine 8 (MCP-2)	P80075
CD27 antigen (CD27)	P26842
CD40 ligand (CD40-L)	P29965
CD40L receptor (CD40)	P25942
CD70 antigen (CD70)	P32970
CD83 antigen (CD83)	Q01151
C-X-C motif chemokine 1 (CXCL1)	P09341
C-X-C motif chemokine 10 (CXCL10)	P02778
C-X-C motif chemokine 11 (CXCL11)	O14625
C-X-C motif chemokine 13 (CXCL13)	O43927
C-X-C motif chemokine 5 (CXCL5)	P42830
C-X-C motif chemokine 9 (CXCL9)	Q07325
Cytotoxic and regulatory T-cell molecule (CRTAM)	O95727
Decorin (DCN)	P07585
Fibroblast growth factor 2 (FGF2)	P09038
Fractalkine (CX3CL1)	P78423
Galectin-1 (Gal-1)	P09382
Galectin-9 (Gal-9)	O00182
Granzyme A (GZMA)	P12544
Granzyme B (GZMB)	P10144
Granzyme H (GZMH)	P20718
Heme oxygenase 1 (HO-1)	P09601

Hepatocyte growth factor (HGF)	P14210
ICOS ligand (ICOSLG)	O75144
Interferon gamma (IFN-γ)	P01579
Interleukin-1 alpha (IL-1 α)	P01583
Interleukin-10 (IL10)	P22301
Interleukin-12 (IL12)	P29459, P29460
Interleukin-12 receptor subunit beta-1 (IL12RB1)	P42701
Interleukin-13 (IL13)	P35225
Interleukin-15 (IL15)	P40933
Interleukin-18 (IL18)	Q14116
Interleukin-2 (IL2)	P60568
Interleukin-33 (IL33)	O95760
Interleukin-4 (IL4)	P05112
Interleukin-5 (IL5)	P05113
Interleukin-6 (IL6)	P05231
Interleukin-7 (IL7)	P13232
Interleukin-8 (IL8)	P10145
Killer cell immunoglobulin-like receptor 3DL1 (KIR3DL1)	P43629
Latency-associated peptide transforming growth factor beta-1 (LAP TGF-β-1)	P01137
Lymphocyte activation gene 3 protein (LAG3)	P18627
Lysosome-associated membrane glycoprotein 3 (LAMP3)	Q9UQV4
Macrophage colony-stimulating factor 1 (CSF-1)	P09603
Macrophage metalloproteinase-12 (MMP12)	P39900
Matrix metalloproteinase-7 (MMP7)	P09237
MHC class I polypeptide-related sequence A/B (MIC-A/B)	Q29983, Q29980
Mucin-16 (MUC-16)	Q8WXI7
Natural cytotoxicity triggering receptor (NCR1)	O76036
Natural killer cell receptor 2B4 (CD244)	Q9BZW8
Natural killer cells antigen CD94 (KLRD1)	Q13241
Nitric oxide synthase, endothelial (NOS3)	P29474
Placenta growth factor (PGF)	P49763
Platelet-derived growth factor subunit B (PDGF subunit B)	P01127
Pleiotrophin (PTN)	P21246
Pro-epidermal growth factor (EGF)	P01133
Programmed cell death 1 ligand 1 (PD-L1)	Q9NZQ7
Programmed cell death 1 ligand 2 (PD-L2)	Q9BQ51
Programmed cell death protein 1 (PDCD1)	Q15116
Stromal cell-derived factor 1 (CXCL12)	P48061
T-cell surface glycoprotein CD4 (CD4)	P01730
T-cell surface glycoprotein CD5 (CD5)	P06127
T-cell surface glycoprotein CD8 alpha chain (CD8A)	P01732
T-cell-specific surface glycoprotein CD28 (CD28)	P10747

TNF-related apoptosis-inducing ligand (TRAIL)	P50591
Tumor necrosis factor (TNF)	P01375
Tumor necrosis factor ligand superfamily member 12 (TWEAK)	O43508
Tumor necrosis factor ligand superfamily member 14 (TNFSF14)	O43557
Tumor necrosis factor ligand superfamily member 6 (FASLG)	P48023
Tumor necrosis factor receptor superfamily member 12A (TNFRSF12A)	Q9NP84
Tumor necrosis factor receptor superfamily member 21 (TNFRSF21)	O75509
Tumor necrosis factor receptor superfamily member 4 (TNFRSF4)	P43489
Tumor necrosis factor receptor superfamily member 9 (TNFRSF9)	Q07011
Vascular endothelial growth factor A (VEGFA)	P15692
Vascular endothelial growth factor receptor 2 (VEGFR2)	P35968

(Olink Bioscience AB)

The panel and experiment details can be found on the manufacturer's website: https://www.olink.com/resourcessupport/document-download-center/.

Supplementary Table 2. Information of patients with prior anti-PD-1 antibody

Patient	Prior used combinations	Number of prior cycles of immunotherapy	Best overall response of prior anti-PD-1 treatment	Best overall response in this study	Progression-free survival (months)	Time to response (months)
# 01	Anti-PD-1 antibody + Paclitaxel	2	Progressive disease	Partial response	17.4	4.2
# 02	Anti-PD-1 antibody + Paclitaxel	2	Progressive disease	Stable disease	2.8	-
# 03	Anti-PD-1 antibody + Gemcitabine+ Capecitabine	13	Stable disease	Partial response	7.3	1.5
# 04	Anti-PD-1 antibody + Gemcitabine + Paclitaxel	5	Stable disease	Stable disease	4.9	-
# 05	Anti-PD-1 antibody + Paclitaxel	6	Stable disease	Stable disease	4.0	-
# 06	Anti-PD-1 antibody + Paclitaxel	9	Stable disease	Progressive disease	4.2	-
# 07	Anti-PD-1 antibody	4	Stable disease	Stable disease	1.6	-
# 08	Anti-PD-1 antibody + Gemcitabine	3	Progressive disease	Stable disease	3.6	-

Supplementary Table 3. Subgroup analysis

	N (%)	Objective response rate (%)	*P* value of objective response rate*	Progression-free survival (months)	Hazard ratio (95% CI)	*P* value of progression-free survival*
**Number of metastatic sites**						
<3	23 (50.0)	52.2	0.067	8.7	1.00 (reference)	0.039
≥3	23 (50.0)	21.7		5.8	2.27 (1.0-5.0)	
**Liver metastasis**						
No	25 (54.3)	40.0	0.873	8.3	1.00 (reference)	0.518
Yes	21 (45.7)	33.3		6.1	1.28 (0.6-2.7)	
**Prior therapy in the advanced setting**						
1-2	17 (37.0)	35.3	>0.999	7.3	1.00 (reference)	0.837
>2	29 (63.0)	37.9		8.1	1.10 (0.5-2.6)	
**Disease-free interval**						
≥12 months	13 (28.3)	38.5	0.927	8.1	1.00 (reference)	
<12 months	20 (43.5)	40.0		7.3	1.29 (0.6-3.0)	0.549
De novo	13 (28.3)	30.8		7.3	0.87 (0.3-2.6)	0.810
**CPS≥1**						
No	8 (17.4)	25.0	0.635	6.1	1.00 (reference)	0.348
Yes	36 (78.3)	41.7		8.1	0.64 (0.3-1.6)	
Unknown#	2 (4.3)					
**CPS≥10**						

No	26 (56.5)	38.5	>0.999	8.7	1.00 (reference)	0.071
Yes	18 (39.1)	38.9		4.6	2.06 (0.9-4.5)	
Unknown[#]	2 (4.3)					

[*] Two-tailed t-test and Kaplan-Meier estimates of PFS were used to determine statistical significance between groups.

[#] The two patients with unknown CPS score were the patients who discontinued study treatment before the first scheduled post-baseline assessment.

Supplementary Table 4. Clinical management and outcome of patients with elevated bilirubin

	Grades of elevated bilirubin[a]	Clinical treatment	Recovery within one week	Recovery within two weeks
#1	2	● Ursodeoxycholic acid	√	
#2	3	● Hold immunotherapy, and eribulin<br>● Apatinb dose reduction<br>● Prednisone 1 mg/kg/day<br>● Ursodeoxycholic acid	√	
#3	1	● Ursodeoxycholic acid	√	
#4	2	● Ursodeoxycholic acid	√	
#5	3	● Hold immunotherapy, and eribulin<br>● Apatinb dose reduction<br>● Prednisone 1 mg/kg/day<br>● Ursodeoxycholic acid		√
#6	3	● Hold immunotherapy, and eribulin<br>● Apatinb dose reduction<br>● Prednisone 1 mg/kg/day<br>● Ursodeoxycholic acid		√

[a]Adverse events were graded according to the National Cancer Institute Common Terminology Criteria for Adverse Events, version 4.03.

Supplementary Table 5. Reasons for drug dosing adjustments

**Reasons for dose interruption of camrelizumab**				
	**Reasons for first dose interruption (*n*=7)**		**Reasons for second dose interruption (*n*=1)**	
	Grade 2	Grade 3	Grade 3	
# 01		Elevated bilirubin and leukopenia	Elevated bilirubin and leukopenia	
# 02	Proteinuria			
# 03		Leukopenia, thrombocytopenia and elevated alanine transaminase or aspartate transaminase		
# 04		Elevated bilirubin and leukopenia		
# 05		Leukopenia, thrombocytopenia and pneumonia		
# 06		Elevated bilirubin		
# 07		Leukopenia, thrombocytopenia, and elevated alanine transaminase or aspartate transaminase		
**Reasons for apatinib dose reduction**				
	**Reasons for first dose reduction[a] (*n*=13)**		**Reasons for second dose reduction[b] (*n*=4)**	
	Grade 2	Grade3	Grade 2	Grade3
# 01	Rash[c]	Elevated alanine transaminase or aspartate transaminase and elevated bilirubin	Rash[c]	
# 02	Rash[c]		Rash[c]	
# 03		Elevated alanine transaminase or aspartate transaminase and elevated bilirubin		Elevated alanine transaminase or aspartate transaminase and elevated bilirubin
# 04		Thrombocytopenia		
# 05[d]		Thrombocytopenia		
# 06		Hand-foot syndrome and thrombocytopenia		Hand-foot syndrome
# 07		Hand-foot syndrome		
# 08		Hand-foot syndrome		
# 09	Canker sore	Elevated alanine transaminase or aspartate transaminase and elevated bilirubin		
# 10		Hand-foot syndrome		
# 11		Rash[c]		
# 12	Rash[c]			
# 13	Rash[c]			
**Reasons for eribulin dose reduction**				

	Reasons for first dose reduction[e] (*n*=13)		Reasons for second dose reduction[f] (*n*=3)		Reason for eribulin discontinuation (*n*=3)
	Grade 3	Grade 4	Grade 3	Grade 4	
# 01	Elevated alanine transaminase or aspartate transaminase		Elevated alanine transaminase or aspartate transaminase		Elevated bilirubin
# 02	Thrombocytopenia				
# 03	Pain		Pain		Pain
# 04	Leukopenia				
# 05		Leukopenia and thrombocytopenia			
# 06	Leukopenia				
# 07	Leukopenia				
# 08	Leukopenia				
# 09	Thrombocytopenia				
# 10					Economic reason
# 11	Thrombocytopenia				
# 12		Leukopenia and thrombocytopenia	Leukopenia and thrombocytopenia		
# 13	Elevated alanine transaminase or aspartate transaminase				

[a]The first drug reduction of apatinib was from 250 mg daily to 125 mg daily.

[b]The second dose was adjusted to 125 mg with one day on and one day off.

[c]Rash was caused by allergy to apatinib, the symptoms disappear in most patients after dose adjustment.

[d]Patient discontinued study treatment due to adverse event.

[e]The first dose reduction was adjusted from 1.4 mg/m^2 to 1.1 mg/m^2.

[f]The second dose reduction was adjusted from 1.1 mg/m^2 to 0.7 mg/m^2.

Supplementary Table 6. Results of plasma analysis using Olink proteomic assay*

	Cytokine/ chemokine	With treatment-related adverse events, mean	Without treatment-related adverse events, mean	*P* value#
General (baseline samples)		n=14	n=26	
	CASP-8	4.82	8.24	0.0001
	IL-18	9.81	12.49	<0.0001
	EGF	5.04	7.68	0.002
	ARG	4.35	6.77	0.0004
Urinary (baseline samples)		n=8	n=32	
	CASP-8	4.35	7.72	0.003
	IL-18	9.59	12.04	0.006
	EGF	4.47	7.33	0.005
	ARG	3.92	6.43	0.007
Skin and subcutaneous tissue (post-treatment samples)		n=23	n=6	
	CXCL5	9.56	6.40	0.0004

*The numbers inside the table represent NPX value.

#Two-tailed Welch's t-test was used to determine statistical significance between the two groups.

Supplementary Table 7. Toxicities among different treatment regimens

Toxicities (%)	Eribulin + camrelizumab + apatinib	Camrelizumab + apatinib [1]	Eribulin (Study 301) [2]	Eribulin (EMBRACE) [3]
Neutropenia				
Grade 1-2	21.9	NA (<2%)	8.5	7
Grade 3-4	30.4	0	45.7	45
AST elevation				
Grade 1-2	55.5	70	0	NA (<10%)
Grade 3-4	17.4	10	3.3	NA (<10%)
ALT elevation				
Grade 1-2	47.9	63.3	NA (<10%)	NA (<10%)
Grade 3-4	17.4	0	NA (<2%)	NA (<10%)
Bilirubin elevation				
Grade 1-2	6.5	20	NA (<10%)	NA (<10%)
Grade 3-4	6.5	0	NA (<2%)	NA (<10%)

[1] Liu, *et al*. J Immunother Cancer. 2020;8(1):e000696.

[2] Kaufman, *et al*. J Clin Oncol. 2015;33(6):594-601.

[3] Cortes, *et al*. Lancet. 2011;377(9769):914-23.

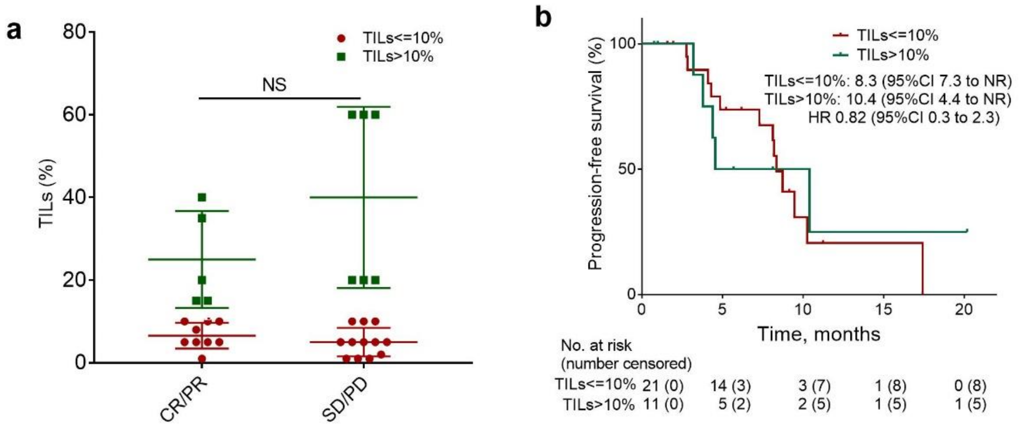

Supplementary Figure 1. Associations between outcomes and the stromal tumor-infiltrating lymphocytes (TILs). (a) Associations between response and the stromal TILs level (>10% *vs.* ≤10%). Two-tailed t-test was used to determine statistical significance between the two groups. Each dot represents one patient (*P*>0.999), patients with SD/PD (n=18) and with CR/PR (n=14) biologically independent samples. Aggregate data are represented as means ±SD. (b) Kaplan-Meier estimates of progression-free survival by stromal TILs level (>10% *vs.* ≤10%). NR, not reached.

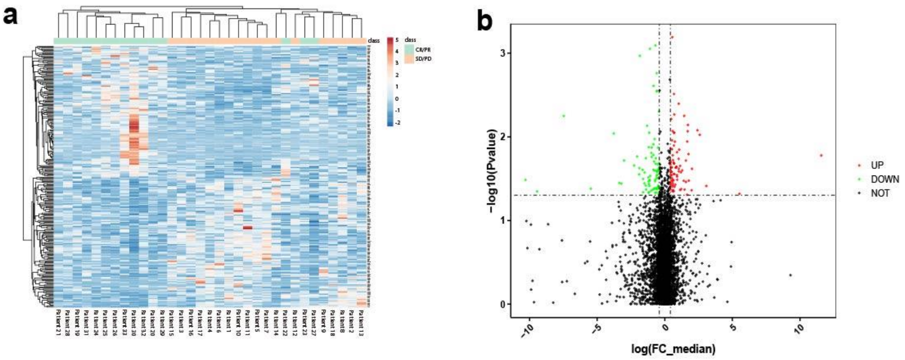

Supplementary Figure 2. Results of FFPE proteomic. (a) Heatmap of differentially expressed proteins in 33 patients. The color of each cell represents average protein abundance. (b) Volcano plot of protein expression levels comparing patients with SD/PD (n=18) to those with CR/PR (n=15). y axis, −log10 (*P* value) and x axis, log2 (fold change). Two-sided t-test was used to determine statistical significance between the two groups.

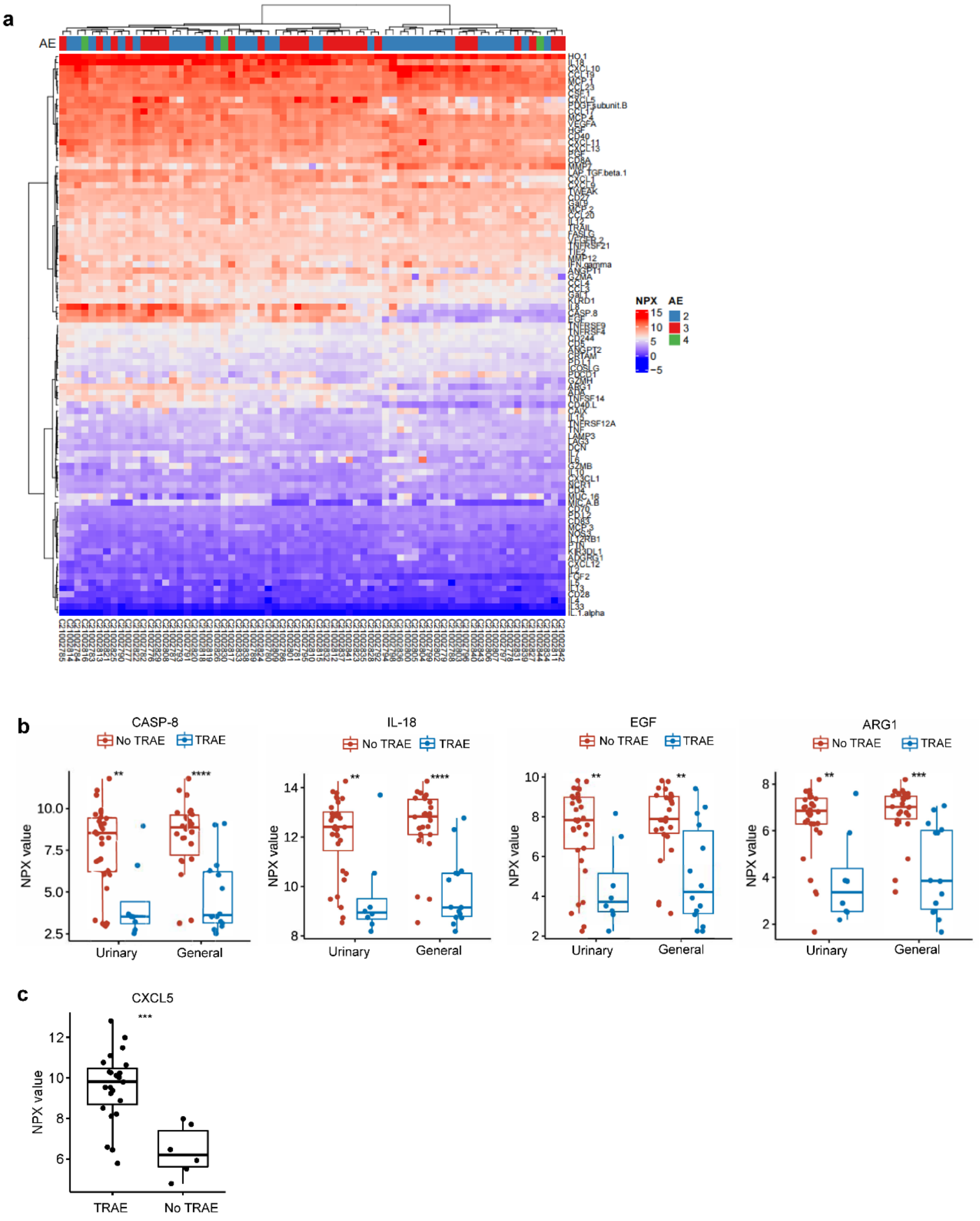

Supplementary Figure 3. Plasma analysis using Olink proteomic assay. (a) Heatmap of plasma protein of all samples. Each column represents a different sample, n=37 biologically independent samples. Rows indicate NPX value. One NPX difference means a doubling of protein concentration. Positive values (red) indicate higher expression and negative values (blue) indicate lower expression. (b) Baseline plasma of CASP-8, IL-18, EGF or ARG level in patients with and without urinary or general treatment related adverse events (TRAEs). Each dot represents one patient, patients with SD/PD (n=27) and with CR/PR (n=13) biologically independent samples. (c) Post-treatment plasma of CXCL5 level in patients with and without skin and subcutaneous tissue TRAEs. Each dot represents one patient, patients with SD/PD (n=16) and with CR/PR (n=13) biologically independent samples. In (b) and (c), a two-tailed Welch's t-test was used to determine statistical significance between the two groups. ** P<0.01; *** P<0.001; **** P<0.0001. Box plots in (b) and (c) are represented as minima, maxima, center, bounds of box and whiskers and percentile. P-value are available in Supplementary Table 6.

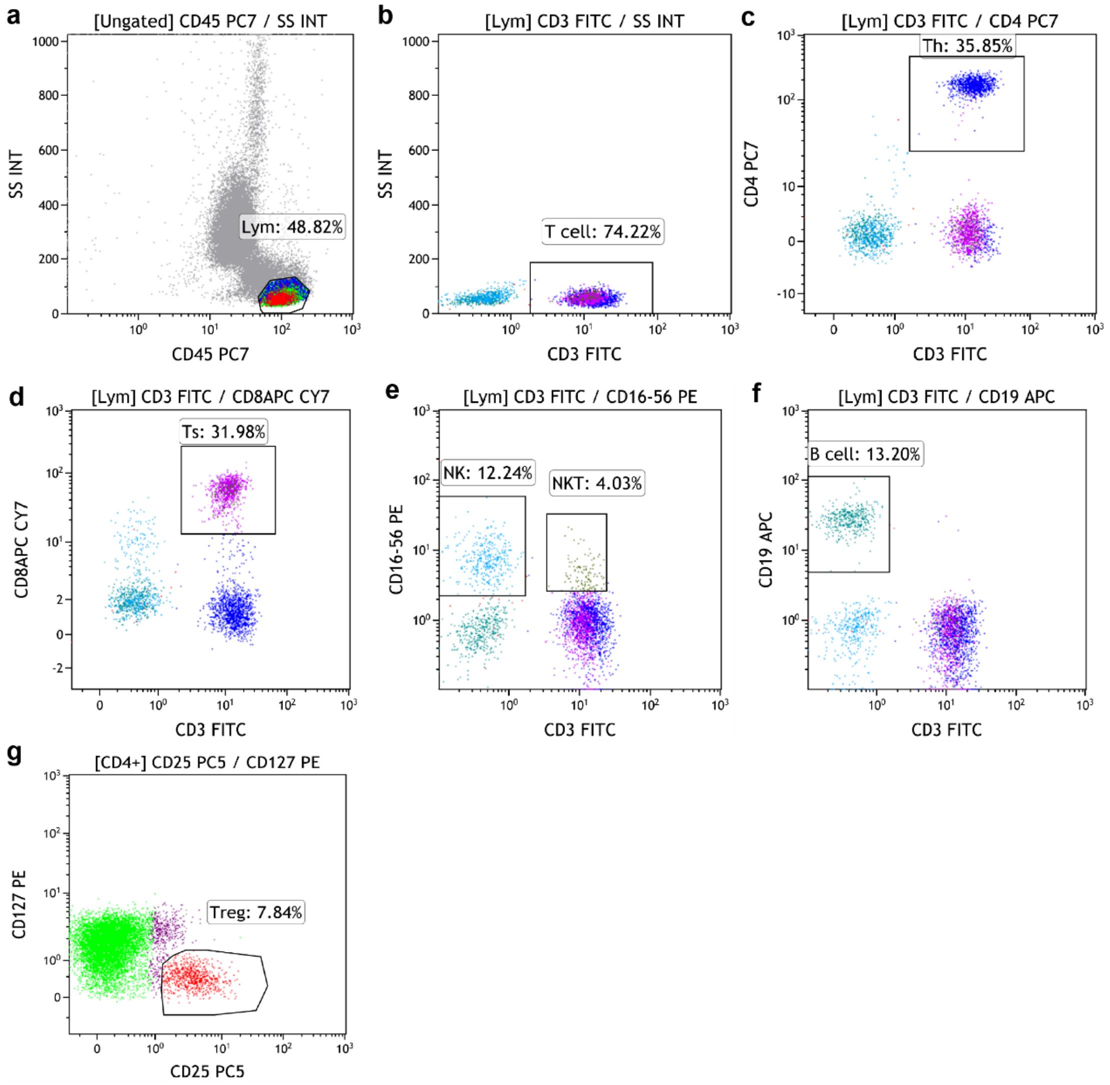

Supplementary Figure 4. Example of FACS sequential gating strategies. (a) Gating strategy of lymphocyte ($CD45^+$ cells). (b) Gating strategy of T cells ($CD3^+$ cells). (c) Gating strategy of $CD4^+$ T cells ($CD3^+CD4^+$ cells). (d) Gating strategy of $CD8^+$ T cells ($CD3^+CD8^+$ cells). (e) Gating strategy of NK cells ($CD3^-CD16^+CD56^+$ cells). -: en-dash. (f) Gating strategy of B cells ($CD3^-CD19^+$ cells). (g) Gating strategy of Treg cells ($CD4^+CD25^+CD127^-$ cells).

A Trial of Camrelizumab in Combination with Apatinib and Eribulin in Patients with Advanced TNBC

Study Protocol

Study Protocol No.:	Immuno2020-01
Version Number of the Study Protocol:	1.0
Version Date of the Study Protocol:	Jan. 29th, 2020
Study Director:	Dr. Erwei Song
Study Center:	Sun Yat-sen Memorial Hospital of Sun Yat-sen University
	First Affiliated Hospital of Sun Yat-sen University
	Changhai Hospital of Shanghai

Protocol Signature Page

Signature of Principal Investigator

I will carefully execute the duties as an investigator in accordance with the Chinese GCP, and personally participate in or directly lead this clinical study. We have read and confirmed this protocol (protocol number: Immuno2020-01, version number: 1.0, version date: Jan.29th, 2020). I agree to execute relevant duties in accordance with the laws of China, the Declaration of Helsinki, Chinese GCP and this clinical study protocol. In addition, I confirm that any measures are subject to approval by the Ethics Committee before implementation, unless they must be taken to protect the safety, rights and interests of subjects.

Study Center: Sun Yat-sen Memorial Hospital of Sun Yat-sen University

Erwei Song	Erwei Song	29/01/2020
Principal Investigator (print)	Principal Investigator (signature)	Signature Date (DD/MM/YYYY)

Table of Contents

Protocol Summary....6
1 Background....21
2 Study Objectives and Endpoints....23
2.1 Study Objectives....23
2.2 Endpoints....23
3 Study Design....24
3.1 Overall Design....24
3.2 Blinding....24
4 Subject Selection and Withdrawal/Discontinuation....24
4.1 Inclusion Criteria....24
4.2 Exclusion Criteria....25
4.3 Subject Withdrawal....26
4.4 Termination Criteria....27
5 Study Drugs....27
5.1 Overview of the Study Drugs....27
5.2 Formulation of Camrelizumab....28
5.3 Formulation of Eribulin....28
5.4 Dosing Regimen....29
5.5 Dose Modification....29
5.6 Drug Management, Dispensation and Return....32
5.7 Combined Medication....33
6 Study Procedures....35
6.1 Screening Period (D-21–D-1):....35
6.2 Treatment Period....38
6.3 Follow-Up Period....39
7 Safety Evaluation....45
7.1 Adverse Event (AE)....45
7.2 Serious Adverse Event (SAE)....47
7.3 Pregnancy....50

7.4 AE of Special Interest....50
7.5 Infusion Reactions....51
7.6 Immune-Related Adverse Events (irAEs)....51
7.7 Symptomatic Treatment for Apatinib-Related Adverse Reactions....54
7.8 Symptomatic Treatment for Erublin-Related Adverse Reactions....58
8 Efficacy Evaluation....60
9 Study Management....60
9.1 Ethics and Informed Consent....60
9.2 Protocol Amendments....61
9.3 Quality Assurance of the Clinical Trial....61
9.4 Data Management....62
10 Data Processing and Intellectual Property....62
10.1 Data Processing....62
11 Statistical Analysis....63
11.1 Determination of Sample Size....63
11.2 Analysis Sets....63
11.3 Analysis of the Primary Endpoint....64
11.4 Analysis of the Secondary Endpoints....64
11.5 General Analysis....64
11.6 Baseline Characteristics....65
11.7 Subgroup Analysis....65
11.8 Safety Evaluation....65
11.9 Other Analyses....67
12 End of Study and Expected Schedule....68
12.1 End of Study....68
12.2 Expected Study Schedule....68
13 Reference....68
Appendix 1. Prohibited Traditional Chinese Medicine (TCM)....70
Appendix 2. Performance Status Criteria (ECOG)....71
Appendix 3. Calculation of Creatinine Clearance....72
Appendix 4. Management Principles for irAEs....73

Appendix 5. Response Evaluation Criteria in Solid Tumors 78

Appendix 6. Percent Bone Marrow in Human Skeleton 94

Protocol Summary

Study Title	A Trial of Camrelizumab in Combination with Apatinib and Eribulin in Patients With Advanced TNBC
Study Protocol No.	Immuno2020-01
Version No.	1.0
Version Date	Jan. 29th, 2020
Sponsor	Sun Yat-sen Memorial Hospital of Sun Yat-sen University
Study Center	Sun Yat-sen Memorial Hospital of Sun Yat-sen University;<br>First Affiliated Hospital of Sun Yat-sen University;<br>Changhai Hospital of Shanghai
Principal Investigator	Dr. Erwei Song
Study Objectives	**Study Objectives:**<br>To observe and evaluate the efficacy and safety of camrelizumab in combination with apatinib and eribulin in patients with advanced triple-negative breast cancer
Endpoints	**Primary Endpoint:**<br>Objective response rate (ORR)<br>**Secondary Endpoints:**<br>1. Incidence and severity of adverse events (AE) and serious adverse events (SAE)<br>2. Disease control rate (DCR): The proportion of subjects who achieved CR/PR/SD<br>3. Clinical benefit rate (CBR): The proportion of subjects with CR, PR, and SD for more than 6 months (including) during the study<br>4. Duration of response (DoR)<br>5. Time to response (TTR)<br>6. Progression-free survival (PFS) based on RECIST 1.1<br>7. One-year overall survival (OS) rate<br>8. Exploratory analysis of the relationship between molecular markers and efficacy
Sample Size	14–46 subjects
Study Design	**A single-arm, multi-center, open-label, phase II clinical study**<br>200 mg camrelizumab (if the weight of patient is less than 50 kg, the dose of camrelizumab is 3 mg/kg), *iv*, d1, q3W; plus 250 mg apatinib, *po* daily, continuously given until the occurrence of an event meeting the discontinuation criteria; plus 1.4mg/m^2 eribulin *iv*, d1, d8, q3w
Dosing Regimen	Camrelizumab is administered via intravenous infusion (without prophylactics) at a dose of 200 mg (if the weight of patient is less than 50kg, the dose of camrelizumab is 3mg/kg) within 30 min (not less than 20 min, no more than 60 min), once every 3 weeks. Each cycle contains 3 weeks. Camrelizumab is administered until the occurrence of an event meeting the discontinuation criteria;<br>Apatinib is taken orally after meals, once a day, and continued throughout the study;

	Eribulin is administered via intravenous infusion at a dose of 1.4mg/m^2 within 2-5 min, on the d1 and d8 every 3 weeks. Each cycle contains 3 weeks. Eribulin is administered until the occurrence of an event meeting the discontinuation criteria.
Sample Size Estimation	According to SIMON's two-stage design, if ≥ 5 subjects of the 14 subjects experience tumor response during the treatment, a total of 32 patients will be enrolled in the second stage, so that the total number of subjects is about 46.
Inclusion Criteria	1. Subjects must participate voluntarily and sign the informed consent form;<br>2. Females aged 18-70 years old;<br>3. Pathologically confirmed advanced triple-negative breast cancer [ER-negative (IHC ER-positive percentage < 1%), PR-negative (IHC PR-positive percentage < 1%), HER2-negative (IHC-/+ or IHC++ but FISH/CISH-)] with at least one measurable lesion according to RECIST v1.1 criteria;<br>4. Previously failed the treatment with anthracycline and taxane; the number of chemotherapy lines was ≥ 1 in the advanced stage; Subjects who relapsed within 6 months after the end of neoadjuvant chemotherapy or adjuvant chemotherapy were considered first-line treatment failure;<br>5. Ability to swallow tablets;<br>6. ECOG PS score: 0–1;<br>7. Expected survival ≥ 12 weeks;<br>8. The major organ functions meet the following requirements (not including any use of blood components and cell growth factors during the screening period):<br>● Absolute neutrophil count ≥ 1.5 × 10^9/L;<br>● Platelets ≥ 100 × 10^9/L;<br>● Hemoglobin ≥ 9 g/dL;<br>● Serum albumin ≥ 3 g/dL;<br>● Thyroid stimulating hormone (TSH) ≤ ULN (In case of abnormalities, T3 and T4 levels should be investigated at the same time. If T3 and T4 levels are normal, the subject can be enrolled);<br>● Bilirubin ≤ ULN (In case of Gilbert's syndrome, or liver metastasis, bilirubin ≤1.5 × ULN);<br>● ALT and AST ≤ 1.5 × ULN (In case of hepatic metastases, ALT and AST ≤ 3 × ULN);<br>● AKP ≤ 2.5 × ULN;<br>● Serum creatinine ≤ 1.5 × ULN or creatinine clearance ≥ 60 mL/min (standard Cockcroft-Gault formula, as shown in Appendix III) within 7 days of initial administration ;<br>9. For female patients of childbearing potential or female patients who are not sterilized by surgical operations, they need to use a medically approved contraceptive measure (such as an intrauterine device, contraceptives or condom) during the study treatment period and within 24 weeks after the end of the study treatment; For female patients of childbearing potential who are not sterilized by

	surgical operations, they must have a negative serum or urine HCG test result within 7 days prior to study enrollment.
Exclusion Criteria	1. Subjects with clinically symptomatic CNS metastasis (e.g., cerebral edema requiring hormonal intervention, or progression of brain metastasis). For patients who have received treatment for brain or meningeal metastasis, they can be enrolled if they are clinically stable (MRI) for at least 1 month;<br>2. Subjects who have received any other study drugs within 28 days prior to the first dose of this study;<br>3. Subjects who have experienced severe hypersensitivity reactions to other monoclonal antibodies;<br>4. Patients who have previously received radiotherapy, chemotherapy, hormone therapy, surgery or molecular targeted therapy in less than 28 days after the end of such treatments (last dose) and before the dosing of the study drug;<br>5. Patients with hypertension which cannot be well controlled by antihypertensive drugs (systolic pressure ≥ 140 mmHg or diastolic pressure ≥ 90 mmHg);<br>6. Subjects who have previously received treatment with anti-CTLA-4, TIM3, LAG3 and other antibodies or T cell co-stimulation therapy (previous use of anti-PD-1 or PD-L1 antibody was permitted);<br>7. Subjects who have previously received treatment with anti-angiogenic drugs or eribulin;<br>8. Subjects with any active autoimmune diseases or a history of autoimmune diseases (including but not limited to the following: autoimmune hepatitis, interstitial pneumonia, uveitis, enteritis, hepatitis, hypophysitis, vasculitis, nephritis, hyperthyroidism, decreased thyroid function; adult subjects with vitiligo or completely relieved childhood asthma can be enrolled if they do not require any intervention; subjects with asthma requiring medical intervention with bronchodilators cannot be enrolled);<br>9. Subjects with clinical symptoms or diseases of the heart that are not well controlled, such as: (1) > NYHA grade 2 cardiac failure (2) unstable angina (3) myocardial infarction within 1 year (4) clinically significant supraventricular or ventricular arrhythmia requiring treatment or intervention;<br>10. The routine urinalysis indicates that urine protein is ≥ ++ or confirms that 24 h urine protein is ≥1.0 g;<br>11. Patients with known hereditary or acquired hemorrhage and thrombophilia (such as hemophilia, coagulopathy, thrombocytopenia, hypersplenism, etc.);<br>12. Subjects with congenital or acquired immunodeficiency (such as HIV infection);<br>13. Subjects who have been inoculated with live vaccines within less than 4 weeks before the first dose or are likely to be inoculated during the study;<br>14. Subjects who are allergic to or contraindicated to the test drug.
Discontinuation Criteria for Subject	Subjects will withdraw from/terminate the treatment when any one of the following conditions occur:<br>1. Subjects withdraw informed consent and request to withdraw from the study;

	2. Radiological examination shows disease progression (The patients who are judged by investigators to have either potential pseudo progression, or are clinically benefiting, can continue the treatment until they are confirmed to be disease progression by a second radiologic scan 4 weeks later);<br>3. Subjects showing unacceptable toxicity;<br>4. Subjects with poor compliance;<br>5. Subjects lost to follow-up or show positive blood HCG results;<br>6. Subjects who have received camrelizumab for up to two years (without radiological examination showed disease progression);<br>7. Subjects are required to withdraw for other investigator-assessed causes.
Termination Criteria of the Study	The termination criteria of this study include but are not limited to the following:<br>1. Discovery of unexpected, significant or unacceptable risks to the subjects;<br>2. Major errors in the protocol are discovered during the implementation of the trial;<br>3. The study drug/trial treatment is ineffective, or the continued implementation of the trial is meaningless.
Safety Evaluation	The severity of adverse events is determined according to the CTCAE v4.0.3 criteria. The adverse event record form should be filled out honestly during the trial, including the occurrence time, severity, duration, actions taken and outcomes of the adverse events.
Efficacy Evaluation	The enrolled subjects undergo imaging evaluation once every 2 cycles (6 weeks). At 6 months after the first dose, a clinical tumor imaging evaluation is performed every 12 weeks ± 7 days as appropriate. Tumor efficacy is evaluated according to RECIST v1.1 criteria, and the subjects first evaluated as PR/CR must be verified 4 weeks later. The subjects who were judged by investigators to have potential pseudo progression must be confirmed by a second radiologic scan 4 weeks later.
Statistical Methods	Trial results are mainly analyzed using descriptive statistics. Measurement data will be summarized using the mean, standard deviation, median, maximum, minimum. Enumeration data and ranked data will be summarized using the frequency (proportion), percentage, and confidence interval.<br>Statistical analysis will be performed using the STATA (version 13.0; Stata Co., College Station, TX).<br>Safety Analysis:<br>Descriptive statistical analysis is primarily used to analyze the adverse events, serious adverse events and adverse reactions (the adverse reactions are defined as adverse events that are "definitely related, possibly related, and not assessable" to the study drug). For laboratory results, the cases in which the pre-treatment values are normal but the post-treatment values are abnormal will be described.<br>Efficacy Analysis:<br>The point estimates of efficacy endpoints such as objective response rate (ORR), disease control rate (DCR), and clinical benefit rate (CBR) with the corresponding two-sided 95% confidence intervals (CI) were calculated using the Clopper-Pearson method. For survival, the median duration of progression-free survival, one-year overall survival

	rate, and their 95% confidence intervals representing the entire population are estimated using the Kaplan-Meier method.
End of Study	The study is completed (the expected first time of data lock) when all enrolled patients reached the primary study endpoint and the last case was enrolled 6 months after the start of treatment.
Study Duration	Expected Scheduled Duration: Feb. 2020 to Dec. 2022

Schedule of Activities

<table>
<tr><th rowspan="3">Item</th><th colspan="2">Screening Period</th><th></th><th colspan="3">Treatment Period (21days/cycle)</th><th colspan="2" rowspan="2">Post-Treatment</th><th rowspan="2">Survival Follow-Up</th></tr>
<tr><th rowspan="2">Within 3 Weeks Before Dosing</th><th rowspan="2">Within 1 Week Before Dosing</th><th colspan="2">Cycle 1</th><th colspan="2">Cycles 2–26</th></tr>
<tr><th>Day 1</th><th>Day 8 (±3d)</th><th>Day 1 (±3d)</th><th>Day 8 (±3d)</th><th>End of Treatment/ Withdrawal</th><th>Visit after End of Treatment</th><th>Every 2 Months (±3d)</th></tr>
<tr><td colspan="10">Baseline Data</td></tr>
<tr><td>Signing Informed Consent</td><td>×</td><td></td><td></td><td></td><td></td><td></td><td></td><td></td><td></td></tr>
<tr><td>Demographic Data</td><td>×</td><td></td><td></td><td></td><td></td><td></td><td></td><td></td><td></td></tr>
<tr><td>Tumor History / Other Medical History[1]</td><td>×</td><td colspan="8"></td></tr>
<tr><td>Concomitant Medication[2]</td><td>×</td><td>×</td><td colspan="6">×</td><td></td></tr>
<tr><td colspan="10">Laboratory Tests</td></tr>
<tr><td>Blood Routine [3]</td><td></td><td>×</td><td></td><td>×</td><td>×</td><td>×</td><td>×</td><td>×</td><td></td></tr>
<tr><td>Urinalysis [4]</td><td></td><td>×</td><td></td><td>×</td><td>×</td><td>×</td><td>×</td><td>×</td><td></td></tr>
<tr><td>Routine Stool Test [5]</td><td></td><td>×</td><td></td><td>×</td><td>×</td><td></td><td>×</td><td></td><td></td></tr>
<tr><td>Blood Biochemistry [6]</td><td></td><td>×</td><td></td><td>×</td><td>×</td><td>×</td><td>×</td><td>×</td><td></td></tr>
<tr><td>Coagulation Function Test [7]</td><td></td><td>×</td><td></td><td>×</td><td>×</td><td></td><td>×</td><td></td><td></td></tr>
</table>

**Item**	**Screening Period**			**Treatment Period (21days/cycle)**			**Post-Treatment**		**Survival Follow-Up**
	Within 3 Weeks Before Dosing	Within 1 Week Before Dosing	**Cycle 1**		**Cycles 2–26**				
			Day 1	Day 8 (±3d)	Day 1 (±3d)	Day 8 (±3d)	End of Treatment/ Withdrawal	Visit after End of Treatment	Every 2 Months (±3d)
T3, FT3, FT4 and TSH [8]	×				×		×		
Pituitary Adrenal Axis Test [9]	×								
Myocardial Zymogram [10]		×					×		
Hepatitis B, Hepatitis C and HIV Test [11]	×								
Pregnancy Test [12]		×					×		
Clinical Evaluation and Examination									
Adverse Events [13]	**From signing the informed consent form until 30 days after the last dose**								
Vital Signs [14]		×	×	×	×	×	×	×	
Physical Examination [15]		×	×	×	×	×	×	×	
ECOG PS Score		×			×		×	×	
Electrocardiogram [16]		×		×	×	×	×		
Echocardiography [17]		×					×		
Blood Pressure Monitoring [18]	×	×		×	×	×	×		
Study Drugs									

Item	Screening Period			Treatment Period (21days/cycle)			Post-Treatment		Survival Follow-Up
	Within 3 Weeks Before Dosing	Within 1 Week Before Dosing	Cycle 1		Cycles 2–26				
			Day 1	Day 8 (±3d)	Day 1 (±3d)	Day 8 (±3d)	End of Treatment/ Withdrawal	Visit after End of Treatment	Every 2 Months (±3d)
Camrelizumab Administration [19]			×		×				
Apatinib Administration [20]			Orally administration once a day after meal						
Dispensation/Return of Apatinib [21]			×		×		×		
Eribulin Administration [22]			×	×	×	×			
Imaging Evaluation									
Imaging Examination [23]	×		Once every 2 cycles in the first 8 cycles of dosing, followed by once every 3 cycle				×		
Follow-Up after the Treatment									
Time to Progression [24]							Imaging evaluation is carried out once every 3 months (±7 d) until disease progression or start of new anti-tumor treatments (subjects with non-imaging PD)		
Time to Death [25]									×
Blood Sample Collection and Tumor Sample Collection/Harvest									
Collection/Harvest of Biomarkers [26]		×			×				

Notes:

[1] Tumor history/other medical histories: Pathological findings, ER/PR/HER2 detection report; a history of tumor surgery, chemotherapy, radiotherapy, and other disease treatments; tumor history other than breast cancer.

[2] Record the concomitant medication and concomitant treatment given within 30 days prior to the start of dosing and during the study period. Once a subject interrupts the trial treatment, only the concomitant medication and treatment used to solve new or unresolved adverse events related to the trial treatment should be recorded.

[3] Blood routine: Hemoglobin, red blood cell count, white blood cell count, neutrophil count, lymphocyte count, and platelet count are measured within 7 days before enrollment, on Day 8 of Cycle 1, Day 1 and Day 8 of subsequent cycles, at the end of study, and 30 days after the treatment.

[4] Urinalysis: Urine protein, urine glucose, urine occult blood (urine red blood cells, white blood cells). If the protein results of two consecutive semi-quantitative methods are 2+, a quantitative test of 24-h urine protein is performed. If the semi-quantitative method shows that the protein is > 2+, the quantitative test of 24-h urine protein is performed; carried out within 7 days before the enrollment, on Day 8 of Cycle 1, Day 1 and Day 8 of subsequent cycles, at the end of study, and 30 days after the treatment.

[5] Routine stool test: Occult blood; re-examination is needed for fecal occult blood+ and a gastroscopy examination may be needed based on the judgment of the investigator. Performed within 7 days before enrollment, on Day 8 of Cycle 1 and Day 1 of subsequent cycles, and at the end of study.

[6] Blood biochemistry: total bilirubin, conjugated bilirubin, ALT, AST, AKP, γ-GT, total protein, albumin, urea/blood urea nitrogen, creatinine, uric acid, fasting blood glucose, TG, cholesterol, potassium, sodium, chlorine, calcium, phosphorus, blood lipase (only during the screening period and in case of subsequent abdominal pain, abdominal distension and other symptoms of suspected pancreatitis), blood amylase (only during the screening period and subsequent abdominal pain, abdominal distension and other symptoms of suspected pancreatitis). It is carried out within 7 days before enrollment, on Day 8 of Cycle 1, Day 1 and Day 8 of subsequent cycles, at the end of study, and 30 days after the treatment.

[7] Coagulation function: INR, APTT, PT, TT, FIB; Performed within 7 days before enrollment, on Day 8 of Cycle 1 and Day 1 of subsequent cycles, and at the end of study.

[8] Thyroid function test: Including serum FT3, FT4, and TSH. Performed within 21 days before dosing, on Day 1 of Cycle 2, and then once every 3 cycles.

[9] Pituitary adrenal axis test: Including ACTH, cortisol, and sex hormone, which are performed during the screening period.

[10] Myocardial zymogram examination: One examination should be performed within 7 days before enrollment. Then, this examination is only supplemented upon the onset of precordial pain and palpitations, as well as abnormal electrocardiograms. It is also carried out at the end of treatment/withdrawal.

[11] Hepatitis B, hepatitis C and HIV tests: 5 items of hepatitis B examination. If the test results are abnormal, virus replication (HBV DNA) test should be performed; hepatitis C virus antibody (anti-HCV) and HIV antibody test.

[12] Pregnancy test: The urine pregnancy test is performed 72 h before the first dose in women of childbearing potential. If the result of the urine pregnancy test is positive, a serum pregnancy test is performed. If necessary, re-tests can be performed for confirmation.

[13] Adverse events: Adverse events (AEs) should be recorded from signing the informed consent until at least 30 days after the last dose and should be followed-up until the AEs are resolved or stabilized. SAE and irAE observed within 90 days after the last dose of camrelizumab should be followed up. If the subjects start a new anti-tumor treatment, they should be followed-up until they start the tumor treatment (if the tumor treatment is started within 30 days after the last dose, the subjects should be followed-up until at least 30 days after the last dose; if the tumor treatment is started more than 30 days after the last dose, the subjects should be followed-up until they start the new tumor treatment).

[14] Vital sign examination: Body temperature, respiration, heart rate, and blood pressure. Performed within 7 days before enrollment, on Day 1 and Day 8 of Cycle 1, Day 1 and Day 8 of subsequent cycles, at the end of study, and 30 days after treatment.

[15] Physical examination: Examination of main body systems (head and face, skin system, lymph nodes, eyes, ears, nose, throat, oral cavity, respiratory system, cardiovascular system, abdomen, genitourinary system, musculoskeletal system, nervous system, and mental state). Physical examination is performed within 7 days before enrollment, on Day 1 and Day 8 of Cycle 1, on Day 1 and Day 8 of subsequent cycles, at the end of study, and 30 days after treatment.

[16] 12-Lead ECG: Performed within 7 days before enrollment, on Day 8 of Cycle 1, Day 1 and Day 8 of subsequent cycles, and at the end of study. Abnormalities in ECG must be confirmed twice via additional ECG tests.

[17] Echocardiography: An echocardiography examination is performed within 7 days prior to the enrollment and at the end of the study, and additional echocardiography examinations are required for abnormalities with clinical significance during the study period.

[18] Blood pressure monitoring: The blood pressure measurement of subjects is performed by the investigator during the screening period; during each blood pressure measurement, smoking and coffee are prohibited for 30 min before the measurement, and subjects should at least rest for 10 min, and the sitting position is taken during the measurement by placing the elbow at the same level as the heart. Each blood pressure measurement is taken on the same side of the body; during the trial, the blood pressure monitoring is performed by the subjects themselves and recorded in their diary card. Blood pressure is detected at least 3 times a week in the first 2 cycles. If the blood pressure is abnormal, the measurement is carried out every day; if the blood pressure is normal, the blood pressure measurement is carried out twice a week after 2 cycles; in addition, the blood pressure is measured by the investigator during each follow-up. During each blood pressure

measurement, smoking and coffee are prohibited for 30 min before the measurement, and subjects should at least rest for 10 min, and the sitting position is taken during the measurement by placing the elbow at the same level as the heart. Each blood pressure measurement is taken on the same side of the body.

[19] Camrelizumab administration: Camrelizumab is administered via intravenous infusion (without prophylactics) at a fixed dose of 200 mg within 30 min (not less than 20 min, no more than 60 min), once every 3 weeks. Each cycle contains 3 weeks. Camrelizumab is administered until the occurrence of an event meeting the discontinuation criteria.

[20] Apatinib is taken orally after meals, once a day, continuously.

[21] Return and dispensation of apatinib. Apatinib is dispensed on Day 1 of Cycle 1. From Day 1 of Cycle 2, the return and dispensation of apatinib are done on Day 1 of each cycle. The remaining drugs are returned first to verify the dose actually taken before new study drugs are dispensed.

[22] Eribulin administration: Eribulin is administered via intravenous injection at a fixed dose of 1.4mg/m^2 within 2-5 min, on Day 1 and Day 8 of every 3 weeks. Each cycle contains 3 weeks. Eribulin is administered until the occurrence of an event meeting the discontinuation criteria.

[23] Imaging examination: including CT or MRI of the chest, abdomen, and brain. The baseline tumor evaluation during the screening period can be relaxed to 3 weeks before treatment. Qualified CT/MRI scan results obtained before signing the informed consent can be used for tumor evaluation during the screening period. Bone scan should be performed upon clinically suspected bone metastases. Absence of cerebral hemorrhage should be confirmed within 21 days before randomization for patients with stable brain metastases.

During the treatment period, the imaging examination should be performed under the same conditions as those of the baseline examination (layer thickness of the scan, use of contrast agent, etc.), and the lesions found at baseline should be checked every 2 cycles in the first 6 cycles of treatment (bone scan is done in case of suspected bone progress or for CR confirmation). After 6 cycles, imaging examination can be performed every 3 cycles and can be done as appropriate upon the discovery of suspected new lesions. The first PR/CR must be confirmed after 4 to 6 weeks; the first PD must be confirmed after 4 to 6 weeks (except for significant changes in subjects' symptoms or rapid tumor progression).

The window period for imaging examination schedule is ± 7 days. Unplanned imaging examinations can be performed when progressive disease (such as worsening of symptoms) is suspected.

[24] In addition to the progressive disease as evidenced by imaging, subjects who have ended the trial treatment for other reasons must be evaluated at the end of treatment if the imaging examination is not performed within 4 weeks prior to the end of the trial. At the same time, tumor efficacy is followed up every 3 months after the end of the trial until records confirm the progressive disease or initiation of new tumor treatments.

[25] Survival follow-up: After the trial treatment is discontinued, the survival status and subsequent anti-cancer treatment can be collected through clinical or telephone follow-ups every 2 months until death.

[26] Blood samples (20 mL) shall be collected from each subject at baseline and centrifuged to retain plasma, while the blood cells shall be cryopreserved below -70 °C. Subjects showing CR/PR during the trial must be subject to an additional biomarker blood sample collection upon the onset of CR/PR and PD, respectively, and the collection requirements are the same as those of the baseline period; the existing paraffin-embedded tumor samples or fresh biopsy specimens (section after fixation and paraffin-embedding) are collected to make ≥ 8 slices that are 5μm thick.

List of Abbreviation

Abbreviations and Terms	English Full Name
ADA	Anti-drug antibody
ADRs	Adverse drug reactions
AE	Adverse event
AKP	Alkaline phosphatase
ALT	Alanine aminotransferase
ANC	Absolute neutrophil count
APTT	Activeated partial thromboplastin time
AST	Aspartate aminotransferase
AUC	Area under curve
BP	Blood pressure
BUN	Blood urea nitrogen
Ca	Calcium
CFDA	China Food and Drug Administration
Cl	Chlorine
Cr	Creatinine
CR	Complete response
CRF	Case report form
CT	Computed tomography
CTCAE	Common Terminology Criteria for Adverse Events
DBIL	Direct bilirubin
DCR	Disease control rate
DLT	Dose limited toxicity
ECG	Electrocardiogram
ECOG	Eastern Cooperative Oncology Group
eCRF	Electronic Case report form
EDC	Electroinc data collection
FAS	Full analyse set
FIB	Fibrinogen
FDA	Food and Drug Administration
FT3	free triiodothyronine
FT4	free thyroxine
GCP	Good Clinical Practice
Glu	Glucose
Hb	Hemoglobin
irAE	Immune-related adverse event

Abbreviations and Terms	English Full Name
INR	International Normalized Ratio
IRB	Institutional review board
ITT	intend to treat
K	Potassium
LLN	lower limits of normal
LVEF	Left ventricular ejection fraction
mPFS	median progression-free survival
MRI	Magnetic Resonance Imaging
MTD	Maximum torlerate dose
Na	Sodium
NCCN	National Comprehensive Cancer Network
NCI-CTC	National cancer institute Common Terminology Criteria
ORR	Objective response rate
OS	Overall survival
P	Phosphorus
PD	Progressive disease
PD-1/PD-L1	Programmed death 1/programmed death ligand 1
PFS	Progression-Free-Survival
PK	Pharmacokinetics
PLT	Platelet
PPS	Per-Protocol Set
PR	Partial response
PT	Prothrombin time
QoL	Quality of life
RBC	Red blood cell
RO	Receptor occupancy
RECIST	Response evaluation criteria in solid tumors
SAE	Serious adverse event
SAS	Statistical Analysis System (software)
SD	Stable disease
SOP	Standard Operation Procedure
SS	Safety Analysis set
TB	Total bilirubin
TC	Total cholesterol
TCM	Traditional Chinese Medicine
TG	Triglyceride

Abbreviations and Terms	English Full Name
TP	Plasma total protein
TSH	Thyroid-stimulating hormone
TT	Thrombin time
UA	Blood uric acid
UICC	Union for International Cancer Control
ULN	Upper limit of normal
WBC	White blood cell
γ-GT	γ-Glutamyltransferase

1 Background

Breast cancer is a common malignant tumor endangering women's health, with the highest incidence and rising trend among Chinese women's cancers. Breast cancer is the sum of a group of diseases, at least divided into four molecular subtypes: luminal A, luminal B, HER2 overexpressing and, triple-negative type. Triple-negative breast cancer (TNBC) accounts for ~15% of the total cases of breast cancer, which is characterized by a high early recurrence rate, high distant metastasis rate, poor prognosis, and low survival rate[1 2]. Due to the lack of effective targeted drugs for advanced metastatic TNBC, chemotherapy is currently recommended by the international consensus, and the combination of albumin paclitaxel chemotherapy and PD-L1 antibody immunotherapy is recommended for patients with positive PD-L1 expression as the first-line treatment, but drug resistance often occurs during the treatment process[3]. Therefore, it has become a hot and difficult issue to find effective targeting drugs for relapsed and metastatic TNBC, especially for patients with front-line treatment progression or patients with negative PD-L1 expression.

In recent years, tumor immunotherapy has made breakthrough progress. However, the single-use of anti-PD-1/PD-L1 antibodies in many TNBC patients did not achieve good efficacy [4].Impassion130 trial found that first-line PD-L1 antibody combined with albumin paclitaxel significantly prolonged progression-free survival (PFS) and overall survival (OS) in PD-L1-positive advanced TNBC compared with albumin paclitaxel alone, but did not improve the prognosis of PD-L1-negative patients[5]. Therefore, it is urgent to find a backline treatment option for TNBC in patients with PD-L1 negative TNBC (approximately 60% of total TNBC patients), as well as after progression of first-line or multiline therapy. Several preclinical studies in recent years have shown that in mouse models of in-situ and recurrent breast cancer, anti-angiogenic drugs can significantly enhance the inhibition effect of anti-PD-1 or PD-L1 antibodies on tumor growth by inducing tumor vascular normalization and enhancing the anti-tumor immune effect of CTL in the tumor microenvironment [6,7]. A border in advanced liver cancer, gastric cancer/stomach esophagus carcinoma clinical studies (N = 43) found that camrelizumab (200 mg, q2w) + apatinib (125 mg/d, 250 mg/d, or 500 mg/d) gained a high ORR, and the adverse reactions were controllable in 250 mg group. One of the most common adverse events associated with level 3 or higher treatment was high blood pressure and AST increased (15.2%), with no treatment-related deaths[8]. In a clinical trial of patients with wild type EGFR and ALK gene compared with advanced squamous I / II non-small cell lung cancer (N = 96), using apatinib(250 mg/d, 375 mg/d, or 500 mg/d) with camrelizumab (200 mg, q2w) treatment, found that apatinib 250 mg group gained controllable adverse reactions and clinical efficacy [9]. Besides, our previous pre-

clinical study also found that low-dose VEGFR2 inhibitor can sensitized breast carcinoma to anti-PD-1 antibody by promoting tumor vascular normalization and re-programming tumor immune microenvironment [10]. Our previous phase II clinical trial (N=40) showed that camrelizumab combined with apatinib could significantly increase the ORR of advanced TNBC to 43%, and it was effective in patients with PD-L1$^-$ tumors or patients received 1-2 lines of chemotherapy. The adverse events were controlled, and the incidence of ≥3 grade therapy-related adverse events was 25%. However, the OS extension was not significant [11]. In conclusion, we speculated that to further improve the ORR and OS of advanced TNBC patients, it is necessary to combine chemotherapy with camrelizumab and apatinib. Eribulin is a low toxicity and effective chemotherapy drug that can block microtubule lengthening, and has the effect of vascular remodeling and reversing EMT. A global, multicenter, randomized phase III study has shown that eribulin prolongs OS and has less toxicity than other common chemotherapy drugs in the treatment of advanced breast cancer that has failed multi-line therapies [12]. Also, a previous study found that anti-PD-1 antibody combined with eribulin achieved the ORR to 26.4% in patients with metastatic TNBC [13]. Therefore, we proposed a single-arm, multi-center, open, researchers launched clinical research, aimed at exploiting the efficacy and tolerability of combinational treatment of anti-PD-1 antibody camrelizumab, VEGFR2 inhibitors apatinib and eribulin in patients with advanced TNBC, and exploring tumor tissue or hematological molecular markers that can predict the efficacy of the treatment. The results of this trial may improve the response rate and prolong the survival of patients with advanced TNBC after first-line or multiline therapies.

This clinical study involves a drug with the trade name of Camrelizumab®, the generic name of which is Camrelizumab for Injection. It is a recombinant humanized anti-PD-1 monoclonal antibody, which has been approved and marketed in China in May 2019. Previous research data showed that the recombinant humanized anti-PD-1 monoclonal antibody injection developed by Hengrui Pharmaceutical has considerable in vivo efficacy and safety compared with similar foreign drugs, and may have better anti-tumor clinical application potential.

The clinical study involved another drug, marketed as Apatinib®, generically known as Apatinib Mesylas or Apatinib Mesylas, which is a small molecule tyrosine kinase inhibitor. Apatinib was approved by the China Food and Drug Administration on November 17, 2014. Apatinib has also shown efficacy in phase II clinical trial of advanced triple-negative breast cancer by significantly prolonging the progression-free survival (PFS) of patients.

This clinical study also involves a drug with the trade name of Halaven®, the generic name of Eribulin Mesylate Injection, which is a microtubule inhibitor. The China Food and Drug

Administration approved eribulin for the treatment of advanced breast cancer in December 2019. The drug has been approved abroad to treat advanced breast cancer .

1.1 Drug Name and Physiochemical Properties

[Generic Name] Camrelizumab for Injection

[English Name] Camrelizumab for Injection

[Molecular Weight] About 143 KD

[Dosage Form] Lyophilized Powder for Injection

[Strength] 200 mg/vial

[Generic Name] Apatinib Mesylate Tablets

[English Name] Apatinib Mesylate Tablets

[Molecular Weight] About 493.58 KD

[Dosage Form] Tablet

[Strength] 250 mg/tablet

[Generic Name] Eribulin Mesilate Injection

[English Name] Eribulin Mesilate Injection

[Molecular Weight] About 826. 0 KD

[Dosage Form] Injection

[Strength] 2ml:1mg

2 Study Objectives and Endpoints

2.1 Study Objectives

To observe and evaluate the efficacy and safety of anti-PD-1 antibody camrelizumab in combination with apatinib mesylate and eribulin in patients with unresectable recurrent, or metastatic TNBC.

2.2 Endpoints

Primary Endpoint:

- Tumor objective response rate (ORR)

Secondary Endpoints:

- Incidence and severity of adverse events (AE)

- Clinical benefit rate (CBR): The proportion of subjects with CR, PR, and SD for more than 6 months (including) during the study
- Duration of response (DoR)
- Time to response (TTR)
- Progression-free survival (PFS) based on RECIST v1.1
- One-year overall survival rate
- Exploratory analysis of the relationship between molecular markers in tumor/peripheral blood and efficacy.

3 Study Design

3.1 Overall Design

A single-arm, multi-center, open-label, phase II clinical study

Camrelizumab 200mg (3mg/kg for patient whose weight is below 50kg) *iv* q3W combination with apatinib 250mg, *po*, daily (d1-d21) and eribulin1.4mg/m^2 *iv* d1, d8 q3W

3.2 Blinding

This study is an open-label study and does not involve blinding

4 Subject Selection and Withdrawal/Discontinuation

4.1 Inclusion Criteria

1. Subjects must participate voluntarily and sign the informed consent form;
2. Females aged 18-70 years old;
3. Pathologically confirmed advanced triple-negative breast cancer [ER-negative (IHC ER-positive percentage < 1%), PR-negative (IHC PR-positive percentage < 1%), HER2-negative (IHC-/+ or IHC++ but FISH/CISH-)] with at least one measurable lesion according to RECIST v1.1 criteria;
4. Previously failed the treatment with anthracycline and taxane; the number of chemotherapy lines was ≥ 1 in the advanced stage;
5. Ability to swallow tablets;
6. ECOG PS score: 0–1;
7. Expected survival ≥ 12 weeks;
8. The major organ functions meet the following requirements (not including any use of blood components and cell growth factors during the screening period):

- Absolute neutrophil count ≥ 1.5×10^9/L;
- Platelets ≥ 100×10^9/L;

- Hemoglobin ≥ 9 g/dL;
- Serum albumin ≥ 3 g/dL;
- Thyroid stimulating hormone (TSH) ≤ ULN (In case of abnormalities, T3 and T4 levels should be investigated at the same time. If T3 and T4 levels are normal, the subject can be enrolled);
- Bilirubin ≤ ULN (In case of Gilbert's syndrome, or liver metastasis, Bilirubin ≤ 1.5 × ULN);
- ALT and AST ≤ 1.5 × ULN (In case of hepatic metastases, ALT and AST ≤ 3 × ULN);
- AKP ≤ 2.5 × ULN;

9. For female patients of childbearing potential or female patients who are not sterilized by surgical operations, they need to use a medically approved contraceptive measure (such as an intrauterine device, contraceptives or condom) during the study treatment period and within 24 weeks after the end of the study treatment; For female patients of childbearing potential who are not sterilized by surgical operations, they must have a negative serum or urine HCG test result within 7 day prior to study enrollment.

4.2 Exclusion Criteria

1. Subjects with clinically symptomatic CNS metastasis (e.g., cerebral edema requiring hormonal intervention, or progression of brain metastasis). For patients who have received treatment for brain or meningeal metastasis, they can be enrolled if they are clinically stable (MRI) for at least 1 month;
2. Subjects who have received any other study drugs within 28 days prior to the first dose of this study;
3. Subjects who have experienced severe hypersensitivity reactions to other monoclonal antibodies;
4. Patients who have previously received radiotherapy, chemotherapy, hormone therapy, surgery or molecular targeted therapy in less than 28 days after the end of such treatments (last dose) and before the dosing of the study drug;
5. Patients with hypertension which cannot be well controlled by antihypertensive drugs (systolic pressure ≥ 140 mmHg or diastolic pressure ≥ 90 mmHg);
6. Subjects who have previously received treatment with anti-CTLA-4, TIM3, LAG3 and other antibodies or T cell co-stimulation therapy (Previous use of anti-PD-1 or PD -L1 antibody was permitted);
7. Subjects who have previously received treatment with anti-angiogenic drugs or eribulin;
8. Subjects with any active autoimmune diseases or a history of autoimmune diseases (including but not limited to the following: autoimmune hepatitis, interstitial pneumonia, uveitis, enteritis, hepatitis, hypophysitis, vasculitis, nephritis, hyperthyroidism, decreased thyroid function; adult subjects with vitiligo or completely relieved childhood asthma can be enrolled if they do not require

any intervention; subjects with asthma requiring medical intervention with bronchodilators cannot be enrolled);

9. Subjects with clinical symptoms or diseases of the heart that are not well controlled, such as: (1) > NYHA grade 2 cardiac failure (2) unstable angina (3) myocardial infarction within 1 year (4) clinically significant supraventricular or ventricular arrhythmia requiring treatment or intervention;
10. The routine urinalysis indicates that urine protein is ≥ ++ or confirms that 24 h urine protein is ≥ 1.0 g;
11. Patients with known hereditary or acquired hemorrhage and thrombophilia (such as hemophilia, coagulopathy, thrombocytopenia, hypersplenism, etc.);
12. Subjects with congenital or acquired immunodeficiency (such as HIV infection);
13. Subjects who have been inoculated with live vaccines within less than 4 weeks before the first dose or are likely to be inoculated during the study;
14. Subjects who are allergic to or contraindicated to the test drug.

4.3 Subject Withdrawal

4.3.1 Criteria for Subject Withdrawal/Treatment Discontinuation

Subjects will withdraw from/terminate the treatment when any one of the following conditions occur:

1. Subjects withdraw informed consent and requested to withdraw from the study;
2. Radiological examination showed disease progression (The patients who are judged by investigators to have either potential pseudo progression, or are clinically benefiting, can continue the treatment until they are confirmed to be disease progression by a second radiologic scan 4 weeks later).
3. Subjects showing unacceptable toxicity;
4. Subjects with poor compliance;
5. Subjects lost to follow-up or show positive blood HCG results;
6. Subjects who have received camrelizumab for up to two years (without radiological examination showed disease progression);
7. Subjects are required to withdraw for other investigator-assessed causes.

4.3.2 Handling of Withdrawn Subjects

The efficacy and safety investigations will be completed upon trial withdrawal as specified in the protocol must be completed as much as possible. In addition, the safety follow-up should be completed along with fully documented AEs and their outcomes. The survival follow-up should be completed to record subsequent treatment regimens and the survival status of the subjects. The investigator can recommend or provide a new or alternative treatment to the subject based on the condition of the

subject. Patients showing no progressive disease need to be continuously followed-up for imaging evaluation until the subjects begin a new treatment or show progressive disease.

4.4 Termination Criteria

The termination criteria of this study include but are not limited to the following:

1. Discovery of unexpected, significant or unacceptable risks to the subjects;
2. Major errors in the protocol are discovered during the implementation of the trial;
3. The study drug/trial treatment is ineffective, or the continued implementation of the trial is meaningless.

5 Study Drugs

5.1 Overview of the Study Drugs

- Camrelizumab:

Name: Camrelizumab for Injection

Manufacturer: Suzhou Shengdiya Biomedical Co., Ltd.

Dosage Form: Lyophilized powder for injection

Route of Administration: Intravenous infusion

Strength: 200 mg/20 mL vial.

Storage and stability: Placed in a 2-8 °C medical refrigerator during storage and the shelf life is tentatively set to 2 years. Do not freeze.

- Apatinib:

Name: Apatinib Mesylate Tablets (Apatinib)

Manufacturer: Jiangsu Hengrui Medicine Co., Ltd.

Dosage Form: Tablet

Route of Administration: Oral

Strength: 250 mg/tablet

Storage and Stability: Away from light, sealed, stored at below 25 °C. Valid for 2 years.

- Eribulin

Name: Eribulin Mesilate Injection (Halaven)

Manufacturer: Vetter Pharma-Fertigung GmbH & Co. KG

Dosage Form: Injection

Route of Administration: Intravenous injection

Strength: 2ml:1mg

Storage and Stability: Sealed and stored at below 25℃. Do not freeze. Store in the original package. Valid for 2 years.

5.2 Formulation of Camrelizumab

The camrelizumab investigational drug is a lyophilized powder for injection, which needs to be formulated before intravenous drip.

Since this product does not contain preservatives, please use aseptic operations when formulating the drug preparation.

- Each vial of lyophilized powder is quantitatively reconstituted in 5 mL of distilled water for injection. During the operation, the distilled water is slowly added into the vial along the vial wall. Please do not directly drop the distilled water onto the surface of the lyophilized powder (the post-reconstitution concentration is 40 mg/mL)
- Do not violently shake the vial during reconstitution. Instead, reconstitute the powder in a gentle way with slow vortex. After reconstitution, allow the vial to stand for 6 min to allow the foam to disappear.
- The presence or absence of particles and discoloration in the liquid should be visually
- Draw a corresponding volume of the reconstituted solution from the vial and dilute it in 100 mL of 5% glucose for injection. Avoid generating a large number of air bubbles during the dilution process. After dilution, slowly invert the infusion bag several times to mix well. Maintain the final concentration at between 0.5 mg/mL and 10 mg/mL.
- Within 2 hours after the dilution is completed, an infusion set equipped with an online filter (0.2 μM) is used to finish the injection via intravenous drip. After the drug infusion is completed, use 20 mL of 5% glucose for injection to flush the drug remaining in the infusion line into the body. Do not use this infusion line to administer other drugs. Each infusion takes 30 min (not less than 20 min, no more than 60 min) (including the final flushing).

5.3 Formulation of Eribulin

Extract the required eribulin from a disposable bottle of cillin and injected into the syringe at the dose aseptically, and injected intravenously within 2-5 minutes, or diluted into 100ml 0.9% Sodium Chloride Injection and intravenously.

- It should not be diluted in a glucose solution or administered through an IV line containing a glucose solution. It is not allowed to be administered in the same infusion tube with other drugs.
- The undiluted eribulin is stored in a syringe at room temperature for a maximum of 4 hours or in cold storage (4℃) for a maximum of 24 hours. Diluted eribulin solutions can be stored up to 4 hours at room temperature or up to 24 hours in cold storage.

- Discard the unused parts of the bottle.

5.4 Dosing Regimen

Camrelizumab 200mg (3mg/kg for patient whose weight is below 50kg) will be administered as an intravenous infusion over 30 minutes every three weeks until unacceptable toxic effects or disease progression or other termination criteria appeared. Patients received up to two years of treatment.

Apatinib is taken orally after meals, once a day with one tablet (250 mg/tablet). Apatinib will be administered daily until unacceptable toxic effects or disease progression or other termination criteria appeared. Patients received up to two years of treatment.

Eribulin Mesylate will be administered as a 1.4 mg/m^2 intravenous (IV) injection over 2 to 5 minutes on day 1 and day 8 of each 21-day cycle until unacceptable toxic effects or disease progression or other termination criteria appeared.

Definition of postprandial administration: Dosing within 30 min after the end of a meal. The subjects continue to use the study drugs until the criteria for treatment discontinuation specified in the protocol are met.

5.5 Dose Modification

The dose of the study drug may be suspended, reduced or terminated according to the toxic side effects of the study drug occurring in the study;

- During the study period, the dose of camrelizumab is allowed to be suspended, and the maximum period of treatment delay is 8 weeks. If the administration of camrelizumab is delayed by more than 3 days after the planned time of administration, the drug should no longer be administered for that time point, and the dosing is resumed at a 200 mg dose (3mg/kg for patients whose weight is blow 50kg) by the next scheduled time of drug administration.
- Dose modifications caused by apatinib-related toxicity include: dose suspension (no more than 28 days), dose reduction and dose termination. During the study, apatinib only allowed dose reduction, which could be reduced from 250 mg/d to 125 mg/d, and the dose increase was not allowed. In the event of apatinib-related toxic side effects, the dose should be suspended first. After the toxicity has returned to an acceptable level, the following options may be selected as appropriate: resume dosing using the original dose, adjust the method of administration or terminate the dose. After the termination of apatinib administration, the subjects can continue their treatment using the camrelizumab monotherapy.
- Dose adjustments due to toxicity associated with eribulin mesylate injection include:

♦ Patients with liver function impairment

♦ Impaired liver function due to liver metastasis:

For patients with mild liver function impairment (Child-Pugh A), the recommended dose of this product is 1.1 mg/m^2, intravenously administered within 2-5 minutes, on a cycle of 21 days, once on the Day 1 and Day 8 of each cycle. For patients with moderate liver function impairment (Child-Pugh B), the recommended dose of this product is 0.7 mg/m^2, intravenously administered within 2-5 minutes, on a cycle of 21 days, once on Day 1 and Day 8 of each cycle. Severe liver function impairment (Child-Pugh C) has not been studied, but if these patients receive eribulin, the estimated dose would need to be reduced more significantly.

- Impaired liver function due to cirrhosis:

This group of patients has not yet been studied. The above doses can be used in patients with mild to moderate liver impairment, but it is recommended that they be closely monitored as doses may need to be readjusted.

- Patients with renal damage

For patients with moderate or severe renal impairment (creatinine clearance rate (CLCR) 15-49 ml/min), the recommended dose of this product is 1.1 mg/m^2, administered intravenously within 2-5 minutes on a 21-day cycle, once on Day 1 and Day 8 of each cycle.

Table 1 Regulation for Dose Modification of Camrelizumab and Apatinib

<table>
<tr><th colspan="2" rowspan="2">Drug-Related Toxicities</th><th rowspan="2">Grade</th><th colspan="2">Whether to Suspend the Dose</th><th rowspan="2">Criteria for Resuming Drug Administration</th><th rowspan="2">Method of Dose Modification</th><th rowspan="2">Criteria for Termination Drug Administration</th></tr>
<tr><th>Camrelizumab</th><th>Apatinib</th></tr>
<tr><td rowspan="6">Toxicity related to camrelizumab and apatinib</td><td rowspan="3">Hematological toxicity</td><td>Grade 1 and 2</td><td>No</td><td>No</td><td>—</td><td>—</td><td>—</td></tr>
<tr><td>Grade 3</td><td>Yes</td><td>Yes</td><td>After the toxicity is returned to ≤ Grade 2</td><td>Resume the dosing at the original dose</td><td rowspan="2">Apatinib discontinuation for more than 28 days; camrelizumab discontinuation for more than 8 weeks</td></tr>
<tr><td>Grade 4</td><td>Yes</td><td>Yes</td><td>After the toxicity is returned to ≤ Grade 2</td><td>Dose reduction of apatinib to 125mg/d</td></tr>
<tr><td rowspan="3">Non-hematological toxicity</td><td>Grade 1</td><td>No</td><td>No</td><td>—</td><td>—</td><td>—</td></tr>
<tr><td>Grade 2 (last for ≥ 7 days)</td><td>Yes</td><td>Yes</td><td>After the toxicity is returned to ≤ Grade 1</td><td>Resume the dosing at the original dose</td><td rowspan="2">Apatinib discontinuation for more than 28 days; camrelizumab discontinuation for more than 8 weeks</td></tr>
<tr><td>Grade 3</td><td>Yes</td><td>Yes</td><td>After the toxicity is returned to ≤ Grade 1</td><td>Dose reduction of apatinib to 125mg/d</td></tr>
<tr><td>Toxicity related to camrelizumab</td><td>Capillary hemangioma</td><td>Grade 3</td><td>Yes</td><td>No</td><td>After the toxicity is returned to ≤ Grade 2</td><td>Resume the dosing at the original dose</td><td>camrelizumab discontinuation for more than 8 weeks</td></tr>
</table>

Drug-Related Toxicities		Grade	Whether to Suspend the Dose		Criteria for Resuming Drug Administration	Method of Dose Modification	Criteria for Termination Drug Administration
			Camrelizumab	Apatinib			
Toxicity related to apatinib	Hypertension	Grade 3 (after symotomatic treatment)	No	Yes	After the toxicity is returned to ≤ Grade 1	Dose reduction of apatinib to 125mg/d	Apatinib discontinuation for more than 28 days
	Proteinuria (without a significant increase in blood creatinine)	Grade 3 (24h urine protein quantification)	No	Yes	After the toxicity is returned to ≤ Grade 2	Dose reduction of apatinib to 125mg/d	Apatinib discontinuation for more than 28 days
	Hand and foot syndrome	Grade 3	No	Yes	After the toxicity is returned to ≤ Grade 1	Dose reduction of apatinib to 125mg/d	Apatinib discontinuation for more than 28 days
	Headache	Grade 2 (last for ≥ 7 days) or Grade 3	No	Yes	After the toxicity is returned to ≤ Grade 1	Dose reduction of apatinib to 125mg/d	Apatinib discontinuation for more than 28 days

Table 2 Regulation for Dose Modification of Eribulin

Drug-Related Toxicities	Method of Dose Modification
**A permanent reduction in the 1.4mg/m^2 dose of eribulin is required for any of the following reasons:**	1.1 mg/m^2
ANC < 500/mm^3 (last for ≥ 7 days)	
ANC < 1000/mm^3,with fever or infection	
Blood platelets count < 25000/mm^3	
Blood platelets count < 50000/mm^3, requiring a blood transfusion	
Grade 3/4 non-hematological toxicity	
Skips or delays the day 8 dose of eribulin in the previous cycle due to toxicity	
Any event requiring a permanent dose reduction occurs during the dose of 1.1 mg/m^2 period	0.7 mg/m^2
Any event requiring a permanent dose reduction occurs during the dose of 0.7 mg/m^2 period	Discontinue the treatment of eribulin
ANC= absolute neutrophil count<br>Toxicity was assessed according to the National Cancer Institute (NCI) Common Terminology Criteria for Adverse Events (CTCAE) version 4.03	

If subjects experience significant toxicity during the trial, such as Grade 3 and higher study drug-related toxicity, or Grade 2 non-hematological toxicity lasting for 2 weeks and more (except for

asymptomatic Grade 2 hypertension), abnormalities in laboratory test indicators (except for proteinuria of < 2 g/24 h), the investigator may, according to the tolerability of the subjects, decide to adjust the method of apatinib administration, i.e., 7d administration at the original dose followed by 7d of drug suspension and observation, in subsequent studies after treatment delay and recovery from toxicity.

In the course of the study and based on the above regulations for dose modification, the investigator may modify the dose appropriately by comprehensively considering the drug-related toxicity in the subjects (if a subject experiences multiple Grade 2 study drug-related toxicity and shows poor tolerance to the drugs, the dosing of apatinib can be maintained at the original dose by given intermittently with observation after treatment delay and recovery from toxicity).

Once the following occurs in the trial: hypertensive crisis, cerebral hemorrhage, ≥ Grade 2 pulmonary hemorrhage, other hemorrhages of ≥ Grade 3, arterial thrombosis, Grade 4 venous thrombosis, leukoencephalopathy syndrome, gastrointestinal perforation, the administration of apatinib should be terminated and the administration of camrelizumab should be suspended along with active symptomatic treatment. The subsequent resumption of camrelizumab treatment will depend on the toxicity recovery of the subjects.

For Grade 4 diarrhea/colitis, ≥ Grade 3 increase in AST, ALT or bilirubin, ≥ Grade 3 injection reactions, pneumonia, nephritis, renal failure or other Grade 4 drug-related non-hematological toxicity occurring during the trial, the subjects should terminate the use of the drugs (camrelizumab and apatinib).

5.6 Drug Management, Dispensation and Return

The management, dispensation, and return of the drugs used for this clinical trial should be the responsibility of designated study staff. The investigator must ensure that all study drugs are only used for subjects participating in this clinical study. The dosage and administration should follow the trial protocol. The remaining or expired drugs should be returned to the funder. The drugs for clinical use should not be transferred to any non-clinical trial participant.

The study drugs should be stored according to the drug storage conditions detailed in the drug information. During the dispensation of the drug, a drug receipt form should be signed by both parties, one copy for the clinical study center and one copy for the funder. Remaining drugs and empty boxes are retrieved at the end of the study and a drug retrieval form will also be signed by both parties. The dispensation and return of all drugs should be recorded on designated record forms in a timely manner.

The clinical research associate is responsible for monitoring the supply, usage, and storage of the investigational drug, and the management of remaining drugs.

5.7 Combined Medication

5.7.1 Medications that are prohibited during the study and medications that should be used with caution

Drugs that May Have Drug-Drug Interactions with Apatinib:

In vitro studies have shown that apatinib is primarily metabolized by the liver P450 enzyme CYP3A4. Apatinib has a strong inhibitory effect on CYP3A4 and CYP2C9, and has a moderate inhibitory effect on CYP2C19. CYP3A4 inducers (dexamethasone, carbamazepine, rifampicin and phenobarbital) and inhibitors (ketoconazole, itraconazole, erythromycin and clarithromycin), CYP3A4 substrates (simvastatin, cyclosporine and pimozide), and other drugs metabolized via CYP3A4 (such as benzodiazepines, dihydropyridine, calcium ion antagonist and HMG-COA reductase inhibitors) should be used with caution during the treatment. Omeprazole should be used with caution during the treatment (except when omeprazole must be used to treat serious adverse drug reactions).

The substrates of CYP2C9 and CYP2C19 should be used with caution, as shown in the table below.

P450 Enzyme	Substrate
CYP2C9	Diclofenac, phenytoin, piroxicam, S-warfarin, and tolbutamide
CYP2C19	Diazepam, imipramine, lansoprazole, and S-mephenytoin

Drugs that Prolong the QT Interval of the Heart:

Because tinib drugs can trigger toxic side effects to prolong the QT interval in clinical applications, it is required to cautiously use the drugs whichcan prolong the QT interval with caution during the study.

These mainly include, but are not limited to, the following categories of drugs:

- Antibiotics: fluoroquinolones: sparfloxacin, gatifloxacin, levofloxacin, moxifloxacin, ofloxacin, ciprofloxacin; macrolides: erythromycin, clarithromycin, telithromycin, azithromycin, roxithromycin, metronidazole
- Antiarrhythmics: quinidine, procainamide, disopyramide, flecainide, propafenone, amiodarone, dronedarone, sotalol, dofetilide, and ibutilide
- Drugs used to relieve angina pectoris: ranolazine, ivabradine
- Antipsychotics: risperidone, fluphenazine, droperidol, haloperidol, thioridazine, pimozide, olanzapine, and clozapine
- Antifungal drugs: voriconazole, posaconazole
- Antimalarial drugs: mefloquine, chloroquine
- Antihistamines: terfenadine, astemizole, hydroxyzine
- Gastrointestinal drugs: antiemetics: ondansetron, granisetron, dolasetron, droperidol (0.625 to 1.25 mg may be a safe dose), hydroxyzine; prokinetics: cisapride, domperidone, metoclopramide

• Antidepressants: amitriptyline, imipramine, clomipramine, dosulepin, and doxepin

CYP3A4 substrates prohibited during the trial (drugs with narrow safety windows and may cause serious adverse reactions after their metabolism is affected) include but are not limited to:

- Hypoglycemic agents: tolbutamide, chlorpropamide
- Ergot derivatives: dihydroergotamine, ergometrine, ergotamine, methyl ergometrine (potential risk of ergot poisoning, including severe vasospasm leading to peripheral and cerebral ischemia)
- Antipsychotic: pimozide (can potentially increase the risk of prolonging QT interval)
- Antiarrhythmics: amiodarone (prohibited within 6 months prior to randomization), bepridil, flecainide, lidocaine, mexiletine, quinidine, propafenone
- Immunomodulators: cyclosporine, tacrolimus, sirolimus (can potentially increase the risk of nephrotoxicity and neurotoxicity)
- Miscellaneous: quetiapine, risperidone, clozapine, tomoxetine hydrochloride

If warfarin anticoagulant is used during the trial, its dose reduction should be considered and monitored closely, and the use of the investigational drugs should be stopped if necessary.

During the treatment period, anti-tumor drugs and adjuvant drugs related to tumor treatment, such as anti-tumor traditional Chinese medicine (see Appendix I for a detailed list), immunological agents, etc., should be discontinued.

5.7.2 Drugs and Treatments that May be Used as Appropriate during the Study:

In other cases, the subjects should be given an optimal supportive care during the treatment. Clinical concurrent diseases and various adverse reactions, especially immune-related adverse reactions, should be actively treated.

Patients can receive bisphosphonate for the treatment of bone metastases. If systemic or local analgesia is not effective in controlling painful lesions of bone metastases, a small area of palliative radiotherapy (the area of the radiotherapy must be < 5% of the bone marrow region, and the percent bone marrow in human skeleton is shown in the figure of **Appendix VI**) is allowed.

Palliative treatment for lesions outside the lungs and liver is allowed during the trial (when the treatment of the subjects is needed to improve symptoms upon the onset of PD), and the treatments include the treatments for the chest, ascites, pericardial effusion, and radiotherapy for brain lesions. During the treatment, the subjects should suspend the administration of the investigational drugs until the end of the recovery period of palliative treatment.

All concomitant treatments and drugs used within 30 days prior to the first dose and during the study period should be recorded in the eCRF in strict accordance with the GCP regulations. Subjects

should be closely monitored if adverse reactions occur, and active symptomatic treatment should be given if necessary. The drugs used should be documented and described in the CRFs.

6 Study Procedures

Before the study commences, the subjects must read and sign the current informed consent form approved by the ethics committee (EC). All examinations and trial procedures are carried out according to the time schedule of activities, and are not affected by the duration of drug suspension. However, it is allowed to change within the window period of test items due to holidays, weekends or other management reasons.

6.1 Screening Period (D-21–D-1):

Unless otherwise stated, the following screening steps must be completed within 21 days prior to the first dose:

[Signing Informed Consent] The written consent form of the subjects must be obtained in advance before any procedures of the clinical trial are carried out.

[Demographics] Data: gender, date of birth, ethnicity, height, weight, smoking history.

[Tumor history] including pathological diagnosis and clinical diagnosis:

1. Tumor diagnosis: the date of the first diagnosis of the tumor, histological classification, site of the primary tumor and metastatic lesions, pathological staging, clinical staging;
2. History of surgery: the surgical history of primary lesions, the surgical history of metastatic lesions;
3. History of chemotherapy (including new/adjuvant chemotherapy): the history of systemic chemotherapy, the history of targeted drug therapy, the history of local chemotherapy;
4. History of radiotherapy: date, dose, site (systemic/local);
5. ER/PR/HER2 assay results;
6. The date of PD or disease recurrence after the last systemic treatment.

[Concomitant Disease] Concomitant disease and related treatment history (such as diabetes, hypertension and other chronic diseases), history of tumors other than breast cancer.

[Concomitant Medication] Record the concomitant medication and concomitant treatment given within 30 days prior to the start of dosing and during the study period. Once a subject interrupts the trial treatment, only the concomitant medication and treatment used to solve new or unresolved adverse events related to the trial treatment should be recorded.

[Thyroid Function] Including serum FT3, FT4, and TSH.

[Examination of the Pituitary Adrenal Axis] Including ACTH and cortisol.

[Hepatitis B, Hepatitis C, and HIV Tests] 5 items of hepatitis B examination. If the test results are abnormal, virus replication (HBV DNA) test should be performed; hepatitis C virus antibody (anti-HCV) and HIV antibody tests.

[Adverse Events] Adverse events (AEs) should be recorded from signing the informed consent until at least 30 days after the last dose and should be followed-up until the AEs are resolved or stabilized. SAE and irAE observed within 90 days after the last dose of camrelizumab should be followed up. If the subjects start a new anti-tumor treatment, they should be followed-up until they start the tumor treatment (if the tumor treatment is started within 30 days after the last dose, the subjects should be followed-up until at least 30 days after the last dose; if the tumor treatment is started more than 30 days after the last dose, the subjects should be followed-up until they start the new tumor treatment).

[Blood Pressure Monitoring] The blood pressure measurement of subjects is performed by the investigator during the screening period; during each blood pressure measurement, smoking and coffee are prohibited within 30 min before the measurement, and subjects should at least rest for 10 min. The sitting position is taken during the measurement by placing the elbow at the same level as the heart. Each blood pressure measurement is taken on the same side of the body.

[Imaging Examination] Including CT or MRI of the chest, abdomen, and brain. The baseline tumor evaluation during the screening period can be relaxed to 3 weeks before treatment. Qualified CT/MRI scan results obtained before signing the informed consent can be used for tumor evaluation during the screening period. Bone scan should be performed upon clinically suspected bone metastases. Absence of cerebral hemorrhage should be confirmed within 21 days before randomization for patients with stable brain metastases.

[Biomarker Collection/Harvest] The existing paraffin-embedded tumor samples or fresh biopsy specimens are collected to make ≥ 8 slices that are 5 μm thick. Tumor samples from metastases or recurrent tumors were acquired to confirm the diagnosis of TNBC, and to determine the expression of PD-L1 or other biomarkers on ICs or TCs. The samples were obtained at fresh biopsy or 6 months prior to study enrollment.

Unless otherwise stated, the following screening steps must be completed within 7 days prior to the first dose:

[Blood Routine] White blood cell count (WBC), absolute neutrophil count (ANC), lymphocyte count (LYM), red blood cell count (RBC), hemoglobin (Hb), and platelet count (PLT).

[Urinalysis] Urine protein (Note: if urine protein ≥ 2+, it is required to check 24-h urine protein quantification), urine red blood cells, urine white blood cells, urine glucose.

[Stool Routine] Occult blood. A re-examination is needed for fecal occult blood+ and agastroscopy examination may be required. Performed within 7 days before enrollment, on Day 8 of Cycle 1 and Day 1 of subsequent cycles, and at the end of study.

[Blood Biochemistry] Total bilirubin, conjugated bilirubin, ALT, AST, AKP, γ-GT, total protein, albumin, blood urea nitrogen, creatinine, uric acid, fasting blood glucose, triglycerides, cholesterol, potassium, sodium, chlorine, calcium, phosphorus, blood lipase (only checked in the screening period and in case of subsequent abdominal pain, abdominal distension and other symptoms of suspected pancreatitis), blood amylase (only checked in the screening period and subsequent abdominal pain, abdominal distension and other symptoms of suspected pancreatitis).

[Coagulation Function] Including INR, APTT, PT, TT, and FIB.

[Myocardial Zymogram Examination] Including CK, LDH, and CK-MB. One examination should be performed within 7 days before enrollment. Then, this examination is only supplemented upon the onset of precordial pain and palpitations, as well as abnormal electrocardiograms. It is also carried out at the end of treatment/withdrawal.

[Pregnancy Test] The urine pregnancy test is performed 72 h before the first dose in women of childbearing potential. If the result of the urine pregnancy test is positive, a serum pregnancy test is performed. If necessary, re-tests can be performed for confirmation.

[Vital Signs] Body temperature, heart rate, respiratory rate, blood pressure.

[Physical Examination] General conditions, head and face, skin, lymph nodes, eyes (sclera, pupil), ear, nose, throat, respiratory system, cardiovascular system, abdomen (including liver and spleen), genitourinary system, musculoskeletal, nervous system, mental status.

[ECOG PS Score] See Appendix II.

[12-Lead ECG] If there is an abnormality, it should be repeated twice for confirmation, or the investigator will determine whether to add other necessary examinations.

[Echocardiography]

[Tumor Marker Blood Sampling] In the baseline period (before drug administration), 10 mL of blood sample are collected into a lithium heparin blood collection tube and centrifuged at 1500 g (centrifugal force) for 10 min in a low temperature centrifuge (4 °C). The supernatant is pipetted and the blood cells are retained, completely recorded, and stored at -70 °C or below.

The inclusion and exclusion criteria are verified again. Subjects must meet all inclusion criteria and must not meet any of the exclusion criteria before they can be included in the study.

6.2 Treatment Period

D1 of Cycle1 [Vital signs] [Physical examination] [Intravenous drip of camrelizumab] [Dispensation of apatinib] [Administration of apatinib] [Administration of eribulin]

In order to improve the compliance of the subjects and ensure that apatinib is taken after breakfast every day, only camrelizumab is administered on D1 of Cycle 1. Eribulin is administered on D1 and D8 of Cycle 1, while the oral administration of apatinib is started on D2 after breakfast.

Within 24 h after the first dose, the subjects should be closely monitored for acute allergic reactions. If an acute allergic reaction occurs, it should be treated according to the medical practice of the hospital and relevant guidelines.

The administration of apatinib is started from Cycle1 and its dose can be suspended or modified according to the occurrence of adverse events.

D1 of subsequent cycles [Blood routine] [Blood biochemistry] [Urinalysis] [Stool routine][Coagulation function] [Vital signs] [Physical examinations and weight measurement] [ECOG PS score] [ECG] [Intravenous drip of camrelizumab] [Injection of eribulin] [Adverse events] [Concomitant medication][Return/dispensation of apatinib]

D8 of Cycle1 [Blood routine] [Blood biochemistry] [Urinalysis] [Stool routine][Coagulation function] [Vital signs] [Physical examinations] [ECG] [Injection of eribulin] [Adverse events] [Concomitant medication]

D1 of subsequent cycles: [Blood routine] [Blood biochemistry] [Urinalysis] [Coagulation function] [Vital signs] [Physical examination] [ECG] [Intravenous drip of camrelizumab] [Injection of eribulin] [Adverse events] [Concomitant medication]

A window period of ± 3d is set and the administration of camrelizumab should be carried out after the evaluation of examinations and tests specified in the flowchart is completed.

[Imaging Evaluation]During the treatment period, the imaging examination should be performed under the same conditions as those of the baseline examination (layer thickness of the scan, use of contrast agent, etc.), and the lesions found at baseline should be checked every 2 cycles in the first 8 cycles of treatment (bone scan is done in case of suspected bone progress or for CR confirmation). After 8 cycles, imaging examination can be performed every 3 cycles and can be done as appropriate upon the discovery of suspected new lesions. The first PR/CR must be confirmed after 4 to 6 weeks; the first PD must be confirmed after 4 to 6 weeks (except for significant changes in subjects' symptoms or rapid tumor progression). The patients who were judged by investigators to have potential pseudo progression must be confirmed by a second radiologic scan 4 weeks later.

[Thyroid Function Test] Performed on Day 1 of Cycle 2 and then once every 3 cycles.

[Biomarker Collection] For subjects showing CR/PR during the trial, an additional biomarker blood sample collection should be done upon the onset of CR/PR and PD, respectively. The collection requirements are the same as those in the baseline period.

6.3 End of Treatment/Withdrawal

A subject will terminate the treatment upon the occurrence of an event that meets the "4.3.1 Criteria for Subject Withdrawal/Treatment Discontinuation". At the end of the study treatment or upon withdrawal from the study, if a subject has not undergone examinations within 14 days prior to the end of the study, the subject should undergo the following examinations:

[Blood routine] [Blood biochemistry] [Urinalysis] [Stool routine] [Coagulation function][Pregnancy test] [Thyroid function test] [Myocardial zymogram] [Vital signs] [Physical examination] [ECOG PS score] [ECG] [Echocardiography] [Blood pressure monitoring] [Adverse events] [Concomitant medication] [Return of apatinib]

If a subject has not undergone imaging examinations within 4 weeks prior to the end of the study, the subject should undergo an imaging examination at the end of the study treatment or upon withdrawal from the study. For subjects with PD demonstrated by non-imaging evidence (intolerability, other conditions), a tumor evaluation is carried out every 3 months until PD, death, or the initiation of other tumor treatments.

6.3 Follow-Up Period

30 days after termination/end of treatment

[Vital signs] [Physical examination] [ECOG PS score] [Blood routine] [Urinalysis] [Blood biochemistry] [Adverse events] [Concomitant medication]

SAE and irAE observed within 90 days after the last dose of camrelizumab should be followed up. If the subjects start a new anti-tumor treatment, they should be followed-up until they start the tumor treatment (if the tumor treatment is started within 30 days after the last dose, the subjects should be followed-up until at least 30 days after the last dose; if the tumor treatment is started more than 30 days after the last dose, the subjects should be followed-up until they start the new tumor treatment).

[Survival Follow-up] After the trial treatment is discontinued, the survival status and subsequent anti-cancer treatment can be collected through clinical or telephone follow-ups every 2 months until death.

Schedule of Activities

Item	Screening Period		Treatment Period (21days/cycle)				Post-Treatment		Survival Follow-Up
	Within 3 Weeks Before Dosing	Within 1 Week Before Dosing	Cycle 1		Cycles 2–26				
			Day 1	Day 8 (±3d)	Day 1 (±3d)	Day 8 (±3d)	End of Treatment/ Withdrawal	Visit after End of Treatment	Every 2 Months (±3d)
Baseline Data									
Signing Informed Consent	×								
Demographic Data	×								
Tumor History / Other Medical History[1]	×								
Concomitant Medication[2]	×	×	×						
Laboratory Tests									
Blood Routine [3]		×		×	×	×	×	×	
Urinalysis [4]		×		×	×	×	×	×	
Routine Stool Test [5]		×		×	×		×		
Blood Biochemistry [6]		×		×	×	×	×	×	
Coagulation Function Test [7]		×		×	×		×		
T3, FT3, FT4 and TSH [8]	×				×		×		
Pituitary Adrenal Axis Test [9]	×								
Myocardial Zymogram [10]		×					×		
Hepatitis B, Hepatitis C and HIV Test [11]	×								
Pregnancy Test [12]		×					×		

Item	Screening Period		Treatment Period (21days/cycle)				Post-Treatment		Survival Follow-Up
	Within 3 Weeks Before Dosing	Within 1 Week Before Dosing	Cycle 1		Cycles 2–26				
			Day 1	Day 8 (±3d)	Day 1 (±3d)	Day 8 (±3d)	End of Treatment/ Withdrawal	Visit after End of Treatment	Every 2 Months (±3d)
**Clinical Evaluation and Examination**									
Adverse Events [13]	**From signing the informed consent form until 30 days after the last dose**								
Vital Signs [14]		×	×	×	×	×	×	×	
Physical Examination [15]		×	×	×	×	×	×	×	
ECOG PS Score		×			×		×	×	
Electrocardiogram [16]		×		×	×	×	×		
Echocardiography [17]		×					×		
Blood Pressure Monitoring [18]	×	×		×	×	×	×		
**Study Drugs**									
Camrelizumab Administration [19]			×		×				
Apatinib Administration [20]			**Orally administration once a day after meal**						
Dispensation/Return of Apatinib [21]			×		×		×		
Eribulin Administration [22]			×	×	×	×			
**Imaging Evaluation**									
Imaging Examination [23]	×		**Once every 2 cycles in the first 8 cycles of dosing, followed by once every 3 cycle**				×		
**Follow-Up after the Treatment**									
Time to Progression [24]							Imaging evaluation is carried out once every 3 months (±7 d) until disease progression or start of new anti-tumor treatments (subjects with non-imaging PD)		
Time to Death [25]									×

Item	Screening Period			Treatment Period (21days/cycle)			Post-Treatment		Survival Follow-Up
	Within 3 Weeks Before Dosing	Within 1 Week Before Dosing	Cycle 1		Cycles 2–26				
			Day 1	Day 8 (±3d)	Day 1 (±3d)	Day 8 (±3d)	End of Treatment/ Withdrawal	Visit after End of Treatment	Every 2 Months (±3d)
Blood Sample Collection and Tumor Sample Collection/Harvest									
Collection/Harvest of Biomarkers [26]		×			×				

Notes:

[1] Tumor history/other medical histories: Pathological findings, ER/PR/HER2 detection report; a history of tumor surgery, chemotherapy, radiotherapy, and other disease treatments; tumor history other than breast cancer.

[2] Record the concomitant medication and concomitant treatment given within 30 days prior to the start of dosing and during the study period. Once a subject interrupts the trial treatment, only the concomitant medication and treatment used to solve new or unresolved adverse events related to the trial treatment should be recorded.

[3] Blood routine: Hemoglobin, red blood cell count, white blood cell count, neutrophil count, lymphocyte count, and platelet count are measured within 7 days before enrollment, on Day 8 of Cycle 1, Day 1 and Day 8 of subsequent cycles, at the end of study, and 30 days after the treatment.

[4] Urinalysis: Urine protein, urine glucose, urine occult blood (urine red blood cells, white blood cells). If the protein results of two consecutive semi-quantitative methods are 2+, a quantitative test of 24-h urine protein is performed. If the semi-quantitative method shows that the protein is > 2+, the quantitative test of 24-h urine protein is performed; carried out within 7 days before the enrollment, on Day 8 of Cycle 1, Day 1 and Day 8 of subsequent cycles, at the end of study, and 30 days after the treatment.

[5] Routine stool test: Occult blood; re-examination is needed for fecal occult blood+ and a gastroscopy examination may be needed based on the judgment of the investigator. Performed within 7 days before enrollment, on Day 8 of Cycle 1 and Day 1 of subsequent cycles, and at the end of study.

[6] Blood biochemistry: total bilirubin, conjugated bilirubin, ALT, AST, AKP, γ-GT, total protein, albumin, urea/blood urea nitrogen, creatinine, uric acid, fasting blood glucose, TG, cholesterol, potassium, sodium, chlorine, calcium, phosphorus, blood lipase (only during the screening period and in case of subsequent abdominal pain, abdominal distension and other symptoms of suspected pancreatitis), blood amylase (only during the screening period and subsequent abdominal pain, abdominal distension and other symptoms of suspected pancreatitis). It is carried out within 7 days before enrollment, on Day 8 of Cycle 1, Day 1 and Day 8 of subsequent cycles, at the end of study, and 30 days after the treatment.

[7] Coagulation function: INR, APTT, PT, TT, FIB; Performed within 7 days before enrollment, on Day 8 of Cycle 1 and Day 1 of subsequent cycles, and at the end of study.

[8] Thyroid function test: Including serum FT3, FT4, and TSH. Performed within 21 days before dosing, on Day 1 of Cycle 2, and then once every 3 cycles.

[9] Pituitary adrenal axis test: Including ACTH, cortisol, and sex hormone, which are performed during the screening period.

[10] Myocardial zymogram examination: One examination should be performed within 7 days before enrollment. Then, this examination is only supplemented upon the onset of precordial pain and palpitations, as well as abnormal electrocardiograms. It is also carried out at the end of treatment/withdrawal.

[11] Hepatitis B, hepatitis C and HIV tests: 5 items of hepatitis B examination. If the test results are abnormal, virus replication (HBV DNA) test should be performed; hepatitis C virus antibody (anti-HCV) and HIV antibody test.

[12] Pregnancy test: The urine pregnancy test is performed 72 h before the first dose in women of childbearing potential. If the result of the urine pregnancy test is positive, a serum pregnancy test is performed. If necessary, re-tests can be performed for confirmation.

[13] Adverse events: Adverse events (AEs) should be recorded from signing the informed consent until at least 30 days after the last dose and should be followed-up until the AEs are resolved or stabilized. SAE and irAE observed within 90 days after the last dose of camrelizumab should be followed up. If the subjects start a new anti-tumor treatment, they should be followed-up until they start the tumor treatment (if the tumor treatment is started within 30 days after the last dose, the subjects should be followed-up until at least 30 days after the last dose; if the tumor treatment is started more than 30 days after the last dose, the subjects should be followed-up until they start the new tumor treatment).

[14] Vital sign examination: Body temperature, respiration, heart rate, and blood pressure. Performed within 7 days before enrollment, on Day 1 and Day 8 of Cycle 1, Day 1 and Day 8 of subsequent cycles, at the end of study, and 30 days after treatment.

[15] Physical examination: Examination of main body systems (head and face, skin system, lymph nodes, eyes, ears, nose, throat, oral cavity, respiratory system, cardiovascular system, abdomen, genitourinary system, musculoskeletal system, nervous system, and mental state). Physical examination is performed within 7 days before enrollment, on Day 1 and Day 8 of Cycle 1, on Day 1 and Day 8 of subsequent cycles, at the end of study, and 30 days after treatment.

[16] 12-Lead ECG: Performed within 7 days before enrollment, on Day 8 of Cycle 1, Day 1 and Day 8 of subsequent cycles, and at the end of study. Abnormalities in ECG must be confirmed twice via additional ECG tests.

[17] Echocardiography: An echocardiography examination is performed within 7 days prior to the enrollment and at the end of the study, and additional echocardiography examinations are required for abnormalities with clinical significance during the study period.

[18] Blood pressure monitoring: The blood pressure measurement of subjects is performed by the investigator during the screening period; during each blood pressure measurement, smoking and coffee are prohibited for 30 min before the measurement, and subjects should at least rest for 10 min, and the sitting position is taken during the measurement by placing the elbow at the same level as the heart. Each blood pressure measurement is taken on the same side of the body; during the trial, the blood pressure monitoring is performed by the subjects themselves and recorded in their diary card. Blood pressure is detected at least 3 times a week in the first 2 cycles. If the blood pressure is abnormal, the measurement is carried out every day; if the blood pressure is normal, the blood pressure measurement is carried out twice a week after 2 cycles; in addition, the blood pressure is measured by the investigator during each follow-up. During each blood pressure measurement, smoking and coffee are prohibited for 30 min before the measurement, and subjects should at least rest for 10 min, and the sitting position is taken during the measurement by placing the elbow at the same level as the heart. Each blood pressure measurement is taken on the same side of the body.

[19] Camrelizumab administration: Camrelizumab is administered via intravenous infusion (without prophylactics) at a fixed dose of 200 mg within 30 min (not less than 20 min, no more than 60 min), once every 3 weeks. Each cycle contains 3 weeks. Camrelizumab is administered until the occurrence of an event meeting the discontinuation criteria.

[20] Apatinib is taken orally after meals, once a day, continuously.

[21] Return and dispensation of apatinib. Apatinib is dispensed on Day 1 of Cycle 1. From Day 1 of Cycle 2, the return and dispensation of apatinib are done on Day 1 of each cycle. The remaining drugs are returned first to verify the dose actually taken before new study drugs are dispensed.

[22] Eribulin administration: Eribulin is administered via intravenous injection at a fixed dose of 1.4mg/m^2 within 2-5 min, on Day 1 and Day 8 of every 3 weeks. Each cycle contains 3 weeks. Eribulin is administered until the occurrence of an event meeting the discontinuation criteria.

[23] Imaging examination: including CT or MRI of the chest, abdomen, and brain. The baseline tumor evaluation during the screening period can be relaxed to 3 weeks before treatment. Qualified CT/MRI scan results obtained before signing the informed consent can be used for tumor evaluation during the screening period. Bone scan should be performed upon clinically suspected bone metastases. Absence of cerebral hemorrhage should be confirmed within 21 days before randomization for patients with stable brain metastases.

During the treatment period, the imaging examination should be performed under the same conditions as those of the baseline examination (layer thickness of the scan, use of contrast agent, etc.), and the lesions found at baseline should be checked every 2 cycles in the first 6 cycles of treatment (bone scan is done in case of suspected bone progress or for CR confirmation). After 6 cycles, imaging examination can be performed every 3 cycles and can be done as appropriate upon the discovery of suspected new lesions. The first PR/CR must be confirmed after 4 to 6 weeks; the first PD must be confirmed after 4 to 6 weeks (except for significant changes in subjects' symptoms or rapid tumor progression).

The window period for imaging examination schedule is ± 7 days. Unplanned imaging examinations can be performed when progressive disease (such as worsening of symptoms) is suspected.

[24] In addition to the progressive disease as evidenced by imaging, subjects who have ended the trial treatment for other reasons must be evaluated at the end of treatment if the imaging examination is not performed within 4 weeks prior to the end of the trial. At the same time, tumor efficacy is followed up every 3 months after the end of the trial until records confirm the progressive disease or initiation of new tumor treatments.

[25] Survival follow-up: After the trial treatment is discontinued, the survival status and subsequent anti-cancer treatment can be collected through clinical or telephone follow-ups every 2 months until death.

[26] Blood samples (20 mL) shall be collected from each subject at baseline and centrifuged to retain plasma, while the blood cells shall be cryopreserved below -70 °C. Subjects showing CR/PR during the trial must be subject to an additional biomarker blood sample collection upon the onset of CR/PR and PD, respectively, and the collection requirements are the same as those of the baseline period; the existing paraffin-embedded tumor samples or fresh biopsy specimens (section after fixation and paraffin-embedding) are collected to make ≥ 8 slices that are 5μm thick.

7 Safety Evaluation

7.1 Adverse Event (AE)

7.1.1 Definition of Adverse Event

An adverse event refers to any untoward medical event that occurs in a subject of clinical study after signing the informed consent form. Any adverse medical events that occur from starting drug treatment until 30 days after the last dose should be followed-up until the adverse events are resolved or stabilized. SAE and irAE observed within 90 days after the last dose of camrelizumab should be followed up. If the subjects start a new anti-tumor treatment, they should be followed-up until they start the tumor treatment (if the tumor treatment is started within 30 days after the last dose, the subjects should be followed-up until at least 30 days after the last dose; if the tumor treatment is started more than 30 days after the last dose, the subjects should be followed-up until they start the new tumor treatment). Regardless of whether or not considered as related to the study drugs, these adverse medical events are all judged as AEs. For abnormalities of the physical examination or laboratory tests that are already present during the baseline period, they are also judged as AEs if the severity of these abnormalities is increased after the subjects receive the drug treatment.

The investigator should record any AEs that occurred in detail, including the description of the AEs and all related symptoms, time of occurrence, severity, duration, measures adopted, and final result and outcome.

7.1.2 Criteria for AE Severity

The criteria for AE severity are based on the grading criteria for adverse drug reactions in NCI-CTCAE v4.03. The following criteria can be used as references if unlisted adverse reactions occur:

Grade I: Mild, asymptomatic or mild symptoms; clinical or laboratory abnormalities only; treatment not indicated.

Grade II: Moderate, minimal, local or noninvasive intervention indicated; limited age-appropriate instrumental activities of daily living (refer to preparing meals, shopping for groceries and clothes, using the telephone, managing money, etc.).

Grade III: Severe or medically significant but not immediately life-threatening; hospitalization or prolongation of hospitalization indicated; disabling; limiting self-care ADL. Self-care ADL: refer

to bathing, dressing and undressing, feeding self, using the toilet, taking medications, and not bedridden.

Grade IV: Life-threatening consequences; urgent intervention indicated.

Grade V: Death related to AEs.

7.1.3 Criteria for the Causality between AEs and Investigational Drug

AEs include all unexpected clinical manifestations. All the AEs occurring after the signing of the ICF must be reported in the form of a clinical report according to AE reporting, regardless of whether the AEs are related to the study drugs, whether the subject is allocated to the investigational drug group, and whether the subject has been administered with the drug. Any discomfort complained by the subject or changes in objective laboratory test measurements during the treatment should be truthfully recorded. Additionally, the severity, duration, management measures, and outcomes of the AE must be stated. The clinical physician should comprehensively determine the relationship between the AEs and investigational drug and assess the possible relationship as "definitely related, possibly related, unlikely related, not related, and not assessable". "Definitely related, possibly related, and not assessable" events are included as adverse drug reactions. The incidence of an AE is calculated with the sum of these three categories as the numerator and the number of subjects used for safety evaluation as the denominator. Criteria are as follows:

Table 7-1. Criteria for the causality between AEs and investigational drug.

Grade	Evaluation Criteria
Definitely Related	The AE occurs in a plausible time relationship to drug administration. The event is a recognized pharmacological phenomenon of the suspected drug. The event resolves with drug discontinuation and recurs with drug re-administration.
Possibly Related	The AE occurs in a plausible time relationship to drug administration. The event is a recognized pharmacological phenomenon of the investigational product. It can also be explained by patient's clinical status or other treatments.
Unlikely Related	The AE does not occur in a plausible time relationship to drug administration. The event is not a recognized pharmacological phenomenon of the investigational product. It can also be explained by patient's clinical status or other treatments.
Not Related	The AE does not occur in a plausible time relationship to drug administration. The event is not a recognized pharmacological phenomenon of the investigational product. It

	can also be explained by patient's clinical status or other treatments. The event resolved when patient's clinical status improved or other treatments are discontinued. The event recurs upon restarting other treatment.

7.2 Serious Adverse Event (SAE)

7.2.1 Definition of Serious Adverse Event

Serious adverse event (SAE) is a medical occurrence that results in hospitalization, prolonged hospitalization, disability, incapacity, life-threatening or death, or congenital malformation, etc. It includes the following unexpected medical events:

- Death;
- Life-threatening (defined as when the subject is at risk of death at the time of the event);
- Hospitalization or prolonged hospitalization;
- Permanent or serious disability/incapacity;
- Congenital anomalies or birth defects; or,
- Other important medical events requiring intervention to prevent permanent injuries or damages.

7.2.2 Progressive Disease

Progressive disease is defined as the worsening of subject condition caused by the primary tumor targeted by the investigational drug. It includes radiological progression and progressions in clinical signs and symptoms. New metastases relative to the primary tumor, or progression of the previous metastases, are recognized as disease progression. Life-threatening events, hospitalization or prolonged hospitalization, permanent or serious disability/incapacity/impairment of work ability, congenital anomalies or birth defects resulting from signs and symptoms of progressive disease should not be reported as SAEs on an expedited basis. Death caused by the symptoms and signs of PD is reported as an SAE on an expedited basis.

7.2.3 Abnormal Hepatic Enzymes

If the levels of AST and/or ALT are abnormal and meet the laboratory test abnormalities shown in the table below, such cases of hepatic enzyme abnormalities should be reported as SAEs. The investigator is required to enhance the follow-up of the subjects, who are followed up until their levels of hepatic enzymes become normal or reach the baseline levels.

Baseline Normal	Normal (AST/ALT and TBIL)	Abnormal (AST/ALT/TBIL)
Treatment Period	ALT or AST ≥ 3 × ULN<br>Total bilirubin ≥ 2 × ULN<br>Alkaline phosphatase ≤ 2 × ULN<br>No hemolysis	AST or ALT ≥ 2 × baseline, and ≥ 3 × ULN<br>Or AST or ALT ≥ 8 × ULN<br>With TBIL increase ≥ 1× ULN or value ≥ 3× ULN

7.2.4 Other Anti-Tumor Treatments

For subjects starting other anti-tumor treatments, adverse events except death will be reported until the start of the new anti-tumor treatment (30 days after the last dose, e.g., the new anti-tumor treatment is started within 30 days after the last dose). If a death occurs after the end of the study treatment but within the reporting period of serious adverse events, it must be reported promptly regardless of whether the patient received other treatments.

7.2.5 Hospitalization

During the clinical study, adverse events resulting in hospitalization or prolonged hospitalization should be considered as SAEs, not including non-medical hospitalization. Hospitalization or prolonged hospitalization not related to the worsening of adverse events is not considered an SAE. For example:

- Hospitalization due to existing disease without the occurrence of a new adverse event or worsening of the existing disease (e.g. to examine a persistent laboratory test abnormality that existed prior to the enrollment);
- Hospitalization for management reasons (e.g. annual routine physical examination);
- Hospitalization during the trial as specified in the study protocol (e.g. as required by the protocol);
- Elective hospitalization unrelated to the exacerbation of an adverse event (e.g., elective cosmetic surgery);
- Scheduled treatment or surgery should be documented throughout the trial protocol and/or subject's individual baseline data;
- Hospitalization merely due to the use of blood products.

Diagnostic or therapeutic invasive (e.g., surgery) or non-invasive procedures should not be reported as adverse events. However, the disease condition leading to such procedures should be reported if it meets the definition of AEs. For example, acute appendicitis during the adverse event reporting period should be reported as an adverse event. The appendectomy should be documented as the treatment method for the event.

7.2.6 Serious Adverse Event (SAE) Report

Serious adverse events that occur starting from the signing of the informed consent form until 90 days (inclusive) after the last dose of the study drugs should be reported. If the subjects start a new anti-tumor treatment, they should be followed-up until they start the tumor treatment (if the tumor treatment is started within 30 days after the last dose, the subjects should be followed-up until at least 30 days after the last dose; if the tumor treatment is started more than 30 days after the last dose, the subjects should be followed-up until they start the new tumor treatment). During the trial, in the event of an SAE, whether it is the first report or a follow-up report, the investigator must complete the "New Drug Clinical Trial Serious Adverse Event (SAE) Report Form" immediately, with a signature and date, and notify the clinical research associate, the Ethics Committee of the study center, the China Food and Drug Administration (CFDA), and the investigator's local (provincial or city) food and drug administration as well as the Medical Management Bureau of Health Administration within 24 h of knowing of the event.

SAEs that occur 90 days after the last dose are generally not reported unless suspected to be related to the investigational drug.

SAEs should be documented in detail, including symptoms, severity, date of onset, time of treatment, measures taken, time and method of follow-up, as well as outcome. If the investigator believes that an SAE is unrelated to the investigational drug but potentially related to study conditions (e.g., termination of the original treatment or complications during the trial), then this relationship should be detailed in the SAE page of the case report form. If the severity of an ongoing SAE or its relationship with the investigational drug changes, an SAE follow-up report should be submitted to the sponsor immediately. All SAEs should be followed until resolved or stabilized.

Table 7-2. Contact information for SAEs.

Unit	Contact Person	Fax/Telephone/Address
Sun Yat-sen Memorial Hospital of Sun Yat-sen University	Ethics Committee	Telephone/Fax:020-81332587
China Food and Drug Administration Division of Drug Research Supervision, Department of Drug and Cosmetics Registration	Address: Building 2, No. 26, Xuanwumen West Street, Xicheng District, Beijing<br>Postal code: 100053<br>Tel.: 010-88330732<br>Fax: 010-88363228	
Medical Administration Bureau, Health Administration	Address: No. 38, North Lishi Road, Xicheng District, Beijing (100810)<br>Tel.: 010-68792201<br>Fax: 010-68792734 (preferred)	
Medical Administration Bureau, Health Administration	Address: Beijing Food and Drug Administration, 12-14F, Zhonghuan Office Building A, 70 Zaolin Qian Street, Xicheng District, Beijing (100053)<br>Tel.: 010-83979465<br>Fax: 010-83560723	

7.3 Pregnancy

If a female subject becomes pregnant, the subject must be excluded from the study. The investigator must report to investigator within 24 hours of knowing the event. The investigator must continuously monitor on the outcome of the pregnancy until the 1 month after delivery. Negative pregnancy outcomes (stillbirth, spontaneous abortion, fetal malformation) are considered as SAEs and need to be reported according to the time requirements for SAEs.

If the subject also meets the SAE criteria, an SAE report form should also be filled and be reported in accordance with SAE requirements.

7.4 AE of Special Interest

For an AE of special interest specified in the clinical trial protocol, the investigator must report to the Ethics Committee of Sun Yat-sen Memorial Hospital of Sun Yat-sen University within 24 hours of event awareness. If it is an SAE at the same time, the "CFDA Serious Adverse Event Report" must also be completed.

- ≥ Grade 3 infusion reactions
- ≥ Grade 2 diarrhea/colitis, uveitis, interstitial pneumonia
- Other ≥ Grade 3 immune-related adverse events

- Any possible hepatic enzyme abnormalities (see **7.2.3**, the lack of other related causes of the abnormalities at the same time, such as PD, acute viral hepatitis, cholestasis, combined medication, previous concurrent liver disease, etc.)
- Grade 4 amylase or lipase elevation

7.5 Infusion Reactions

During the entire study, the investigator should closely monitor the possible infusion and/or allergic reactions, especially acute immune-mediated adverse reactions (including cytokine storms).

In general, there is no need for the administration of prophylactics before the infusion of camrelizumab. Based on published relevant data, an allergic reaction/allergy event is most likely to occur within 24 h after infusion. Once an allergic reaction/allergy event occurs, the infusion should be slowed or suspended according to the situation, and clinical supportive care should be given. In addition, prophylactics can be given before further administration. Possible allergic reactions may present as fever, chill, shiver, headache, rash, itching, joint pain, hypotension or hypertension, or bronchospasm. All grade 3 or 4 infusion reactions should be reported according to SAE procedures.

7.6 Immune-Related Adverse Events (irAEs)

Immune-related adverse events (irAEs) are clinically significant side effects that are consistent with the immunological mechanisms of the study drugs. irAEs require further serological, immunological and pathological (biopsy) data to support its diagnosis. Also, tumors, infections, metabolism, toxins, or other pathogenic factors must be ruled out.

Management Principles for Immune-Related Adverse Events (See Appendix IV for Details):

- **Immune-Related Pneumonitis**

In the clinical study of camrelizumab, the monitoring of signs and symptoms of immune-related pneumonitis, such as cough and chest discomfort, in the subjects will be strengthened. Examinations will be carried out via imaging methods (such as X-ray images), and a high-dose hormone therapy will be given to the subjects with Grade 2 or higher immune-related pneumonitis. Subjects with Grade 2 immune-related pneumonitis may temporarily suspend camrelizumab

for treatment, but subjects with Grade 3 or 4 immune-related pneumonitis should permanently terminate the camrelizumab treatment.

- **Immune-Related Enteritis**

In the clinical study of camrelizumab, the monitoring of signs and symptoms of immune-related enteritis, such as abdominal pain, diarrhea, and hematochezia, in the subjects will be strengthened.

A high-dose hormone therapy will be given to the subjects with Grade 2 or higher immune-related enteritis. Subjects with Grade 2 or 3 immune-related enteritis may temporarily suspend camrelizumab for treatment, but subjects with Grade 4 immune-related enteritis should permanently terminate the camrelizumab treatment.

- **Immune-Related Hepatitis**

In the clinical study of camrelizumab, the monitoring of signs and symptoms of immune-related hepatitis, such as liver discomfort and transaminase abnormal, in the subjects will be strengthened.

A high-dose hormone therapy will be given to the subjects with Grade 2 or higher immune-related hepatitis. Subjects with Grade 2 immune-related hepatitis may temporarily suspend camrelizumab for treatment, but subjects with Grade 3 or 4 immune-related hepatitis should permanently terminate the camrelizumab treatment.

- **Immune-Related Thyroid Dysfunction**

Thyroid dysfunction may occur at any time during the study. Therefore, in the clinical study of camrelizumab, the thyroid functions of the subjects will be regularly examined to closely monitor the clinical symptoms of abnormal thyroid functions. After the occurrence of immune-related hyperthyroidism, the subject should be given a high dose of cortisone/prednisone. Hormone replacement therapy is used for the treatment of hypothyroidism, but glucocorticoids are not applicable.

In the clinical study of camrelizumab, the monitoring of signs and symptoms of immune-related thyroid dysfunction in the subjects will be strengthened. A high-dose hormone therapy will be given to subjects with Grade 3 or higher immune-related thyroid dysfunction, but subjects with Grade 4 immune-related thyroid dysfunction should permanently terminate the camrelizumab treatment.

- **Immune-Related Nephritis and Renal Failure**

In the clinical study of camrelizumab, the monitoring of signs and symptoms of immune-related nephritis in the subjects will be strengthened. A high-dose hormone therapy will be given to the subjects with Grade 2 or higher immune-related nephritis. Subjects with Grade 2 immune-related nephritis can temporarily suspend camrelizumab for treatment, but subjects with Grade 3 or 4 immune-related nephritis should permanently terminate the camrelizumab treatment.

- **Immune-Related Hypophysitis**

In the clinical study of camrelizumab, the monitoring of signs and symptoms of immune-related hypophysitis in the subjects will be strengthened. A high-dose hormone therapy will be given to the subjects with Grade 2 or higher immune-related hypophysitis. Subjects with Grade 2 or 3 immune-related hypophysitis can temporarily suspend camrelizumab for treatment, but subjects with Grade 4 immune-related hypophysitis should permanently terminate the camrelizumab treatment.

- **Other Immune-Related Adverse Reactions**

In principle, depending on the severity of the adverse reactions, suspension of camrelizumab should be primarily used. If the severity of the AEs returns to Grade 1 or lower, the resumption of camrelizumab can be considered; In case of a serious Grade 3 or life-threatening Grade 4 adverse reaction, camrelizumab should be permanently discontinued.

Management of immune-related adverse reactions should be based on the medical practice and guidelines of the study site. The recommendations on treatment of irAEs are as follows for reference (Table 7-3).

Table 7-3. Recommendations on the treatment of immune-related adverse reactions by camrelizumab for injection

CTCAE Grade	Clinical Management*	Camrelizumab Treatment
Grade 1 (Mild)	-Close monitoring, especially for diarrhea<br>• Supportive care	Continue
Grade 2 (Moderate)	-Close monitoring<br>• Supportive care	Suspend, resume the drug when Grade ≤ 1

	• Local application of steroids, such as skin/colitis, etc.<br>If the symptoms are lasting for more than 7 days, start 1 mg/kg of prednisone equivalent.<br>If the symptoms are aggravated, intravenous or oral administration of 1 mg/kg prednisone	
Grade ≥ 3<br>(Severe)*	-2 mg/kg of prednisone equivalent	Permanent discontinuation

* The treatment does not need to be discontinued for Grade 3 local skin and endocrine diseases because they can often heal (skin) or be treated using an replacement therapy (endocrine)
(Weber, Jeffrey S. MD, PhD, et al., "Toxicities of immunotherapy for the practitioner", J Clin Oncol, April 2015)

7.7 Symptomatic Treatment for Apatinib-Related Adverse Reactions

1) Hand and Foot Syndrome (HFS)

Hand and foot syndrome is featured by a dysesthesia of hand and foot or acral erythema. It is skin toxicity that is more pronounced in compressed or pressured areas. It can occur in the tumor patients during the chemotherapy or molecular targeted therapy. HFSR is characterized by numbness, dysesthesia, paraesthesia, tingling, unable to feel pain, a painful feeling, swollen skin, erythema, desquamation, rhagadia, hardened blisters, and severe pain.

Grade of HFS:

Grade 1: Numbness/dysesthesia/paraesthesia, painless swelling or erythema of the hands and/or feet and/or discomfort that does not affect normal activities.

Grade 2: Painful erythema and swelling of the hands and/or feet and/or discomfort affecting patients' activities of daily living.

Grade 3: Moist desquamation, ulceration, blistering or severe pain of hands and/or feet and/or severe discomfort that causes the patients to be unable to work or perform activities of daily living. Strong pain, loss of skin function, relatively rare.

Symptomatic Treatment and Handling of HFS:

Some necessary symptomatic and supportive treatments must be taken, including: Strengthen skin care, keep skin clean, avoid secondary infections; Avoid pressure or friction; Use a moisturizer or lubricant, use lotion or lubricant containing urea and corticosteroids in local areas; Use local antifungal or antibiotic treatment if necessary.

Note: If there are 3 consecutive Grade 3 or higher hand and foot syndrome, and if the symptoms tend to become more aggravated, the dosing of the drug should be stopped and the subject should withdraw from the clinical study.

2) Hypertension

Before enrollment, the patients should strictly meet the blood pressure requirements specified in the inclusion/exclusion criteria. The blood pressure of patients with hypertension can be controlled by adjusting the dose of antihypertensive drugs or by adding a new antihypertensive drug before the administration of investigational drug. The blood pressure should be controlled within 140/90 mmHg (the average of 2 blood pressure monitoring operations that are at least 24 h apart) prior to randomization.

Monitoring and handling of such hypertension: Blood pressure should be monitored at least 3 times a week during the first 2 cycles of targeted drug therapy.

Since anti-VEGF/VEGFR targeted drugs can decrease the synthesis of NO and ultimately activate the renin-angiotensin-aldosterone system to cause hypertension, angiotensin converting enzyme (ACE) inhibitors (such as captopril, enalapril, benazepril, and cilazapril) are preferred for antihypertensive therapy. For some patients who are allergic or intolerant to ACE inhibitors, angiotensin II receptor blockers (ARB, such as losartan, valsartan, irbesartan, and telmisartan) can be used for treatment. In addition to lowering blood pressure, ARB is also beneficial for alleviating proteinuria. ACE inhibitors can be used in patients with chronic kidney diseases, proteinuria, and metabolic syndrome; dihydropyridine calcium ion antagonists are suitable for elderly patients.

When subjects develop hypertension or aggravated hypertension during drug administration, the following measures should be used: 1) adjust the study drugs according to the protocol; 2) start the administration of antihypertensive drugs or adjust the dose of antihypertensive drugs.

Antihypertensive drugs recommended for the trial: 1) angiotensin converting enzyme inhibitor (ACEI); 2) angiotensin II receptor blocker (ARB); 3) dihydropyridine calcium channel blocker; 4) β-receptor blocker.

It is not recommended to use diuretic antihypertensive drugs. Antihypertensive drugs with an inhibitory effect on CYP3A4, such as nicardipine, diltiazem, and verapamil, are prohibited during the administration period of the investigational drugs. For those with hypertensive crisis, the application of apatinib should be terminated and the subjects should withdraw from this clinical study.

3) Hemorrhage

Symptomatic treatment should be actively given for gastrointestinal hemorrhage, including fecal occult blood (++) and above, hematemesis or bloody stool. Patients with upper gastrointestinal hemorrhage should fast and be given acid suppression, gastric mucosal protection, and hemostatic treatment (tranexamic acid, reptilase, etc.), as well as octreotide if necessary;

Patients with lower gastrointestinal hemorrhage should be given hemostatic treatment, blood transfusion, and supportive care, etc.; For those whose bleeding cannot be controlled, assistance from the surgery department should be requested immediately.

Patients with hemoptysis should be given hemostatic treatment, blood transfusion, and supportive care, etc.; for those whose bleeding cannot be controlled, assistance from the surgery department should be requested.

Note: Patients with cerebral hemorrhage, Grade 2 or higher pulmonary hemorrhage, and Grade 3 or higher hemorrhage should stop the study treatment immediately, undergo symptomatic treatment and withdraw from the study.

4) Proteinuria

All subjects should be closely monitored for proteinuria throughout the entire treatment period and the monitoring should be strengthened for those with a history of hypertension; for those with two consecutive test results of urine protein ++ to +++, a 24-h urine protein assay is required. For those with urine protein ++++ and above, a 24-h urine protein assay is required.

Note: In case of nephrotic syndrome, the subject should discontinue the treatment permanently and withdraw from this clinical study.

5) Thrombosis

Subjects developing any arterial thrombosis (such as cerebral ischemia, stroke, angina pectoris, myocardial infarction, etc.) should stop the drug treatment immediately and withdraw from the clinical study. In case of any symptomatic Grade 4 venous thrombosis, the subjects should stop the drug treatment and withdraw from the clinical study. Once the symptoms are observed, symptomatic treatment, surgery, or anticoagulants shall be immediately given.

6) Reversible Posterior Leukoencephalopathy Syndrome

Reversible posterior leukoencephalopathy syndrome has not been reported in completed clinical trials of apatinib, but has been found in clinical application of newly marketed anti-angiogenic macromolecular antibodies and small molecule inhibitors.

The clinical manifestations are headache, altered consciousness/confusion, visual abnormalities/blindness, and convulsion, often accompanied by hypertension. The causes of reversible posterior leukoencephalopathy syndrome are yet unidentified. Once the symptoms are suspected, the investigational drug shall be discontinued immediately, and symptomatic treatment and strict blood pressure control shall be given. The subject will withdraw from the study once the imaging diagnosis is confirmed.

7) Fatigue and Weakness

Fatigue and weakness are common tumor-related clinical symptoms. Electrolyte disturbance, abnormal liver function, and abnormal cardiac function can all cause fatigue and weakness. Also, fatigue and weakness are common adverse reactions of targeted anti-angiogenic drugs, such as sunitinib, pazopanib, and sorafenib. Clinical reports show that targeted anti-angiogenic drugs may increase the incidence of fatigue and weakness through hypothyroidism.

In a previously completed clinical trial of apatinib, subjects in the test group showed a higher incidence of fatigue and weakness than those in the control group, and the mechanism behind the increased incidence of fatigue and weakness caused by apatinib is yet unidentified.

Therefore, close attention should be paid to Grade 2 or higher fatigue and weakness. In the case of Grade 3 or higher fatigue and weakness, the subject should be admitted to the hospital immediately for detailed examinations to exclude possible reasons such as electrolyte disturbance, abnormal liver function, cardiac dysfunction (ECG, echocardiography), and abnormal hormone

levels (adrenal hormones, thyroid hormones). The subject should receive a symptomatic treatment and the dose should be suspended or modified correspondingly according to the principle of dose modification.

8) Abdominal Pain

Abdominal pain is not uncommon in the treatment with apatinib, which are mostly a concomitant symptom of tumor. Also, gastrointestinal perforation occasionally occurs in clinical trials of apatinib and other anti-angiogenic drugs. For subjects with abdominal pain, the investigator shall be cautious of potential gastrointestinal perforation. Upon the observation of gastrointestinal perforation, the drug should be discontinued immediately, and the subject should withdraw from the trial and be given an active symptomatic treatment.

9) Interstitial Pulmonary Fibrosis

The clinical physicians shall fully understand the conditions of subjects and be familiar with the drugs that may lead to pulmonary toxicity. The clinical symptoms and changes in chest X-ray or CT are closely monitored. Once cough, chest tightness, labored breathing, dyspnea, and hemoptysis of unknown causes, the drug shall be discontinued as soon as other causes (such as infection and heart failure) are ruled out. Alveolar lavage and surgical lung biopsy are important means to diagnose interstitial lung disease. Currently, no therapy yields a satisfactory result against pulmonary fibrosis, and correction of hypoxemia and timely administration of corticosteroids are recommended following "Guidelines for Diagnosis and Treatment of Idiopathic Pulmonary (Interstitial) Fibrosis" issued by the Chinese Medical Association Respiratory Diseases Branch.

Note: Consult specialists when necessary.

7.8 Symptomatic Treatment for Erublin-Related Adverse Reactions

1) Haematology

Myelosuppression is dose dependent and primarily manifested as neutropenia. Monitoring of complete blood counts should be performed on all patients prior to each dose of eribulin. Treatment with eribulin should only be initiated in patients with ANC values $\geq 1.5\times10^9$/L and platelets > 100 $\times10^9$/L.

Febrile neutropenia occurred in < 5% of patients treated with eribulin. Patients experiencing febrile neutropenia, severe neutropenia or thrombocytopenia, should be treated according to the recommendations in section **5.5**.

Patients with ALT or AST >3 × ULN experienced a higher incidence of Grade 4 neutropenia and febrile neutropenia.

Although data are limited, patients with bilirubin >1.5 × ULN also have a higher incidence of Grade 4 neutropenia and febrile neutropenia.

Fatal cases of febrile neutropenia, neutropenic sepsis, sepsis and septic shock have been reported. Severe neutropenia may be managed by the use of granulocyte colony-stimulating factor (G-CSF) or equivalent at the physician’s discretion in accordance with relevant guidelines.

2) Peripheral neuropathy

Patients should be closely monitored for signs of peripheral motor and sensory neuropathy. The development of severe peripheral neurotoxicity requires a delay or reduction of dose (see section **5.5**)

In clinical trials, patients with pre-existing neuropathy greater than Grade 2 were excluded. However, patients with pre-existing neuropathy Grade 1 or 2 were no more likely to develop new or worsening symptoms than those who entered the study without the condition.

3) QT prolongation

In an uncontrolled open-label ECG study in 26 patients, QT prolongation was observed on Day 8, independent of eribulin concentration, with no QT prolongation observed on Day 1. ECG monitoring is recommended if therapy is initiated in patients with congestive heart failure, bradyarrhythmias or concomittant treatment with medicinal products known to prolong the QT interval, including Class Ia and III antiarrhythmics, and electrolyte abnormalities. Hypokalemia or hypomagnesemia should be corrected prior to initiating HALAVEN and these electrolytes should be monitored periodically during therapy. Eribulin should be avoided in patients with congenital long QT syndrome.

8 Efficacy Evaluation

The objective response rate (ORR), duration of response (DoR), disease control rate (DCR), clinical benefit rate (CBR), time to response, progression-free survival (PFS), and one-year overall survival rate are evaluated.

The tumor imaging evaluation is performed once every 2 cycles (6 weeks) based on RECIST v1.1 (**Appendix V**). For subjects with CP/PR for the first time, the results shall be confirmed after 4 weeks. The subjects who were judged by investigators to have potential pseudo progression must be confirmed by a second radiologic scan 4 weeks later. The tumor imaging evaluation is carried out once every 3 cycles (24 weeks), 6 months after the first dose of the drug.

9 Study Management

9.1 Ethics and Informed Consent

This clinical trial must comply with the "Declaration of Helsinki" (1996 edition), CFDA's "Good Clinical Practice" (GCP), and related regulations. Approval must be obtained from the Ethics Committee before the trial is initiated. During the trial, any revisions to this protocol must be reported to the ethics committee and put on record.

The clinical investigator must follow all applicable laws and regulations to protect the subjects. The informed consent form used in the informed consent process must be approved by the institutional review board and be available for inspection.

The clinical investigator must inform the subjects that the participation in the clinical trial is voluntary and that the subjects have the right to withdraw from the study at any stage of the study without being discriminated and retaliated, their medical treatment and benefits will not be affected, and they can continue to be treated by other approaches. All subjects shall be acknowledged that the participation of the trial and their personal information during the trial are kept confidential. Also, the subjects should be informed of the nature, objectives, expected potential benefits, and possible risks and inconvenience of the clinical trial, other alternative treatment options, and rights and obligations of the subjects in accordance with the "Declaration of Helsinki". Subjects are given sufficient time to consider whether to participate in the trial and sign the informed consent form.

Before the implementation of any process required by the study protocol, the subjects must:

- Be informed of information relevant to the study and all content and terms in the informed consent form;
- Be given sufficient time to ask questions and consider whether to participate in the study;
- Be enrolled in the study voluntarily;
- Sign and date the informed consent form approved by the IRB/IEC.

In the case of major changes during the study, the protocol should be amended. Unless it is necessary to eliminate obvious direct harms to subjects, the investigator must not make any change to the study without the approval of the IRB/IEC and sponsor. Changes to the study protocol intended to eliminate obvious direct risks to subjects can be implemented immediately, but the change must be recorded in protocol amendments, reported to the IRB/IEC, and submitted to relevant regulatory authorities within a required period. The process of protocol amendment must follow the same process of review and approval of the original protocol.

9.2 Protocol Amendments

The "Clinical Study Protocol" and "Clinical Study Case Report Form" are formulated by the PI and approved by the ethics committee of hospital before implementation. During the clinical trial, any changes to the study protocol must be approved by the ethics committee.

9.3 Quality Assurance of the Clinical Trial

In order to ensure the quality of the clinical trial, a clinical study plan should be formulated by the principal investigator before the official commencement of the study. All the relevant study staff in the clinical trial are trained for the study protocol and GCP.

The investigational drug in the clinical trial must be managed in accordance with the SOP, including receiving, storage, dispensing, and returning.

According to the GCP guidelines, necessary steps must be taken at the design and implementation phases of the study to ensure that all collected data are accurate, consistent, intact, and reliable. All observed results and abnormal findings in the clinical trial must be verified and recorded in a timely and serious manner to ensure data reliability. All instruments, equipment,

reagents, and standards used in various tests in the clinical trial must have stringent specifications and be operated under normal conditions.

9.4 Data Management

Data management is to ensure the reliability, integrity, and accuracy of the data, with an objective to obtain authentic data of high quality for statistical analysis. Study data will be collected and managed using the Case Report Form (CRF).

9.4.1 Review of Data and Monitoring of Study Center

The investigator must keep the source documents of each subject, including all medical records and visit records (outpatient or inpatient record), such as demographic indicators, medical information, lab results, ECGs, and results of other examinations and evaluations. All information on the CRF must come from the source documents of the subject. The investigator must also keep the informed consent forms signed by the subjects.

9.4.2 Keeping of Trial Record

To fulfill the review and/or audit requirement of regulatory authorities, the investigator/Study Center must agree to keep all relevant records, including all subjects' ID number (with sufficient information linked to the records, such as the CRF and medical record), all original signed informed consent forms, all copies of the CRFs, safety reports, source records, detailed treatment records, and relevant communication documents (such as letters, minutes, and telephone reports). The investigator/Study Center should keep the records according to the related specifications.

Study documents should be kept by the Study Center for 5 years after completion of the clinical trial.

10 Data Processing and Intellectual Property

10.1 Data Processing

After the completion of the clinical trial, data will be processed by the data statistics company authorized by the sponsor. Inconsistency in the documents will be discovered via data verification. Any inconsistency in the documents should be clarified by the investigator. The database will be locked after the data is verified. No access is allowed without authorization. Following database locking, the statistician unblinds the data after applying with the sponsor and PI and obtaining

agreement from all the three parties. To ensure the safety of the data, irrelevant personnel cannot access and modify data. Data should be backed up. Any changes to the data should be signed by the principal investigator, statistician, and data manager before it can be changed.

11 Statistical Analysis

11.1 Determination of Sample Size

The SIMON's two-stage design will be used. It is expected that the ORR of chemotherapy in patients with advanced TNBC who have failed multiline therapies is about 26%[14]. Our preliminary trial found that the ORR of patients with advanced TNBC received 1-3 lines treatment at the advanced stage treated with apatinib combined with camrelizumab was 43%[11]. In this study, we have expected that the ORR of heavily treated therapy-experienced advanced TNBC can be increased to 46% after camrelizumab combined with eribulin plus apatinib. According to a two-sided test level of 0.05 and power = 0.80, 14 patients are required to be enrolled in stage I. If less than 5 patients have achieved ORR in stage I, the trial will be terminated and the result of this trial is negative. If 5 or more patients have achieved ORR in stage I, a total of 32 patients will be enrolled into stage II, i.e., we will totally enroll 46 patients. If a total of > 16 patients have achieved ORR in the two stages, the result of this trial is positive.

11.2 Analysis Sets

Full Analysis Set (FAS)

All enrolled subjects who have received at least one dose of the investigational drug.

Per-Protocol Set (PPS)

All enrolled subjects who have received at least one dose of the investigational drug without significant protocol deviation.

Safety Analysis Set (SAS)

All enrolled subjects who have received at least one dose of the investigational drug and have safety records after administration. This data set is used for safety analysis.

11.3 Analysis of the Primary Endpoint

The primary endpoint is ORR, defined as a complete response (CR) or partial response (PR), as determined by investigator assessment and confirmed by repeat assessment ≥4 weeks after initial documentation. ORR will be estimated and 95% CI for the estimated rate will be constructed using the Clopper-Pearson method. Analysis of ORR will performed in both Full Analysis Set (FAS) population and Per-Protocol Set (PPS).

11.4 Analysis of the Secondary Endpoints

The secondary endpoints are defined as follow:

Disease Control Rate (DCR) is defined as the percentage of patients who have achieved CR, PR and stable disease (SD) to the therapy.

Duration of response (DoR) is the time from response (R) to progression/death (P/D).

Progression-Free-Survival (PFS) is defined as the time from date of first dose of study drug to the date of disease progression or death, whichever occurred first. Second primary cancer was not counted as a PFS endpoint.

One-year Overall survival (One-year OS) is defined as the percentage of subjects who are alive after one year.

Clinical benefit rate (CBR) is defined as the proportion of patients who achieved an objective response or had stable disease as their best response for 24 weeks or more.

Time to response (TTR) is defined as the time elapsed between date of first dosing, and the day CR or PR is first achieved.

The point estimates of secondary efficacy endpoints such as DCR, and CBR with the corresponding two-sided 95% confidence intervals (CI) will be calculated using the Clopper-Pearson method. For survival, the median duration of PFS, DoR, PFS, One-year OS, TTR and their 95% confidence intervals representing the entire population are estimated using the Kaplan-Meier method. In addition, survival plots are plotted.

Statistical analysis will be carried out after the completion of the study, and the report will be submitted.

11.5 General Analysis

Trial results are mainly analyzed using descriptive statistics. Measurement data will be

summarized using the mean, standard deviation, median, maximum, minimum. Enumeration data and ranked data will be summarized using the frequency (proportion), percentage, and confidence interval.

Statistical analysis will be performed using the STATA (version 12.0; Stata Co., College Station, TX). All statistical tests were two-sided, and statistical significance was defined as $P < 0.05$.

11.6 Baseline Characteristics

Quantitative data such as age and disease-free interval[1] (DFI) are summarized using the mean, standard deviation, median, maximum, and minimum. Qualitative data such as PD-L1 expression status, prior therapy lines in advanced setting, and ECOG PS score are summarized using frequency and percentage.

11.7 Subgroup Analysis

At the time of efficacy analysis, we will conduct the subgroup analysis by estimating the hazard ratio with 95%CI and the test interaction, the association between ORR and subgroups will be assessed by using χ^2 test or Fisher's exact test. We will estimate the PFS in subgroups with Kaplan-Meier method, and will compare them using log-rank tests if applicable, among subgroups with two-sided (*P*-values) for the following items:

- CPS score (<1 vs. ≥1; <10 vs.≥10)
- Sites of metastatic disease (<3 vs. ≥3)
- Liver metastasis (Yes vs. No)
- Treatment lines at the stage of recurrence or metastasis (1-2 vs. ＞2)
- Previous usage of checkpoint inhibitors (Yes vs. No)
- DFI (de novo vs. DFI≥1 year vs. DFI < 1 year)

11.8 Safety Evaluation

Safety data will be summarized based on the Safety Analysis Set. An adverse event refers to any untoward medical event that occurs in a subject of clinical study after signing the informed consent form. Any adverse medical events that occur from starting drug treatment until 30 days after the last dose should be followed-up until the adverse events are resolved or stabilized. SAE and

[1] DFI is defined as the period of time between the end of adjuvant treatment after surgery and the first sign of tumor recurrence.

irAE observed within 90 days after the last dose of camrelizumab should be followed up. If the subjects start a new anti-tumor treatment, they should be followed-up until they start the tumor treatment (if the tumor treatment is started within 30 days after the last dose, the subjects should be followed-up until at least 30 days after the last dose; if the tumor treatment is started more than 30 days after the last dose, the subjects should be followed-up until they start the new tumor treatment). Regardless of whether or not considered as related to the study drugs, these adverse medical events are all judged as AEs.

All AEs, including serious adverse events (SAEs), will be summarized by NCI-CTCAE v4.03 grade:

Grade I: Mild, asymptomatic or mild symptoms; clinical or laboratory abnormalities only; treatment not indicated.

Grade II: Moderate, minimal, local or noninvasive intervention indicated; limited age-appropriate instrumental activities of daily living (refer to preparing meals, shopping for groceries and clothes, using the telephone, managing money, etc.).

Grade III: Severe or medically significant but not immediately life-threatening; hospitalization or prolongation of hospitalization indicated; disabling; limiting self-care ADL. Self-care ADL: refer to bathing, dressing and undressing, feeding self, using the toilet, taking medications, and not bedridden.

Grade IV: Life-threatening consequences; urgent intervention indicated.

Grade V: Death related to AEs.

The clinical physician will comprehensively determine the relationship between the AEs and investigational drug and assess the possible relationship as "definitely related, possibly related, unlikely related, not related, and not assessable". "Definitely related, possibly related, and not assessable" events are included as adverse drug reactions. The incidence of an AE is calculated with the sum of these three categories as the numerator and the number of subjects used for safety evaluation as the denominator.

Adverse event data will be listed by study site, dose, patient number, and study day. Multiple occurrences of the same event will be counted once at the maximum severity for each patient.

Descriptive statistical analysis is primarily used to analyze the adverse events, serious adverse events and adverse reactions in the study. For laboratory results, the cases in which the pre-treatment values are normal but the post-treatment values are abnormal will be described. The mean, standard deviation, median, minimum, and maximum of vital signs (blood pressure, heart rate, body temperature, and respiratory rate) before and after treatment.

The number and rate of laboratory measurements, ECG, and physical examination "changed from normal to abnormal" or "exacerbated", and abnormalities after the trial and their clinical explanations are presented by tabulation.

11.9 Other Analyses

In order to explore the relationship between immune-related biomarkers and efficacy of the combination therapy of camrelizumab, apatinib and eribulin, the following exploratory biomarker endpoints will be assessed when appropriate:

- PD-L1 and other exploratory biomarkers in tumor tissue
- Stromal TILs
- Immune biomarkers in blood

For biomarker analysis, PD-L1 expression will be measured using the FDA-cleared 22C3 assay on the Dako Link 48 platform following the manufacturer's instructions. The measure of expression level is the combined positive score (CPS), defined as the ratio of PD-L1-positive cells (tumor cells, lymphocytes, and macrophages) divided by the total number of tumor cells multiplied by 100. PD-L1 positivity is defined as a CPS score of 1 or greater. Stromal TILs will be evaluated in hematoxylin and eosin sections following the criteria proposed by the International TIL WG22. For other exploratory biomarkers in tumor tissue, high multiplexed imaging, multiple immunofluorescence and proteomics will be used.

Expression levels of PD-L1 in tumor, Stromal tumor-infiltrating lymphocytes (TILs) as well as immune biomarkers in blood will be analyzed using descriptive statistics. The Wilcoxon rank-sum or Kruskal-Wallist test will be performed to assess the associations between baseline biomarkers. The log-rank test and chi-square test is used to assess the associations between distinct biomarkers and clinical outcomes.

12 End of Study and Expected Schedule

12.1 End of Study

The study is completed at 6 months after the first dose of the subjects, and the primary and secondary endpoints of the study will be statistically analyzed.

All subjects will be followed up until the occurrence of an event that meets the criteria for discontinuation for the dosing of the last subject, and a supplemental analysis of the primary and secondary endpoints of the study will be performed after the follow-up.

After the end of the study, if the subjects continue to benefit from the study drug, they may continue to use it until the criteria for treatment discontinuation is met. In addition, the occurrence of SAEs are collected and recorded during drug administration and after the last dose according to the protocol.

12.2 Expected Study Schedule

February 2020 to December 2022.

13 Reference

1. Bauer KR, Brown M, Cress RD, Parise CA, Caggiano V. Descriptive analysis of estrogen receptor (ER)-negative, progesterone receptor (PR)-negative, and HER2-negative invasive breast cancer, the so-called triple-negative phenotype: a population-based study from the California cancer Registry. *Cancer* 2007; **109**(9): 1721-8.
2. Dent R, Trudeau M, Pritchard KI, et al. Triple-Negative Breast Cancer: Clinical Features and Patterns of Recurrence. *Clinical Cancer Research* 2007; **13**(15): 4429-34.
3. Breast Cancer, Version 1.2020, NCCN Clinical Practice Guidelines in Oncology. *2020 National Comprehensive Cancer Network* 2020.
4. McDermott DF, Sosman JA, Sznol M, et al. Atezolizumab, an Anti–Programmed Death-Ligand 1 Antibody, in Metastatic Renal Cell Carcinoma: Long-Term Safety, Clinical Activity, and Immune Correlates From a Phase Ia Study. *Journal of Clinical Oncology* 2016; **34**(8): 833-42.
5. Schmid P, Rugo HS, Adams S, et al. Atezolizumab plus nab-paclitaxel as first-line treatment for unresectable, locally advanced or metastatic triple-negative breast cancer (IMpassion130): updated efficacy results from a randomised, double-blind, placebo-controlled, phase 3 trial. *The Lancet Oncology* 2020; **21**(1): 44-59.
6. Alexandrescu DT, Ichim TE, Riordan NH, et al. Immunotherapy for Melanoma: Current Status and Perspectives. *J Immunother* 2010; **33**(6): 570-90.
7. Mizugaki H, Yamamoto N, Murakami H, et al. Phase I dose-finding study of monotherapy with atezolizumab, an engineered immunoglobulin monoclonal antibody targeting PD-L1, in Japanese patients with advanced solid tumors. *Investigational New Drugs* 2016; **34**(5): 596-603.
8. Xu J, Zhang Y, Jia R, et al. Anti-PD-1 Antibody SHR-1210 Combined with Apatinib for Advanced Hepatocellular

Carcinoma, Gastric, or Esophagogastric Junction Cancer: An Open-label, Dose Escalation and Expansion Study. *Clin Cancer Res* 2019; **25**(2): 515-23.

9. Zhou C, Gao G, Wang YN, et al. Efficacy of PD-1 monoclonal antibody SHR-1210 plus apatinib in patients with advanced nonsquamous NSCLC with wild‐type EGFR and ALK. *Journal of Clinical Oncology* 2019; **37**(15_suppl): 9112-.

10. Li Q, Wang Y, Jia W, et al. Low-dose anti-angiogenic therapy sensitizes breast cancer to PD-1 blockade. *Clin Cancer Res* 2019.

11. Liu J, Jiang Z, Li Q, Li Y, Liu Q, Song E. Efficacy and safety of anti-PD-1 antibody SHR-1210 combined with apatinib in patients with advanced triple-negative breast cancer. *Journal of Clinical Oncology* 2019; **37**(15_suppl): 1066-.

12. Cortes J, O'Shaughnessy J, Loesch D, et al. Eribulin monotherapy versus treatment of physician's choice in patients with metastatic breast cancer (EMBRACE): a phase 3 open-label randomised study. *The Lancet* 2011; **377**(9769): 914-23.

13. Tolaney SM KK, Kaklamani V, et al. Phase 1b/2 study to evaluate eribulin mesylate in combination with pembrolizumab in patients with metastatic triple-negative breast cancer. *Presented at: 2017 San Antonio Breast Cancer Symposium; December 5-9, 2017; San Antonio, TX Abstract PD6-13.*

14. Yuan P, Hu X, Sun T, et al. Eribulin mesilate versus vinorelbine in women with locally recurrent or metastatic breast cancer: A randomised clinical trial. *Eur J Cancer* 2019; **112**: 57-65.

Appendix 1. Prohibited Traditional Chinese Medicine (TCM)

Huatan Huisheng Tablet

Brucea Javanica Oil Soft Capsule

Mandarin Melon Berry Syrup

Cantharidin

Cinobufotalin

Bufotoxin

Kang'ai Injection

Kanglaite

Zhongjiefeng Injection

Aidi Injection

Awei Huapi Ointment

Kangaiping Pill

Fukang Capsule

Xiaoaiping

Pingxiao Capsule

Pingxiao Tablet

Shendan Sanjie Capsule

Ankangxin Capsule

Boshengaining

Zedoary Turmeric Oil and Glucose Injection

Kanglixin Capsule

Cidan Capsule

Appendix 2. Performance Status Criteria (ECOG)

(Eastern Cooperative Oncology Group)

Score	Description
0	Asymptomatic, fully active, able to carry on all performance without restriction.
1	Symptomatic, restricted in physically strenuous activity but ambulatory and able to carry out work of a light or sedentary nature, e.g., light house work, office work.
2	Symptomatic, ambulatory and capable of all selfcare but unable to carry out any work activities; up and about more than 50% of waking hours (confined to bed < 50% of waking hours).
3	Symptomatic, capable of only limited selfcare; confined to bed or chair more than 50% of waking hours, but not totally confined to bed.
4	Completely disabled; cannot carry on any selfcare; totally confined to bed or chair.
5	Death

Appendix 3. Calculation of Creatinine Clearance

Creatinine Clearance Calculation Using the Cockcroft-Gault Formula

Please choose the appropriate formula corresponding to the unit of serum creatinine test:

If the unit of serum concentration of creatinine is mg/dL

Creatinine clearance in males (mL/min) = $\frac{(140 - \text{Year}) \times (\text{Weight})}{72 \times \text{Serum creatinine}}$

Creatinine clearance in females (mL/min) = $\frac{0.85 \times (140 - \text{Year}) \times (\text{Weight})}{72 \times \text{Serum creatinine}}$

If the unit of serum concentration of creatinine is μmol/L

Creatinine clearance in males (mL/min) = $\frac{(140 - \text{Year}) \times (\text{Weight})}{0.81 \times \text{Serum creatinine}}$

Creatinine clearance in females (mL/min) = $\frac{0.85 \times (140 - \text{Year}) \times (\text{Weight})}{0.81 \times \text{Serum creatinine}}$

Note: The unit of age is years old, and the unit of body weight is kilogram (kg).

Appendix 4. Management Principles for irAEs

1. Management Principles for Gastrointestinal Aes

Non-inflammatory causes of disease should be excluded. Opioids/anesthetics may mask the symptoms of perforation. Do not use infliximab in the case of perforation/sepsis.

Grade of Diarrhoea /Colitis (NCI CTCAE V4)	Treatment	Follow-up
**Grade 1**<br>Diarrhoea: Increase of < 4 stools per day over baseline<br>Colitis: Asymptomatic	• Continue I-O treatment according to the study protocol<br>• Symptomatic treatment	• Closely monitor aggravated symptoms.<br>• Educate patients to report aggravated symptoms immediately<br>If it is aggravated:<br>• Provide treatment according to the method for Grade 2 or Grade 3/4 situations
**Grade 2**<br>Diarrhoea: Increase of 4-6 stools per day over baseline; intravenous infusion of <24 h is required; daily living is not affected<br>Colitis: Abdominal pain; hematochezia	• Delay I-O treatment according to the study protocol<br>• Symptomatic treatment	If it is improved to Grade 1:<br>• Resume I-O treatment according to the study protocolIf it lasts for >5-7 days or relapses:<br>• Intravenous administration of 0.5-1.0 mg/kg/day methylprednisolone or an equivalent orally administrated dose<br>• When the symptoms improve to grade 1, reduce the dose of steroids for at least 1 month and consider prophylactic antibiotics to prevent opportunistic infections, then resume I-O treatment according to the study protocol<br>If the symptoms are aggravated or persisted after >3-5 days of oral administration of steroids:<br>• Provide treatment according to the method for Grade 3/4 situations
**Grade 3-4**<br>Diarrhoea (Grade 3): Increase of ≥ 7 stools per day over baseline; fecal incontinence; intravenous infusion of ≥24 h is required; daily living is affected<br>Colitis (Grade 3): Severe abdominal pain, indications for medical intervention, peritoneal signs<br>Grade 4: Life-threatening, perforation	• Terminate I-O treatment according to the study protocol<br>• Intravenous administration of 1.0-2.0 mg/kg/day methylprednisolone or an equivalent dose via intravenous injection<br>• Add prophylactic antibiotics to prevent opportunistic infections<br>• Consider lower GI endoscopy	If symptoms improve:<br>• Continue steroid treatment until the symptoms return to grade 1, then gradually reduce the dose of steroids for at least 1 month<br>If it lasts for >3-5 days or relapses after improvement:<br>• Add 5 mg/kg infliximab (if there are no contraindications). Note: Do not use infliximab in the case of perforation/sepsis.

Once a patient given intravenous injections of steroids shows a sustained clinical improvement, the patient can switch to an equivalent dose of oral corticosteroids (e.g., prednisone) by the time or before the dose of steroid injections starts to be gradually reduced. When switching to oral corticosteroids with an equivalent dose, it should be considered that the bioavailability of oral corticosteroids is relatively low.

2. Management Principles for Pulmonary AEs

Non-inflammatory causes of disease should be excluded. If it is due to a non-inflammatory cause, a symptomatic treatment should be given while the I-O therapy should be continued.

maging evaluation and consultations with the respiratory department should be performed.

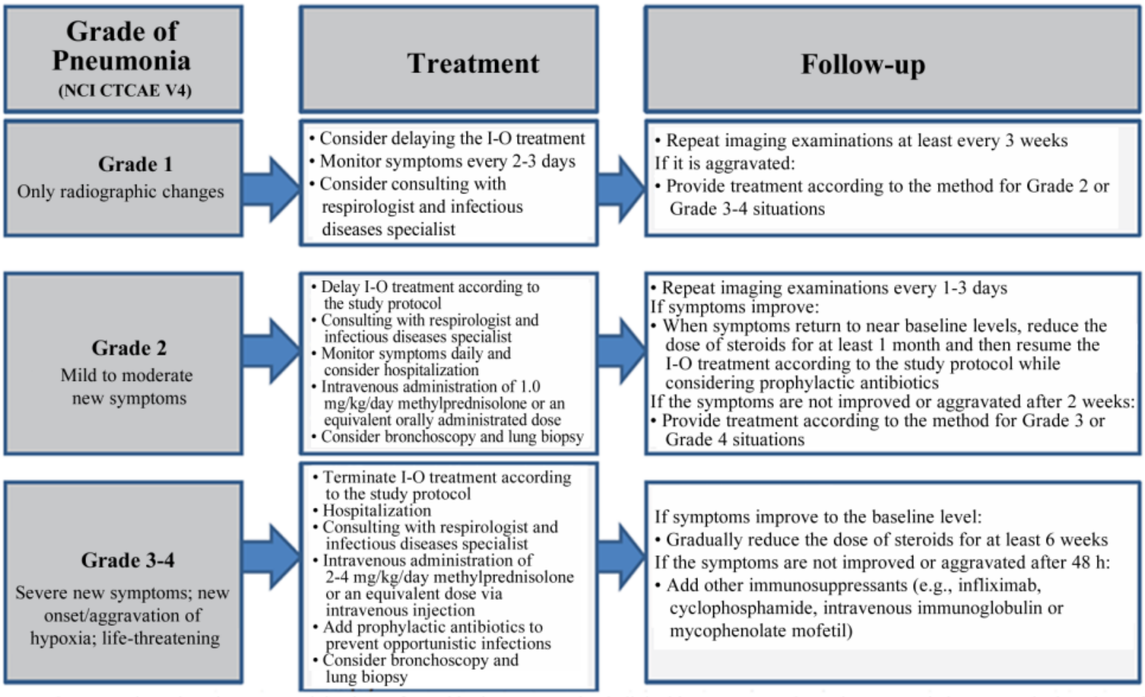

Once a patient given intravenous injections of steroids shows a sustained clinical improvement, the patient can switch to an equivalent dose of oral corticosteroids (e.g., prednisone) by the time or before the dose of steroid injections starts to be gradually reduced. When switching to oral corticosteroids with an equivalent dose, it should be considered that the bioavailability of oral corticosteroids is relatively low.

3. Management Principles for Hepatic AEs

Non-inflammatory causes of disease should be excluded. If it is due to a non-inflammatory cause, a symptomatic treatment should be given while the I-O therapy should be continued. Consider imaging examinations to rule out obstruction/tumor progression.

Grade of Liver Function Test Increased (NCI CTCAE V4)	Treatment	Follow-up
**Grade 1**<br>AST or ALT > ULN-3.0 × ULN and/or T.bili > ULN-1.5 × ULN	• Continue I-O treatment according to the study protocol	• Continue liver function monitoring according to the study protocol<br>If it is aggravated:<br>• Provide treatment according to the method for Grade 2 or Grade 3/4 situations
**Grade 2**<br>AST or ALT > 3.0 to ≤ 5 × ULN and/or T.bili > 1.5 to ≤ 3 × ULN	• Delay I-O treatment according to the study protocol<br>• Increase the monitoring frequency to once every 3 days	If it returns to the baseline level:<br>• Resume routine monitoring and resume I-O treatment according to the study protocol<br>If the increase lasts for > 5-7 days or becomes aggravated:<br>• Intravenous administration of 0.5-1 mg/kg/day methylprednisolone or an equivalent orally administrated dose. If LFT returns to grade 1 or baseline, reduce the dose of steroids for at least 1 month and consider prophylactic antibiotics to prevent opportunistic infections, and then resume I-O treatment according to the study protocol
**Grade 3-4**<br>AST or ALT > 5 × ULN and/or T.bili > 3 × ULN	• Terminate I-O treatment*<br>• Increase the monitoring frequency to once every 1-2 days<br>• Intravenous administration of 1.0-2.0 mg/kg/day methylprednisolone or an equivalent dose via intravenous injection**<br>• Add prophylactic antibiotics to prevent opportunistic infections<br>• Consultation with the gastroenterology department	If it returns to Grade 2:<br>• Reduce the dose of steroids for at least 1 month<br>If there is no improvement within >3-5 days, or if the symptoms are aggravated or deteriorated:<br>• Add 1 g of mycophenolate mofetil, b.i.d<br>• If the symptoms are not relieved within 3-5 days, consider other immunosuppressants based on local guidelines

Once a patient given intravenous injections of steroids shows a sustained clinical improvement, the patient can switch to an equivalent dose of oral corticosteroids (e.g., prednisone) by the time or before the dose of steroid injections starts to be gradually reduced. When switching to oral corticosteroids with an equivalent dose, it should be considered that the bioavailability of oral corticosteroids is relatively low.

*If AST/ALT ≤ 8 × ULN and T.bili ≤ 5 × ULN, the I-O treatment can be delayed rather than discontinued.

**For grade 4 hepatitis, the recommended starting dose of methylprednisolone intravenous injection is 2 mg/kg/day.

4. Management Principles for Endocrine AEs

Non-inflammatory causes of disease should be excluded. If it is due to a non-inflammatory cause, a symptomatic treatment should be given while the I-O therapy should be continued.

Visual field tests, endocrinology consultation and imaging examinations are considered

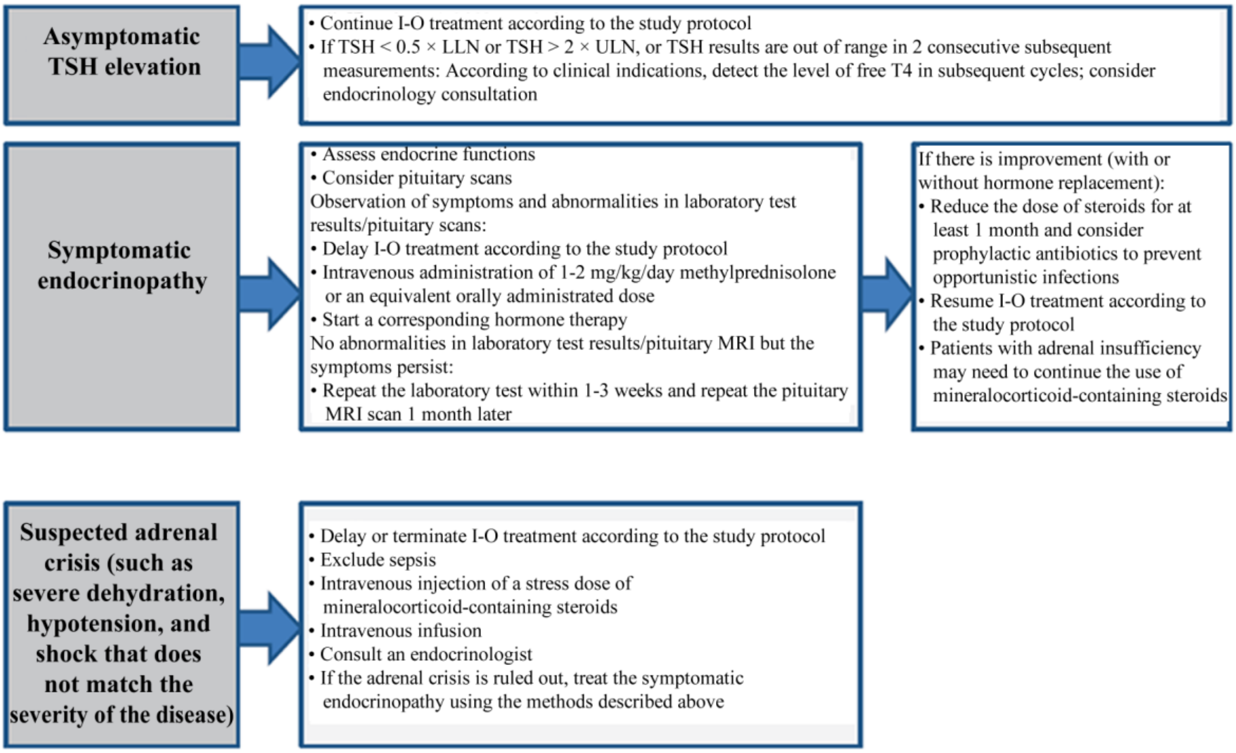

Once a patient given intravenous injections of steroids shows a sustained clinical improvement, the patient can switch to an equivalent dose of oral corticosteroids (e.g., prednisone) by the time or before the dose of steroid injections starts to be gradually reduced. When switching to oral corticosteroids with an equivalent dose in the lungs and liver, it should be considered that the bioavailability of oral corticosteroids is relatively low.

5. Management Principles for Skin AEs

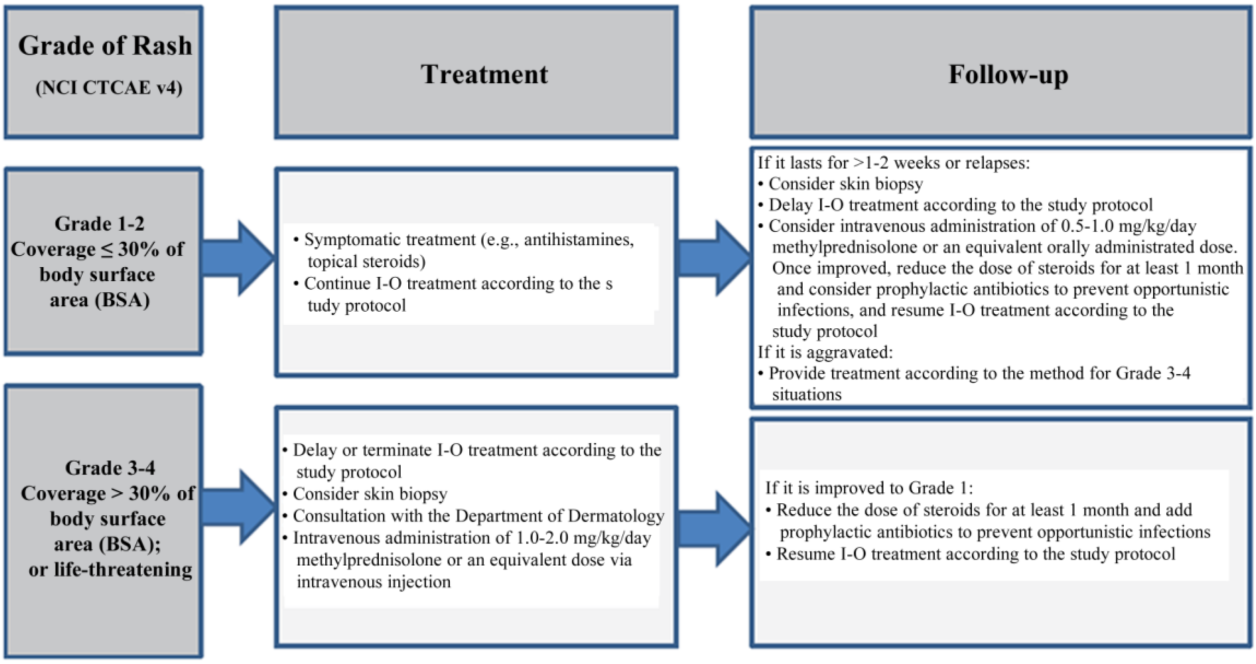

Once a patient given intravenous injections of steroids shows a sustained clinical improvement, the patient can switch to an equivalent dose of oral corticosteroids (e.g., prednisone) by the time or before the dose of steroid injections starts to be gradually reduced. When switching to oral corticosteroids with an equivalent dose in the lungs and liver, it should be considered that the bioavailability of oral corticosteroids is relatively low.

(***Weber JS***, *Postow M, Lao CD, Schadendorf D. Management of Adverse Events Following Treatment With Anti-Programmed Death-1 Agents. Oncologist. 2016 Jul 8: 2016-0055.*)

Appendix 5. Response Evaluation Criteria in Solid Tumors

New Response Evaluation Criteria in Solid Tumors: Revised RECIST Version 1.1 (Excerpt)

Note: This Appendix is translated internally and is for reference only. Please refer to the English version during practice.

1. Background

Omitted

2. Objectives

Omitted

3. Measurability of tumor at baseline

3.1 Definitions

At baseline, tumor lesions/lymph nodes will be categorized measurable or non-measurable as follows:

3.1.1 Measurable Lesions

Tumor lesions: Must be accurately measured in at least one dimension (longest diameter is to be recorded) with a minimum size of:

- 10 mm by CT scan (CT scan slice thickness no greater than 5 mm)
- 10 mm caliper measurement by clinical exam (lesions which cannot be accurately measured with calipers should be recorded as non-measurable)
- 20 mm by chest X-ray
- Malignant lymph nodes: To be considered pathologically enlarged and measurable, a lymph node must be ≥ 15 mm in short axis when assessed by CT scan (CT scan slice thickness recommended to be no greater than 5 mm). At baseline and in follow-up, only the short axis will be measured and followed.

3.1.2 Non-Measurable Lesions

All other lesions, including small lesions (longest diameter < 10 mm or pathological lymph nodes with ≥ 10 to < 15 mm short axis) as well as truly non-measurable lesions. Lesions considered truly non-measurable include: leptomeningeal disease, ascites, pleural or pericardial effusion, inflammatory breast disease, lymphangitic involvement of skin or lung, abdominal

masses/abdominal organomegaly identified by physical exam that is not measurable by reproducible imaging techniques, and cystic lesion.

3.1.3 Special Considerations Regarding Lesion Measurability

Bone lesions, cystic lesions, and lesions previously treated with local therapy require particular comment:

Bone lesions:

- Bone scan, PET scan or plain films are not considered adequate imaging techniques to measure bone lesions. However, these techniques can be used to confirm the presence or disappearance of bone lesions;
- Lytic lesions or mixed lytic-blastic lesions, with identifiable soft tissue components, that can be evaluated by tomography techniques such as CT or MRI can be considered as measurable lesions if the soft tissue component meets the definition of measurability described above;
- Blastic bone lesions are non-measurable.

Cystic lesions:

- Lesions that meet the criteria for radiographically defined simple cysts should not be considered as malignant lesions (neither measurable nor non-measurable) since they are, by definition, simple cysts;
- Cystic lesions thought to represent cystic metastases can be considered as measurable lesions, if they meet the definition of measurability described above. However, if non-cystic lesions are present in the same patient, these are preferred for selection as target lesions.

Lesions with prior local treatment:

- Tumor lesions situated in a previously irradiated area, or in an area subjected to other loco-regional therapy, are usually considered non-measurable unless there has been demonstrated progression in the lesion. Study protocols should detail the conditions under which such lesions would be considered measurable.

3.2 Methods of Measurement

3.2.1 Measurement of Lesions

All measurements should be recorded in metric notation, using calipers if clinically assessed. All baseline evaluations should be performed as close as possible to the treatment start and never more than 28 days (4 weeks) before the beginning of the treatment.

3.2.2 Method of Assessment

The same method and the same technique should be used to assess lesions at baseline and during follow-up. Imaging based evaluation should always be done rather than clinical examination unless the lesion(s) being followed cannot be imaged but are assessable by clinical exam.

Clinical lesions: Clinical lesions will only be considered measurable when they are superficial and ≥ 10 mm diameter as assessed using calipers (e.g., skin nodules). For the case of skin lesions, documentation by color photography including a ruler to estimate the size of the lesion is suggested. When lesions can be evaluated by both clinical exam and imaging, imaging evaluation should be undertaken since it is more objective and may also be reviewed at the end of the study.

Chest X-ray: Chest CT is preferred over chest X-ray, especially when tumor progression is an important clinical endpoint, since CT is more sensitive, particularly in identifying new lesions. Chest X-ray is only applicable when the measured lesion boundary is clear and the lungs are well ventilated.

CT, MRI: CT is currently the best available and reproducible method for efficacy evaluation. This guideline has defined measurability of lesions on CT scan based on the assumption that ≤ CT slice thickness is ≤ 5 mm. When CT scans have slice thickness greater than 5 mm, the minimum size for a measurable lesion should be twice the slice thickness. MRI is also acceptable in certain situations (e.g. for body scans).

Ultrasound: Ultrasound should not be used as a method to measure lesion size. Ultrasound examinations are operation-dependent, and cannot be reproduced at a later date. It cannot be guaranteed that the same technique and measurements will be taken from one assessment to the next. If new lesions are identified by ultrasound in the course of the study, confirmation by CT or MRI is advised. If there is concern about radiation exposure at CT, MRI may be used instead.

Endoscopy, laparoscopy: The utilization of these techniques for objective tumor evaluation is not advised. However, they can be useful to confirm CR when biopsies are obtained, or to determine relapse in trials where recurrence following CR or surgical excision is an endpoint.

Tumor Markers: Tumor markers alone cannot be used to assess objective tumor response. If markers are initially above the upper normal limit, however, they must normalize for a patient to be considered in complete response. Because tumor markers are disease specific, instructions for their measurement should be incorporated into protocols on a disease specific basis. Specific guidelines for both CA-125 response (in recurrent ovarian cancer) and PSA response (in recurrent prostate cancer), have been published. In addition, the Gynecologic Cancer Intergroup has developed CA-125 progression criteria which are to be integrated with objective tumor assessment for use in first-line trials in ovarian cancer.

Cytology/histology: These techniques can be used to differentiate between PR and CR in rare cases if required by protocol (for example, residual lesions in tumor types such as germ cell neoplasm, where known residual benign neoplasm can remain). When effusions are known to be a potential adverse effect of treatment (e.g. with certain taxane compounds or angiogenesis inhibitors), the cytological confirmation of the neoplastic origin of any effusion that appears or worsens during treatment can be considered if the measurable tumor has met criteria for response or stable disease in order to differentiate between response (or stable disease) and progressive disease.

4 Tumor Response Assessment

4.1 Assessment of Overall Tumor Burden and Measurable Disease

To assess objective response or future progression, it is necessary to estimate the overall tumor burden at baseline and use this as a comparator for subsequent measurements. Only patients with measurable disease at baseline should be included in protocols where objective response is the primary endpoint. Measurable disease is defined by the presence of at least one measurable lesion.

In trials where the primary endpoint is tumor progression (either time to progression or proportion with progression at a fixed date), the protocol must specify if enrollment is restricted to

those with measurable disease or whether patients having non-measurable disease only are also eligible.

4.2 Baseline Documentation of "Target" and "Non-Target" Lesions

When more than one measurable lesion is present at baseline, all lesions up to a maximum of five lesions total (and a maximum of two lesions per organ), representative of all involved organs should be identified as target lesions and will be recorded and measured at baseline (this means in instances where patients have only one or two organ sites involved, a maximum of two and four lesions respectively will be recorded).

Target lesions should be selected on the basis of their size (lesions with the longest diameter), be representative of all involved organs, but in addition should be those that lend themselves to reproducible repeated measurements. It may be the case that, on occasion, the largest lesion does not lend itself to reproducible measurement in which circumstance the next largest lesion which can be measured reproducibly should be selected.

Lymph nodes merit special mention since they are normal tissues which may be visible by imaging even if not involved by tumor metastasis. Pathological nodes which are defined as measurable and may be identified as target lesions must meet the criterion of a short axis of ≥ 15 mm by CT scan. Only the short axis of these nodes needs to be measured at baseline. The short axis of the node is the diameter normally used by radiologists to judge if a node is involved by tumor metastasis. Nodule size is normally reported as two dimensions in the plane in which the image is obtained (for CT scan this is almost always the axial plane; for MRI the plane of acquisition may be axial, saggital or coronal). The smaller of these measures is the short axis. For example, an abdominal node which is reported as being 20 mm × 30 mm has a short axis of 20 mm and qualifies as a malignant, measurable node. In this example, 20 mm should be recorded as the node measurement. Nodes with short axis ≥ 10 mm but < 15 mm) should be considered non-target lesions. Nodes that have a short axis < 10 mm are considered non-pathological and should not be recorded or followed.

A sum of the diameters (longest for non-nodal lesions, short axis for nodal lesions) for all target lesions will be calculated and reported as the baseline sum diameters. If lymph nodes are to

be included in the sum, then as noted above, only the short axis is added into the sum. The baseline sum diameters will be used as reference.

All other lesions including pathological lymph nodes should be identified as non-target lesions and should also be recorded at baseline. Measurements are not required and these lesions should be followed as "present", "absent", or in rare cases "unequivocal progression". It is possible to record multiple nontarget lesions involving the same organ as a single item on the case record form (e.g., "multiple enlarged pelvic lymph nodes" or "multiple liver metastases").

4.3 Response Criteria

4.3.1 Evaluation of Target Lesions

Complete response (CR): Disappearance of all target lesions. Any pathological lymph nodes (whether target or non-target) must have reduction in short axis to < 10 mm.

Partial Response (PR): At least a 30% decrease in the sum of diameters of target lesions, compared to baseline.

Progressive Disease (PD): At least a 20% increase in the sum of diameters of target lesions, taking as reference the smallest sum on study (this includes the baseline sum if that is the smallest on study). In addition, the sum must also demonstrate an absolute increase of at least 5mm (the appearance of one or more new lesions is also considered disease progression).

Stable Disease (SD): Neither sufficient shrinkage to qualify for PR nor sufficient increase to qualify for PD, taking as reference the smallest sum diameters while on study.

4.3.2 Special Notes on the Assessment of Target Lesions

Lymph nodes: Lymph nodes identified as target lesions should always have the actual short axis measurement recorded (measured in the same anatomical plane as the baseline examination), even if the nodes regress to below 10 mm on study. This means that when lymph nodes are included as target lesions, the sum of lesions may not be zero even if complete response criteria are met, since a normal lymph nodule is defined as having a short axis of < 10 mm. Case report forms or other data collection methods may therefore be designed to have target nodal lesions recorded in a separate section where, in order to qualify for CR, each nodule must achieve a short axis of < 10

mm. For PR, SD and PD, the actual short axis measurement of the nodes is to be included in the sum of target lesions.

Target lesions that become too small to measure: While on study, all lesions (nodal and non-nodal) recorded at baseline should have their actual measurements recorded at each subsequent evaluation, even when very small (e.g. 2 mm). However, sometimes lesions or lymph nodes which are recorded as target lesions at baseline become so faint on CT scan that the radiologist may not feel comfortable assigning an exact measure and may report them as being "too small to measure". When this occurs it is important that a value be recorded on the case report form. If it is the opinion of the radiologist that the lesion has likely disappeared, the measurement should be recorded as 0 mm. If the lesion is believed to be present and is faintly seen but too small to measure, a default value of 5 mm could be assigned. (Note: It is less likely that this rule will be used for lymph nodes since they usually have a definable size when normal and are frequently surrounded by adipose tissues as in the retroperitoneum; however, if a lymph nodule is believed to be present and is faintly seen but too small to measure, a default value of 5 mm could be assigned in this circumstance as well). This default value is derived from the 5 mm CT slice thickness (but should not be changed with varying CT slice thickness). The measurement of these lesions is potentially non-reproducible, therefore providing this default value will prevent false evaluation based upon measurement error. To reiterate, however, if the radiologist is able to provide an actual measure, that should be recorded, even if it is below 5 mm.

Lesions that split or coalesce: When non-nodal lesions fragmented, the longest diameters of the fragmented portions should be added together to calculate the target lesion sum. Similarly, as lesions coalesce, a plane between them may be maintained that would aid in obtaining maximal diameter measurements of each individual lesion. If the lesions have truly coalesced such that they are no longer separable, the vector of the longest diameter in this instance should be the maximal longest diameter for the coalesced lesion.

4.3.3 Evaluation of Non-Target Lesions

This section provides the definitions of the criteria used to determine the tumor response for the group of non-target lesions. While some non-target lesions may actually be measurable, they

need not be measured and instead should be assessed only qualitatively at the time points specified in the protocol.

Complete Response (CR): Disappearance of all non-target lesions and normalization of tumor marker level. All lymph nodes must be non-pathological in size (< 10 mm short axis).

Non-CR/Non-PD: Persistence of one or more non-target lesion(s) and/or maintenance of tumor marker level above the normal limits.

Progressive Disease (PD): Unequivocal progression of existing non-target lesions. Note: the appearance of one or more new lesions is also considered disease progression.

4.3.4 Special Notes on Assessment of Progression of Non-Target Disease

The concept of progression of non-target disease requires additional explanation as follows: When the patient also has measurable disease: In this setting, to achieve ‘unequivocal progression’ on the basis of the non-target disease, there must be an overall level of substantial worsening in non-target disease such that, even in presence of SD or PR in target disease, the overall tumor burden has increased sufficiently to merit discontinuation of therapy. A modest increase in the size of one or more non-target lesions is usually not sufficient to quality for unequivocal progression status. The designation of overall progression solely on the basis of change in non-target disease in the face of SD or PR of target disease will therefore be extremely rare.

When the patient has only non-measurable disease: This circumstance arises in some phase III trials when it is not a criterion of study inclusion to have measurable disease. The same general concepts apply here as noted above, however, in this instance there is no measurable disease assessment. Because worsening in non-target disease cannot be easily quantified (by definition: if all lesions are truly non-measurable), a useful test that can be applied when assessing patients for unequivocal progression is to consider if the increase in overall disease burden based on the change in non-measurable disease is comparable in magnitude to the increase that would be required to declare PD for measurable disease. For example, an increase in tumor burden representing an additional 73% increase in volume (which is equivalent to a 20% increase diameter in a measurable lesion). Examples include an increase in a peritoneal effusion from "trace "to "large", an increase in lymphangitic disease from "localized" to "widespread", or may be

described in protocols as "sufficient to require a change in treatment". Examples include an increase in a pleural effusion from trace to large, an increase in lymphangitic disease from localized to widespread, or may be described in protocols as "sufficient to require a change in therapy". If unequivocal progression is seen, the patient should be considered to have had overall PD at that point. While it would be ideal to have objective criteria to apply to non-measurable disease, the very nature of that disease makes it impossible to do so, therefore the increase must be substantial.

4.3.5 New Lesions

The appearance of new malignant lesions denotes disease progression; therefore, some comments on detection of new lesions are important. There are no specific criteria for the identification of new radiographic lesions; however, the finding of a new lesion should be unequivocal: For example, it should not be attributable to differences in scanning technique, change in imaging modality, or findings thought to represent something other than tumor (for example, some new bone lesions that may be simply healing, or re-occurrence of pre-existing lesions). This is particularly important when the patient's baseline lesions show partial or complete response. For example, necrosis of a liver lesion may be reported on a CT scan report as a new cystic lesion, which it is not.

A lesion identified on a follow-up study that was not scanned at baseline is considered a new lesion and will indicate disease progression. An example of this is the patient who has visceral disease at baseline and while on study has a CT or MRI brain ordered which reveals metastases.

The patient’s brain metastases are considered to be evidence of PD even if he/she did not have brain imaging at baseline.

If a new lesion is equivocal, for example because of its small size, continued treatment and follow-up evaluation are required to clarify if it represents a truly new disease. If repeated scans confirm there is definitely a new lesion, then progression should be declared using the date of the initial identification.

While FDG-PET response assessments generally need additional study, it is sometimes reasonable to incorporate the use of FDG-PET scanning to complement CT scanning in assessment

of progression (particularly possible new disease). New lesions on the basis of FDG-PET imaging can be identified according to the following process:

Negative FDG-PET at baseline, with a positive FDG-PET at follow-up is a sign of PD based on a new lesion.

No FDG-PET at baseline and a positive FDG-PET at follow-up:

If the positive FDG-PET at follow-up corresponds to a new site of disease confirmed by CT, PD is confirmed.

If the positive FDG-PET at follow-up is not confirmed as a new site of disease on CT, additional follow-up CT scans are needed to determine if there is truly progression occurring at that site (if so, the date of PD will be the date of the initial abnormal FDG-PET scan).

If the positive FDG-PET at follow-up corresponds to a pre-existing site of disease on CT that is not progressing on the basis of the imaging examination, this is not PD.

4.4 Evaluation of Best Overall Response

The best overall response is the best response recorded from the start of the trial until the end of trial taking into account any necessary requirement for confirmation. On occasion a response may not be documented until after the end of treatment so protocols should be clear if post-treatment assessments are to be considered in the evaluation of best overall response.

Protocols must specify how any new treatment introduced before progression will affect best response evaluation. The patient's best overall response evaluation will depend on the findings of both target and non-target diseases and will also take into consideration the characteristics of new lesions. Furthermore, depending on the nature of the study and the protocol requirements, it may also require confirmatory measurement. Specifically, in non-randomized trials where response is the primary endpoint, confirmation of PR or CR is needed to determine either one is the best overall response.

4.4.1 Time Point Response

It is assumed that at each time point specified in protocol, a efficacy response occurs. Table 1 provides a summary of the overall response status calculation at each time point for patients who have measurable disease at baseline.

Table 1. Time point response: patients with target (+/– non-target) disease

Target Lesions	Non-Target Lesions	New Lesions	Overall Response
CR	CR	No	CR
CR	Non-CR/non-PD	No	PR
CR	Not evaluable	No	PR
PR	Non-PD or not all evaluated	No	PR
SD	Non-PD or not all evaluated	No	SD
Not all evaluated	Non-PD	No	NE
PD	Any	Yes or No	PD
Any	PD	Yes or No	PD
Any	Any	Yes	PD

CR = complete response, PR = partial response, SD = stable disease, PD = progressive disease, and NE = not evaluable.

When patients have non-measurable (therefore non-target) disease only, Table 2 is to be used.

Table 2. Time point response: patients with non-target disease only.

Non-Target Lesions	New Lesions	Overall Response
CR	No	CR
Non-CR/Non-PD	No	Non-CR/Non-PD[a]
Not all evaluated	No	Not evaluable
Unequivocal PD	Yes or No	PD
Any	Yes	PD

a. "Non-CR/non-PD" is preferred over "stable disease" for non-target disease since SD is increasingly used as endpoint for efficacy evaluation in some trials so to assign this category when no lesions can be measured.

4.4.2 Missing Evaluation and Not Evaluable Designation

When no imaging/measurement is done at all at a particular time point, the patient is not evaluable at that time point. If only a subset of lesion measurements are made at an evaluation, usually the case is also considered not evaluable at that time point, unless a convincing argument can be made that the contribution of the individual missing lesion(s) has/have no effect on the assigned time point response. This would be most likely to happen in the case of PD. For example, if a patient had a baseline sum of 50 mm with three measured lesions and only two lesions were assessed at subsequent follow-up, but those gave a sum of 80 mm, the patient will have achieved PD status, regardless of the contribution of the missing lesion.

4.4.3 Best Overall Response: All Time Points

The best overall response is determined once all the data for the patient is known.

Best response determination in trials where confirmation of complete or partial response is not required: Best response in these trials is defined as the best response across all time points (for example, a patient who has SD in evaluation at Cycle 1, PR at Cycle 2, and PD at the last cycle has a best overall response of PR). When SD is believed to be best response, it must also meet the protocol specified minimum time calculated from baseline. If the minimum time is not met when SD is otherwise the best overall response, the patient's best overall response depends on the subsequent assessments. For example, a patient who has SD at Cycle 1, PD at Cycle 2 and does not meet minimum duration for SD, will have a best overall response of PD. The same patient lost to follow-up after the first SD assessment would be considered not evaluable.

Best overall response determination in trials where confirmation of complete or partial response is required: Complete or partial responses may be claimed only if the criteria for each are met at a subsequent time point as specified in the protocol (generally 4 weeks later). In this circumstance, the best overall response can be interpreted as in Table 3.

Table 3. Best overall response when confirmation of CR and PR required.

Overall Response First Time Point	Overall Response Subsequent Time	Best Overall Response
CR	CR	CR
CR	PR	SD, PD or PR[a]
CR	SD	SD provided minimum criteria for SD duration met, otherwise, PD
CR	PD	SD provided minimum criteria for SD duration met, otherwise, PD
CR	NE	SD provided minimum criteria for SD duration met, otherwise, NE
PR	CR	PR
PR	PR	PR
PR	SD	SD
PR	PD	SD provided minimum criteria for SD duration met, otherwise, PD
PR	NE	SD provided minimum criteria for SD duration met, otherwise, NE
NE	NE	NE

CR = complete response, PR = partial response, SD = stable disease, PD = progressive disease, and NE = not evaluable.

a: If a CR is truly met at first time point, then any disease seen at a subsequent time point, even disease meeting PR criteria relative to baseline, makes the disease PD at that point (since disease must have

reappeared after CR). Best response would depend on whether minimum duration for SD was met. However, sometimes CR may be claimed when subsequent scans suggest small lesions were likely still present and in fact the patient had PR, not CR at the first time point. Under these circumstances, the original CR should be changed to PR and the best response is PR.

4.4.4 Special Notes on Response Evaluation

When nodal disease is included in the sum of target lesions and the nodes decrease to "normal" size (< 10 mm), they may still have a measurement reported on scans. This measurement should be recorded even though the nodules are normal in order not to overstate progression should it be based on increase in size of the nodes. As noted earlier, this means that patients with CR may not have "zero" recorded on the case report form (CRF).

In trials where confirmation of response is required, repeated "not evaluable" time point evaluations may complicate best response determination. The analysis plan for the trial must address how missing data/evaluations will be addressed in determination of response and progression. For example, in most trials it is reasonable to consider a patient with time point responses of PR-NE-PR as a confirmed response.

Patients with an overall deterioration of health status requiring discontinuation of treatment without objective evidence of disease progression at that time should be reported as symptomatic deterioration. Efforts should be made to evaluate objective progression even after discontinuation of treatment. Symptomatic deterioration is not a description of an objective response: it is a reason for discontinuation of treatment. The objective response status of such patients is to be determined by evaluation of target and non-target disease as shown in Tables 1-3.

Conditions that define early progression, early death and inevaluability are study specific and should be clearly described in each protocol (depending on treatment duration and treatment cycle).

In some circumstances it may be difficult to distinguish residual lesions from normal tissues.

When the evaluation of complete response depends upon this determination, it is recommended that the residual lesion be investigated before assigning a status of complete response. FDG-PET may be used to confirm a response to a CR in a manner similar to a biopsy in cases where a residual radiographic abnormality is thought to represent fibrosis or scarring. The use of FDG-PET in this circumstance should be prospectively described in the protocol and supported by disease specific

medical literature for the indication. However, it must be acknowledged that both approaches may lead to false positive CR due to limitations of FDG-PET and biopsy resolution/sensitivity.

For equivocal findings of progression (e.g. very small and uncertain new lesions; cystic changes or necrosis in existing lesions), treatment may continue until the next scheduled evaluation. If at the next scheduled evaluation, progression is confirmed, the date of progression should be the earlier date when progression was suspected.

4.5 Frequency of Tumor Re-Evaluation

Frequency of tumor re-evaluation during treatment should be protocol-specific and consistent with the type and schedule of treatment. However, in the Phase II trials where the beneficial effect of treatment is not known, follow-up every 6-8 weeks (timed to coincide with the end of a cycle) is reasonable. Interval adjustments could be justified in specific regimens or circumstances. The protocol should specify which organ sites are to be evaluated at baseline (usually those most likely to be involved with metastatic disease for the tumor type under study) and how often evaluations are repeated. Normally, all target and non-target sites are evaluated at each assessment. In selected circumstances certain non-target organs may be evaluated less frequently. For example, bone scans may need to be repeated only when CR is identified in target disease or when progression in bone is suspected.

After the treatment, the need for tumor re-evaluations depends on whether the trial has as made the response rate or the time to an event (progression/death) an endpoint. If "time to an event" (e.g., TTP/DFS[2] /PFS) is the main endpoint of the study, then routine scheduled re-evaluation of protocol specified sites of disease is warranted. In randomized comparative trials in particular, the scheduled assessments should be performed as identified on a calendar schedule (for example: every 6-8 weeks on treatment or every 3-4 months after treatment) and should not be affected by delays in therapy, drug holidays or any other events that might lead to imbalance in a treatment arm in the timing of disease assessment.

4.6 Confirmatory Measurement/Duration of Response

4.6.1 Confirmation

[2] Disease free survival

In non-randomized trials where response is the primary endpoint, confirmation of PR and CR is required to ensure responses identified are not the result of measurement error. This will also permit appropriate interpretation of results in the context of historical data where response has traditionally required confirmation in such trials. However, in all other circumstances, i.e., in randomized trials (phase II or III) or studies where stable disease or progression are the primary endpoints, confirmation of response is not required since it will not add value to the interpretation of trial results. However, elimination of the requirement for response confirmation may increase the importance of central review to protect against bias, in particular in studies which are not blinded.

In the case of SD, measurements must have met the SD criteria at least once after study entry at a minimum interval (in general not less than 6–8 weeks) that is defined in the study protocol.

4.6.2 Duration of Overall Response

The duration of overall response is measured from the time measurement criteria are first met for CR/PR (whichever is first recorded) until the first date that recurrent or progressive disease is objectively documented (taking as reference for progressive disease the smallest measurements recorded on study). The duration of overall complete response is measured from the time criteria are first met for CR until the first date that recurrent or progressive disease is truly documented.

4.6.3 Duration of Stable Disease

Stable disease is measured from the start of the treatment (in randomized trials, from date of randomization) until the criteria for progression are met, taking as reference the smallest sum on study (if the baseline sum is the smallest, this is the reference for calculation of PD). The clinical relevance of the duration of stable disease varies in different studies and diseases. If the proportion of patients achieving stable disease for a minimum period of time is an endpoint in a particular trial, the protocol should specify the minimal time interval required between two measurements for determination of stable disease.

Note: The duration of response and stable disease as well as the progression-free survival are influenced by the frequency of follow-up after baseline evaluation. It is not in the scope of this guideline to define a standard follow-up frequency. The frequency should take into account many

parameters including disease types and stages, treatment periodicity and standard practice. However, these limitations of the precision of the measured endpoint should be taken into account if comparisons between trials are to be made.

4.7 PFS/TTP

4.7.1 Phase II Clinical Trial

This guideline is focused primarily on the use of objective response as study endpoints for phase II trials. In some circumstances, response rate may not be the optimal method to assess the potential anticancer activity of new agents/regimens. In such cases, PFS/PPF at landmark time points might be considered appropriate alternatives to provide an initial signal of biologic effect of new agents. It is clear, however, that in an uncontrolled trial, these measures are subject to criticism since an apparently promising observation may be related to biological factors such as patient selection and not the impact of the intervention. Thus, phase II screening trials utilizing these endpoints are best designed with a randomized control. Exceptions may exist where the behavior patterns of certain cancers are so consistent (and usually consistently poor), that a non-randomized trial is justifiable. However, in these cases, it will be essential to document with care the basis for estimating the expected PFS or PPF[3] in the absence of a treatment effect.

[3] Proportion of progress-free

Appendix 6. Percent Bone Marrow in Human Skeleton

Percent Bone Marrow in the Adult Skeleton

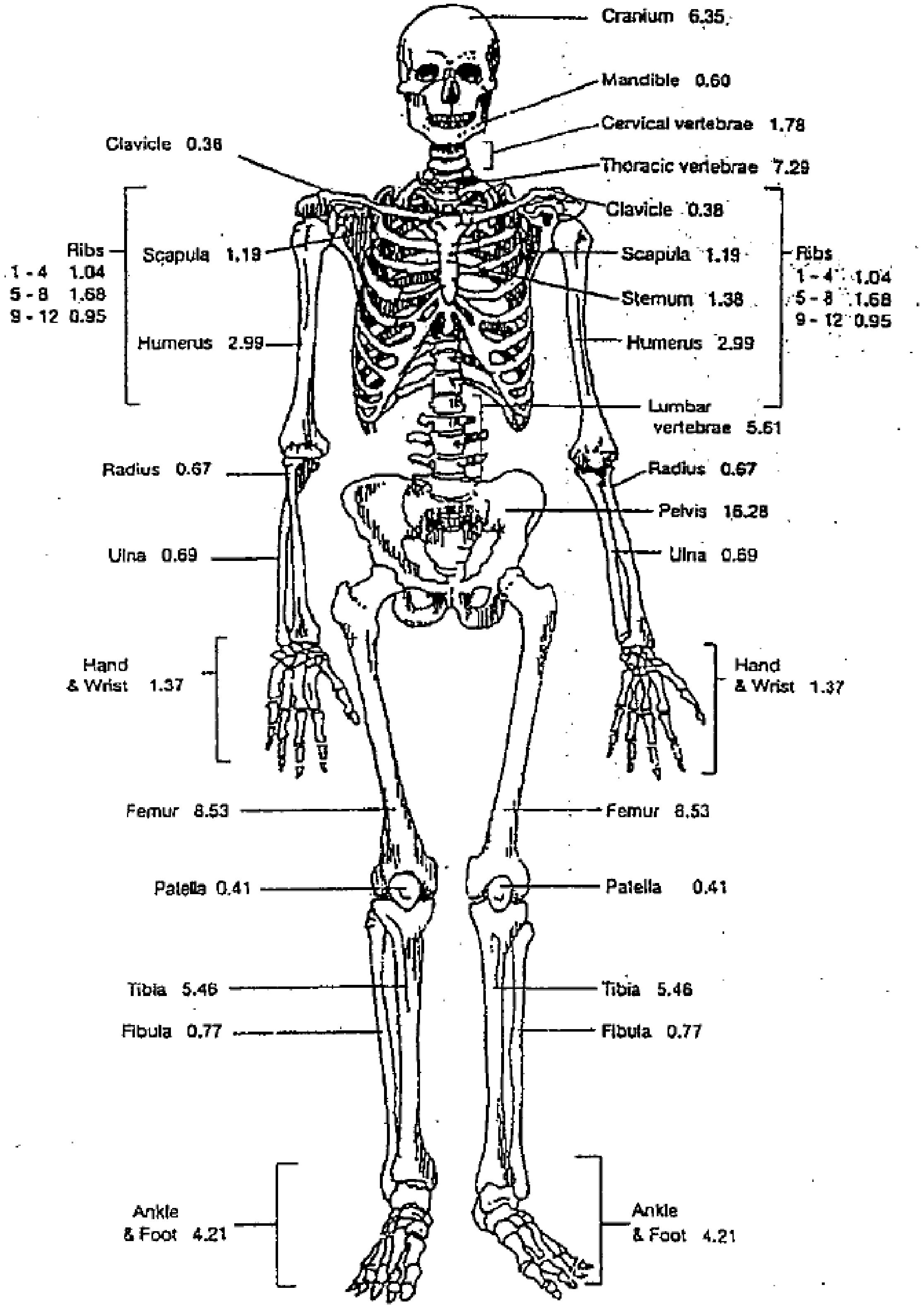

Woodward Holoday E. A summary of the data of Mechanik on the distribution of human bone marrow. Phys Med Biol. 1960;5:57-59
