# Supplementary material for: Multicenter phase II trial of Camrelizumab combined with Apatinib and Eribulin in heavily pretreated patients with advanced triple-negative breast cancer
Source: Nat Commun. 2022 May 31;13:3011. doi: 10.1038/s41467-022-30569-0 (PMC9156739; doi:10.1038/s41467-022-30569-0)
Supplement: Supplementary file 2 — Reporting Summary [file 41467_2022_30569_MOESM2_ESM.pdf]

nature portfolio

Corresponding author(s): Erwei Song

Last updated by author(s): May 1, 2022

Reporting Summary

Nature Portfolio wishes to improve the reproducibility of the work that we publish. This form provides structure for consistency and transparency in reporting. For further information on Nature Portfolio policies, see our Editorial Policies and the Editorial Policy Checklist.

Statistics

For all statistical analyses, confirm that the following items are present in the figure legend, table legend, main text, or Methods section.

n/a	Confirmed	
☐	☒	The exact sample size (*n*) for each experimental group/condition, given as a discrete number and unit of measurement
☐	☒	A statement on whether measurements were taken from distinct samples or whether the same sample was measured repeatedly
☐	☒	The statistical test(s) used AND whether they are one- or two-sided<br>*Only common tests should be described solely by name; describe more complex techniques in the Methods section.*
☐	☒	A description of all covariates tested
☐	☒	A description of any assumptions or corrections, such as tests of normality and adjustment for multiple comparisons
☐	☒	A full description of the statistical parameters including central tendency (e.g. means) or other basic estimates (e.g. regression coefficient) AND variation (e.g. standard deviation) or associated estimates of uncertainty (e.g. confidence intervals)
☐	☒	For null hypothesis testing, the test statistic (e.g. *F*, *t*, *r*) with confidence intervals, effect sizes, degrees of freedom and *P* value noted<br>*Give P values as exact values whenever suitable.*
☒	☐	For Bayesian analysis, information on the choice of priors and Markov chain Monte Carlo settings
☒	☐	For hierarchical and complex designs, identification of the appropriate level for tests and full reporting of outcomes
☐	☒	Estimates of effect sizes (e.g. Cohen's *d*, Pearson's *r*), indicating how they were calculated

Our web collection on statistics for biologists contains articles on many of the points above.

Software and code

Policy information about availability of computer code

Data collection	There was no custom code developed for this project, but all code and statistical packages used for the study will be provided upon request. For database searching of MS data in proteomics, the DIA data was searched against the human UniProt database (20,365 sequences) using Spectronaut software (version 14.5.200813.47784).
Data analysis	Statistical analysis was performed using STATA 12.0 (Stata Co., College Station, TX), R studio (version 4.1.2) and Kaluza Analysis (version 2.0). The area under the curve (AUC), unsupervised clustering, heatmap, principal component analysis (PCA), box plots, scatter plots and KM curve were all constructed using custom R scripts and GraphPad Prism 7.0.

For manuscripts utilizing custom algorithms or software that are central to the research but not yet described in published literature, software must be made available to editors and reviewers. We strongly encourage code deposition in a community repository (e.g. GitHub). See the Nature Portfolio guidelines for submitting code & software for further information.

Data

Policy information about availability of data

All manuscripts must include a data availability statement. This statement should provide the following information, where applicable:

- Accession codes, unique identifiers, or web links for publicly available datasets
- A description of any restrictions on data availability
- For clinical datasets or third party data, please ensure that the statement adheres to our policy

Source data are provided with this paper. The study protocol is available as Supplementary Note 1 in the Supplementary Information file. The proteomics data generated in this study have been deposited in the iProX database under ID IPX0004386001. The authors declare that the data supporting the findings of this study are available within the paper and its supplementary information files. Clinical data can be accessed on request from the corresponding author Song for ten years,

individual de-identified participant data will be shared. The data are not publicly available due to involving patient privacy.

Field-specific reporting

Please select the one below that is the best fit for your research. If you are not sure, read the appropriate sections before making your selection.

☒ Life sciences ☐ Behavioural & social sciences ☐ Ecological, evolutionary & environmental sciences

For a reference copy of the document with all sections, see nature.com/documents/nr-reporting-summary-flat.pdf

Life sciences study design

All studies must disclose on these points even when the disclosure is negative.

Sample size	Simon's two-stage design was used. The null hypothesis of ORR was 26% based on previously reported data of second- or later-line eribulin chemotherapy in patients with advanced TNBC from a randomized controlled trial. The alternative hypothesis of ORR was 46%. According to the two-sided test of 0.05 and the power of 0.80, 14 patients needed to be enrolled in the first stage. If 5 or more patients reached ORR in stage I, another 32 patients would be included in stage II. If more than 16 responders are observed in 46 patients, it has clinical significance.
Data exclusions	No data were excluded from the analysis.
Replication	Biomarkers analysis done on blood samples or biopsies were performed on a single sample due to insufficient material.
Randomization	No randomization. This was a single-arm trial with no comparator arm. Therefore, this study did not involve randomization.
Blinding	This was a single-arm study, therefore no blinding was done.

Reporting for specific materials, systems and methods

We require information from authors about some types of materials, experimental systems and methods used in many studies. Here, indicate whether each material, system or method listed is relevant to your study. If you are not sure if a list item applies to your research, read the appropriate section before selecting a response.

Materials & experimental systems

n/a	Involved in the study	
☐	☒	Antibodies
☒	☐	Eukaryotic cell lines
☒	☐	Palaeontology and archaeology
☒	☐	Animals and other organisms
☐	☒	Human research participants
☐	☒	Clinical data
☒	☐	Dual use research of concern

Methods

n/a	Involved in the study	
☒	☐	ChIP-seq
☐	☒	Flow cytometry
☒	☐	MRI-based neuroimaging

Antibodies

Antibodies used	All the antibodies were from commercial sources and had been validated by the vendors and their validation data were available on the manufacturer's website. Antibodies used for TLS analysis were primary rabbit anti-human CD4 antibody, (Abcam, catalog number ab133616, 1:500 dilution), primary rabbit anti-human CD8 antibody, (Abcam, catalog number ab93278, 1:4000 dilution), and primary rabbit anti-human CD20 antibody, (Abcam, catalog number ab78237, 1:50 dilution). PD-L1 expression of the tumor samples was measured using the FDA-cleared 22C3 assay on the Dako Link 48 platform (DAKO, clone number 22C3, 1:50 dilution).CD8 immunofluorescence were performed on tumor slides using primary rabbit anti-human CD8 (Cat# MA5-14548, Thermo Fisher; 1:200) antibody.
Validation	All antibodies used in this study were obtained from commercial sources and validated according to manufacturers' instruction.

Human research participants

Policy information about studies involving human research participants

Population characteristics	Patients' demographic characteristics were described in detail in Table 1 of this manuscript.
Recruitment	Eligible patients include women age of 18-70 years with unresectable recurrent or metastatic TNBC defined by the American Society of Clinical Oncology/College of American Pathologists; with measurable disease according to the Response Evaluation Criteria In Solid Tumors (RECIST) version 1.1; progressed after prior anthracycline and taxane, with at least one line of unsuccessful systemic therapy in the advanced setting; an Eastern Cooperative Oncology Group (ECOG) status of 0 or 1; and retained adequate organ and bone marrow function. Key exclusion criteria included clinically symptomatic central nerve system metastasis; and history of anti-CTLA-4, TIM3, LAG3, or T cell co-stimulation therapy (prior use of anti-PD-1/PD-L1 antibody was permitted); history of anti-angiogenic drugs or eribulin; and history of autoimmune disease.<br>All patients provided written informed consent before enrollment. All patients who met the inclusion and exclusion criteria were included in this study. There was no potential self selection bias.
Ethics oversight	Study protocol was approved by the Research Ethics Board of Sun Yat-sen Memorial Hospital, the First Affiliated Hospital of Sun Yat-sen University, and Changhai Hospital of Shanghai. This study was conducted in accordance with the Declaration of Helsinki. All patients provided written informed consent before enrollment.

Note that full information on the approval of the study protocol must also be provided in the manuscript.

Clinical data

Policy information about clinical studies

All manuscripts should comply with the ICMJEguidelines for publication of clinical research and a completedCONSORT checklist must be included with all submissions.

Clinical trial registration	NCT04303741
Study protocol	The trial protocol is available from the Supplementary Information.
Data collection	From March 27, 2020, to May 27, 2021, 46 patients were enrolled from three academic hospitals in China. Including Sun Yat-sen Memorial Hospital, the First Affiliated Hospital of Sun Yat-sen University, and Changhai Hospital of Shanghai.
Outcomes	The primary endpoint was ORR per RECIST 1.1, defined as the proportion of patients with best response of complete or partial response. Secondary endpoints included incidence of TRAEs, disease control rate (DCR, proportion of patients with complete response [CR], partial response [PR] or stable disease [SD]), clinical benefit rate (CBR, proportion of patients with CR, PR or durable [≥ 24 weeks] SD), duration of response (DoR, time from the first documented CR or PR to disease progression or any-cause death), time to response (TTR, time from the initiation of study treatment to the first documented CR or PR), PFS (time from the initiation of study treatment to disease progression or any-cause death), one-year OS rate (proportion of patients alive at 1 year), and potential biomarkers.

Flow Cytometry

Plots

Confirm that:

- [x] The axis labels state the marker and fluorochrome used (e.g. CD4-FITC).
- [x] The axis scales are clearly visible. Include numbers along axes only for bottom left plot of group (a 'group' is an analysis of identical markers).
- [x] All plots are contour plots with outliers or pseudocolor plots.
- [x] A numerical value for number of cells or percentage (with statistics) is provided.

Methodology

Sample preparation	For T cell subsets, natural killer (NK) cell and B cell detection, 50 µl peripheral blood was stained with BD Multitest 6-color TBNK Reagent (BD Biosciences), all were in 1:2 dillution, details were as follow: PC7-conjugated anti-CD4 (colon number SK3), APC-conjugated anti-CD8 (colon number SK1) or with FITC-conjugated anti-CD3 (colon number SK7), PE-conjugated anti-CD16 (colon number B73.1)/anti-CD56 (colon number NCAM16.2) and PC7-conjugated anti-CD45 (colon number 2D1) for 30 min at 4℃, respectively. For Tregs detection, whole blood was stained with FITC conjugated anti-CD4 (Beckman, colon number 13B8.2, 1:2 dillution), PC5-conjugated anti-CD25 (Beckman, colon number B1.49.9, 1:2 dillution) and PE-conjugated anti-CD127 (Beckman, colon number R34.34, 1:2 dillution) for 45 min at 4℃. After antibody staining, hemolysin was used to lyse red blood cell (RBC). Single cell suspension was washed and then resuspended with 200 µl staining buffer for flow cytometry.
Instrument	Flow cytometry was performed and analyzed on FACS Diva (BD Bioscience).
Software	Kaluza Analysis (version 2.0)

Cell population abundance	Whole peripheral blood mononuclear cells were used for staining. No purifications or enrichment.
Gating strategy	Cells were visualized by FSC/SSC and the lymphocyte population was gated on. Individual subsets were gated based on their expression of relevant markers. Positive and negative populations were identified using FMO controls.

[x] Tick this box to confirm that a figure exemplifying the gating strategy is provided in the Supplementary Information.
