# Supplementary material for: Multicenter phase II trial of Camrelizumab combined with Apatinib and Eribulin in heavily pretreated patients with advanced triple-negative breast cancer
Source: Nat Commun. 2022 May 31;13:3011. doi: 10.1038/s41467-022-30569-0 (PMC9156739; doi:10.1038/s41467-022-30569-0)
Supplement: Supplementary file 3 — Description of Additional Supplementary Files [file 41467_2022_30569_MOESM3_ESM.pdf]

Title: Supplementary Data 1
Description: FFPE proteomic analysis

Title: Supplementary Data 2
Description: Olink analysis
